# A Triazole‐Substituted Aryl Iodide with Omnipotent Reactivity in Enantioselective Oxidations†

**DOI:** 10.1002/anie.201912023

**Published:** 2019-12-12

**Authors:** Ayham H. Abazid, Boris J. Nachtsheim

**Affiliations:** ^1^ Institut für Organische und Analytische Chemie Universität Bremen Leobener Straße 7 28359 Bremen Germany

**Keywords:** asymmetric oxidation, chiral hypervalent iodine compounds, organocatalysis, oxidation, stereoselective synthesis

## Abstract

A widely applicable triazole‐substituted chiral aryl iodide is described as catalyst for enantioselective oxidation reactions. The introduction of a substituent in ortho‐position to the iodide is key for its high reactivity and selectivity. Besides a robust and modular synthesis, the main advantage of this catalyst is the excellent performance in a plethora of mechanistically diverse enantioselective transformations, such as spirocyclizations, phenol dearomatizations, α‐oxygenations, and oxidative rearrangements. DFT‐calculations of in situ generated [hydroxy(tosyloxy)iodo]arene isomers give an initial rational for the observed reactivity.

## Graphical abstract

**A good all‐rounder**: An easy to synthesize chiral triazole‐substituted aryl iodide is described as an omnipotent catalyst for a variety of iodane‐mediated enantioselective oxidations. It outperforms all existing chiral hypervalent iodine precursors.

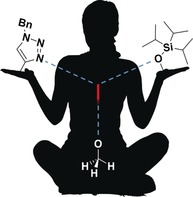

Hypervalent iodine compounds are versatile oxidants which have been utilized with great success in a plethora of oxidative coupling reactions^1^ and in natural product synthesis.^2^ In related enantioselective processes, a chiral aryl iodide precursor can be used in catalytic amounts in combination with a terminal co‐oxidant to generate a chiral hypervalent iodine compound in situ. This chiral oxidant is subsequently capable of transferring its chirality onto the desired coupling products through diastereotopic transition states in the key oxidative C–X bond forming step.^3^

Since the discovery of the first enantioselective transformation catalyzed by a chiral aryl iodide in 2007 by Wirth and co‐workers,^4^ more than a dozen highly diverse C1‐ and C2‐symmetric chiral aryl iodides have been developed.^5^ Successful catalysts, such as **1**–**4** (Figure 1), usually show a good reactivity and selectivity in only one distinct class of oxidative transformation. So far there is no omnipotent chiral aryl iodide available that performs well throughout the most important oxidative transformations and hence can be seen as broadly applicable catalyst for iodane‐based enantioselective couplings. Our group is heavily interested in the development of *N*‐heterocycle‐stabilized iodanes (NHIs) as a new class of stable and at the same time highly reactive hypervalent iodine compounds.^6^, ^7^ With the aim to design novel chiral aryl iodides which are robust to synthesize in a modular sequence and show a good performance throughout a diverse range of enantioselective oxidations, we recently developed the novel triazole‐substituted aryl iodide **5** (Figure 2) and evaluated its reactivity in the Kita‐spirocyclization of 1‐naphthols.^8^

**Figure 1:**
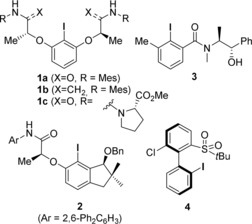
Best‐performing chiral aryl iodide catalysts for enantioselective oxygenations.

**Figure 2:**
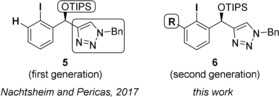
Structure of first‐ and second‐generation triazole catalysts.

Even though this “first‐generation” catalyst gave the so far highest enantioselectivities in direct comparison to other C1‐symmetric aryl iodides for this reaction, reactivities were low. Well‐established C2‐symmetric aryl iodides, such as spirobiindanes developed by Kita or resorcinol ethers **1** developed by Uyanik and Ishihara, showed significantly higher stereoinduction and yielded the desired chiral lactones in better yields.^9^ Due to the promising initial results with catalyst **5** and its highly modular and robust synthesis, we further developed “second‐generation” triazole catalysts **6** (Figure 2) bearing a simple *ortho*‐modification at the aryl iodide. We therefore synthesized *ortho*‐Cl, ‐Me, and ‐OMe‐substituted derivatives **6 a**–**c** (Scheme 1). Their synthesis is straightforward and was completed on a gram scale for each chiral triazole starting from the iodinated carbaldehydes **7 a**–**c**. Addition of ethynylmagnesium bromide gave the racemic propargylic alcohols **8 a**–**c**, which were treated with the esterase CALB and isopropenyl acetate to give the enantiopure alcohols **ent 8 a**–**c** in excellent selectivity. Since both products of the kinetic resolution, the free alcohol and the enantiomeric acetate, can be separated effortlessly, this route gives an efficient access to both enantiomers of the final catalysts. Copper‐mediated Huisgen 1,3‐dipolar cycloaddition of the free alcohols with benzyl azide gives triazoles **9 a**–**c**. The final catalysts **6 a**–**c** were observed through final TIPS‐protection.

**Scheme 1:**
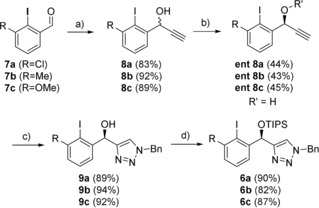
Catalyst synthesis. Reaction conditions: a) **7** (1 equiv), ethynylmagnesium bromide (1.25 equiv) at 0 °C for 2.5 h. b) **8** (1 equiv), CALB (6 mg/mmol of **8**), isopropenyl acetate (1.5 equiv) in toluene at room temperature for 3 days c) **ent 8** (1 equiv), benzyl azide (1.3 equiv), TTMCuCl (0.005 equiv) in water at room temperature for 17 h. d) **9** (1 equiv), 2,6‐lutidine (2 equiv), trialkylsilyl trifluoromethanesulfonate (1.2 equiv), in DCM at 0 °C for 6 h. The starting aldehydes are known in the literature and commercially available. For detailed synthetic procedures, see the Supporting Information.

Having these second‐generation catalysts in hand, we investigated their performance in the Kita‐spirocyclization of 1‐naphthol carboxylic acid **10** (Table 1). In comparison to **5**, the *ortho*‐Cl‐substituted catalyst **6 a** had a higher reactivity yielding the spirolactone (*R*)‐**11** with improved yields after shorter reaction times but with a slightly lower enantioselectivity. Ortho‐Me‐substituted catalyst **6 b** was more reactive than **5** and **6 a**, which correlated with an increased enantioselectivity of 78 % *ee*. The highest reactivity and enantioselectivity was observed with OMe‐substituted catalyst **6 c** giving **11** in 81 % yield and 82 % *ee*. We further optimized the reaction conditions and found that a 1:1 mixture of DCM/CHCl_3_ and addition of ethanol increased the enantioselectivity of **11** to 99 % *ee* (Table 1, entry 6). These are the highest enantioselectivities ever observed in this representative model reaction. **6 c** even outperforms all of the well‐established C2‐symmetric chiral aryl iodides, such as the C2‐symmetric resorcinols **1 a** and **1 b**.^10^ Surprised by the significantly increased reactivity based on this simple synthetic modification we wanted to evaluate the general applicability of **6 c** in other, more challenging, oxidative C–O bond forming reactions (Scheme 2). First, we investigated the oxidative dearomatization of 4‐substituted phenols to *para*‐quinols. Even with sterically highly demanding aryl iodide catalysts, this reaction gives low enantioselectivities due to the high distance between the phenolate, which is bound to the hypervalent iodine center, and the hydroxylated C4. Even the successful resorcinol‐derived aryl iodides **1** give only moderate selectivity of up to 50 %. In 2018, Maruoka and co‐workers introduced a specially designed C1‐symmetric indanol‐based aryl iodide **2**, which was so far the best performing catalyst for this reaction.^11^ We were pleased to find that with 10 mol % **6 c**, 2‐bromo‐4‐methylphenol **12** could be efficiently dearomatized into the 2‐bromo *para*‐quinol **13** in 92 % yield and 93 % *ee*, again a novel best mark for this transformation (Scheme 2 a).

**Scheme 2:**
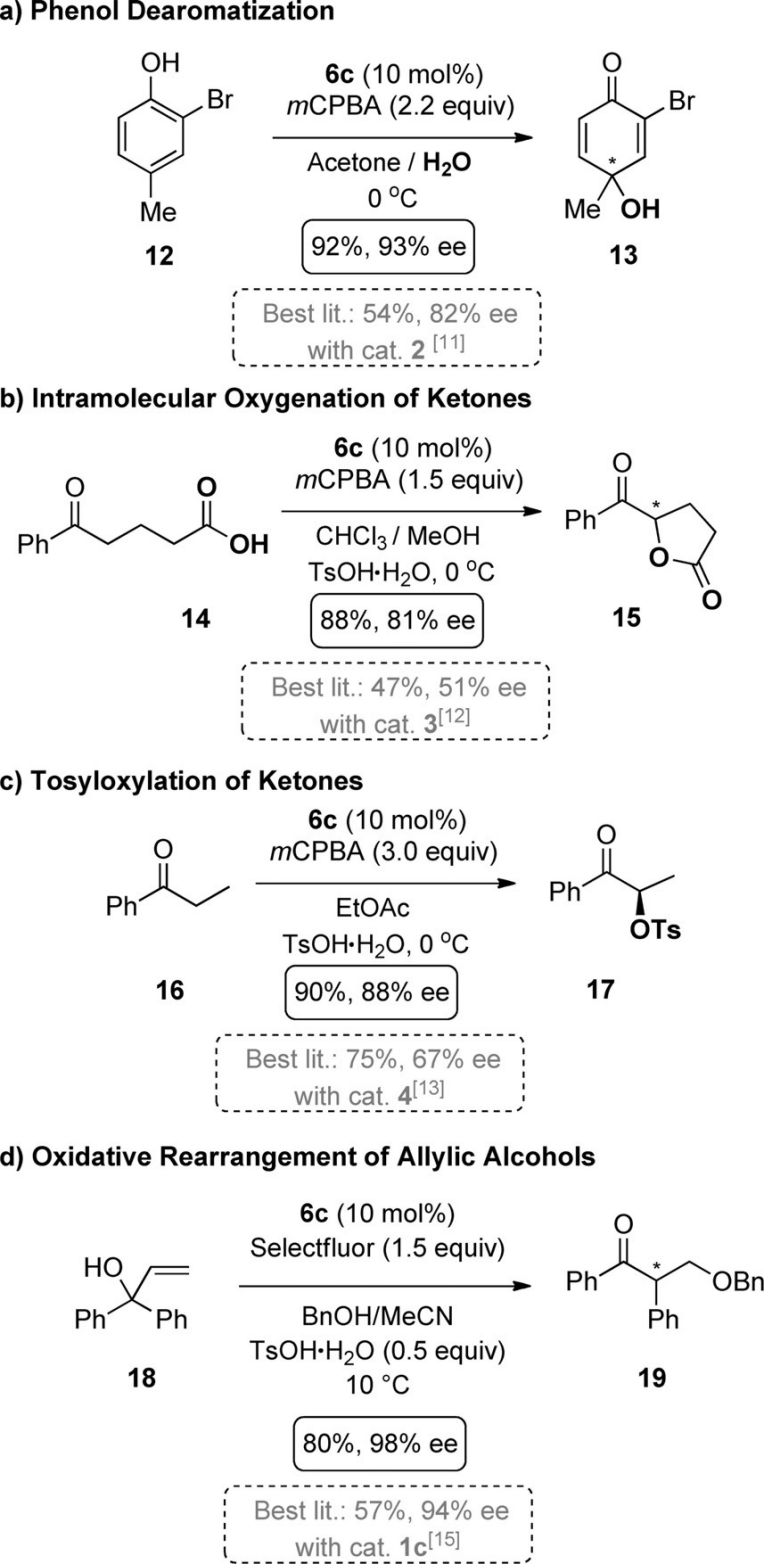
Enantioselective oxygenations and rearrangements catalyzed by **6 c** in comparison with the best literature values. Detailed optimizations for each reaction are discussed in the Supporting Information. Due to missing comparable literature samples, the absolute configuration of the major isomers for **13**, **15**, and **19** has not been finally determined.

**Table 1:** Optimization of the Kita‐spirolactonization. 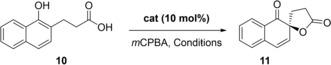

Entry^[a]^	Triazole	Solvent	*T* [°C]	*t* [h]	Yield^[b]^ [%]	*ee* ^[c]^ [%]
1	**5**	DCM	0	36	36	64
2	**6 a**	DCM	0	32	54	60
3	**6 b**	DCM	0	24	62	78
4	**6 c**	DCM	0	22	81	82
5	**6 c**	DCM/CHCl_3_	0	24	85	97
6	**6 c**	**DCM/CHCl_3_ + 6 equiv EtOH**	**−10**	**32**	**85**	**99**

[a] Reaction conditions: **10** (0.09 mmol), cat (0.009 mmol, 10 mol %), *m*CPBA (0.12 mmol, 1.3 equiv), solvent (0.02 m). [b] Isolated yield after column chromatography. [c] Determined by chiral HPLC.

We then investigated the intramolecular α‐oxygenation of 5‐oxo‐5‐phenylpentanoic acid **14** to 5‐benzoyldihydrofuran‐2(3*H*)one **15**. Here, the C1‐symmetric pseudoephedrine‐substituted aryl iodide **3**, as developed by Moran, gave the best results so far (47 % yield and 51 % *ee*).^12^ Again, **6 c** is a superior catalyst for this transformation and yielded the desired lactone **15** in 88 % yield and 81 % *ee* (Scheme 2 b). We then tested the performance of **6 c** in the α‐tosyloxylation of propiophenone. This was the first oxygenation to be catalysed by a chiral aryl iodide catalyst as developed by Wirth and co‐workers in 2007.^4^ However, even after more than a decade of this first enantioselective report, numerous efforts to design efficient chiral aryl iodide catalysts did not result in synthetically adequate enantioselectivities due to hard to control concurring S_N_2‐and S_N_2′‐based reaction pathways. In 2017, Masson and co‐workers developed the sulfone‐containing aryl iodide **4** as the best performing catalyst for this reaction giving **17** in 67 % *ee*.^13^ Again, **6 c** showed superior results in this transformation. A combination of 10 mol % **6 c** and *m*CPBA as co‐oxidant resulted in an efficient conversion of propiophenone **16** to **17** in 90 % yield and 88 % *ee* (Scheme 2 c). Apart from oxygenations, hypervalent iodine compounds can be applied efficiently in enantioselective rearrangements.^14^ Very recently, Gong and co‐workers developed the oxidative rearrangement of allylic alcohols to β‐oxygenated ketones with the lactic‐acid‐derived catalyst **1 c**.^15^ We utilized this useful reaction as another benchmark for **6 c** (Scheme 2 d). Treatment of the tertiary allyl alcohol **18** with 10 mol % **6 c**, Selectfluor as co‐oxidant, and benzyl alcohol, resulted in the formation of the rearranged β‐oxygenated ketone **19** in 80 % yield and 98 % *ee*.

After catalyst **6 c** showed this superior performance among a variety of mechanistically diverse transformations, we decided to gain a better rational about the underlying structural aspects that cause the high reactivity of **6 c**, in particular in direct comparison to the first‐generation catalyst **5**.

It was of particular interest, which secondary bonding interactions stabilize the oxidized hydroxy iodobenzene. After initial oxidation, the iodane can be further stabilized either by an oxygen lone pair of the TIPS‐protected benzyl alcohol or by N3 of the triazole giving two potential isomers. Both isomers could further react in distinct ligand exchange and oxidation pathways and this competition should result in diminished yields and enantioselectivities. To confirm this finding for **6 c** and to verify the importance of an N–I interaction for the performance of this catalyst we synthesized the *N*‐methyl triazolium tetrafluoroborate **6 d** by treatment of **6 c** with Meerwein salt (Scheme 3 a). This catalyst was then used in the Kita‐spirolactonization. Here we found that **6 d** has a diminished reactivity compared to **6 c** giving the spirolactone in only 32 % yield and 61 % *ee* (Scheme 3 b). This poor reactivity is comparable to the reactivity of the first‐generation catalyst **5**. Legault and co‐workers and our group recently demonstrated that a stabilization of cationic iodane species by covalently attached *N*,*O*‐heterocycles, such as oxazoles and oxazolines, is favored through dative N–I bonding interactions as indicated by N–I distances being significantly shorter than O–I distances in calculated structures.^16^ In solid‐state structures only the N‐bound intermediates were observed.^6^

**Scheme 3:**
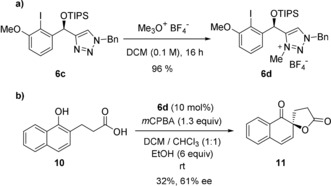
a) Synthesis of *N*‐methyl triazole **6 d**. b) Evaluation of the performance of **6 d** in the Kita‐spirolactonization.

Even though the N‐methylation experiment is a first hint for an intramolecular N–I interaction in the active species of **6 c**, it does not explain the observed effect of the *ortho*‐substituent. Besides the donor ligand, the angle between the plane of the iodoarene and the I–OH bond is an important structural feature which is heavily influenced by *ortho*‐substituents, as described by Legault and co‐workers.^17^ Since all attempts to produce suitable crystals for X‐ray analysis were not successful, DFT calculations were performed for a deeper structural understanding of the catalyst structure and its reactivity.^18^ Energy minima of oxidized [hydroxy(tosyloxy)iodo]arenes **5‐OH** and **6 c‐OH** were searched by preoptimization with Grimme's tight‐binding method GFN2‐xTB^19^ and further fine optimized on a PBEh‐3c/ma‐def2‐SVP(O,N)/def2‐TZVP(I) level of theory.^20^ Single point energies were calculated with the double hybrid PWPB95‐D3 functional together with ma‐def2‐TZVP(O,N)/def2‐TZVPP(I) basis sets.^21^ Preoptimization with GFN2‐xTB resulted in four distinct energy minima for each catalyst (Figure 3). In good agreement with theoretical investigations by Legault et al., initial calculations based on dissociated species with a separated tosylate anion and an iodonium cation revealed that the dissociation process is highly endergonic. (Supporting Information, Scheme S1).^22^ Therefore, dissociated iodonium complexes were not further considered. Minimized structures of **5‐OH** are summarized in Figure 3 a, minimized structures of **6 c‐OH** are summarized in Figure 3 b. For both catalysts two isomers (**5/6 c‐OH‐N** and **5/6 c‐OH‐O**) show a direct stabilization of the hypervalent iodine atom through dative N–I or O–I interactions.

**Figure 3:**
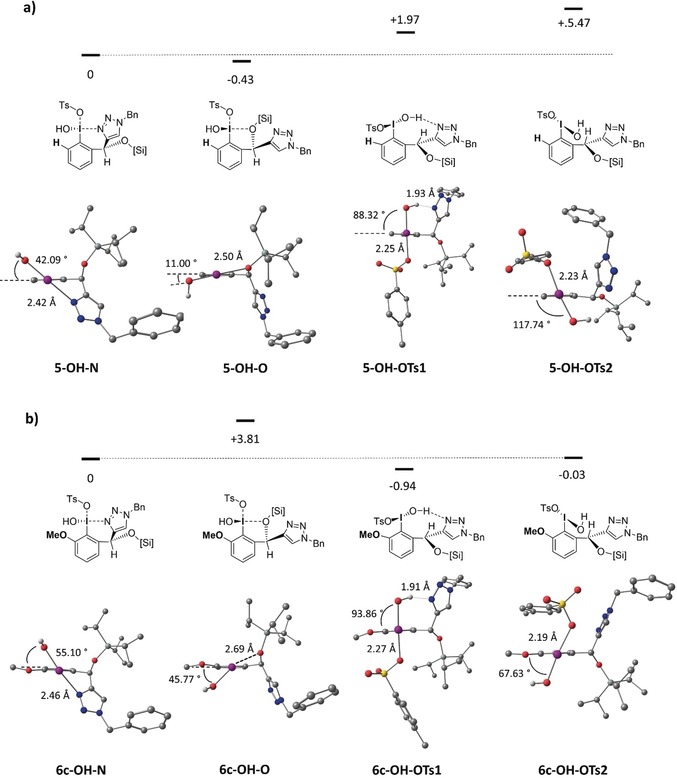
Calculated structures and relative free energies (G in kcal mol^−1^) of hydroxy(tosyloxy) iodobenzene isomers of **5‐OH** (a) and **6 c‐OH** (b). For isomers **5/6 c‐OH‐N** and **5/6 c‐OH‐O** the tosylate counterion has been omitted in the illustration. All non‐heteroatom‐bound hydrogens have been omitted as well. Structure optimization and frequency analysis was performed on the PBEh‐3c/ma‐def2‐SVP(O,N)/def2‐TZVP(I) level of theory. Final single‐point energies were calculated on a PWPB95/ma‐def2‐TZVP(O,N)/def2‐TZVPP(I) level of theory using a continuum solvation model (CPCM) in CHCl_3_.

These oxidized isomers are nearly equal in energy for **5‐OH**, whereas for **6 c‐OH** the N‐bound isomer is favored by 3.81 kcal mol^−1^. In these isomers the tosylate counterion (not shown) is usually bound in an apical position with typical TsO–I bond length of more than 2.6 Å. In the other observed isomers (**5/6 c‐OH‐OTs1** and **5/6 c‐OH‐OTs2**) the tosylate acts as the key ligand building the linear hypervalent bond in the oxidized state together with the OH group (TsO–I bond length of 2.19–2.25 Å). In **5/6 c‐OH‐OTs1** the hydroxy group is located *cis* to the triazole ring and is engaged in hydrogen bonding with the *N*‐heterocycle. For **6 d** this hydrogen bond is not operational and therefore this isomer would be destabilized. Furthermore, the oxidation with *m*CPBA should be favored through an initial hydrogen bonding between the triazole and the transferred terminal hydroxy group of the peracid.

However, for isomer **5/6 c‐OH‐OTs2**, the hydroxy ligand is located *trans* to the triazole ring and hence no further intramolecular secondary interactions can be observed. For **5‐OH** the TsO‐bound isomers are significantly higher in energy (+1.97 and +5.47 kcal mol^−1^) compared to the triazole‐bound isomer **5‐OH‐N**. In contrast, the TsO‐stabilized isomers of **6 c** are equal or slightly lower in energy compared to **6C‐OH‐N**. In particular, the relatively high stability of **6 c‐OH‐OTs1** is remarkable due to the high dihedral angle of 93.86 between C(1)/C(6) of the arene ring and the I–O bond. Usually, linear arrangements are preferred in which the arene is in plane with the hypervalent bond. This can be nearly found in **5‐OH‐O**. This isomer has the smallest dihedral angle of 11.00°. A high dihedral angle between the arene ring and the hypervalent bond, defined by Legault and co‐workers as “hypervalent twist”,^16^ together with the good leaving‐group ability of the tosylate ligand as found in **6 c‐OH‐OTs1** are crucial for further ligand exchange reactions with phenolic or enolic oxygens to initiate the discussed enantioselective coupling reactions. We are therefore confident that these calculations give a good initial rational for the high reactivity of catalyst **6 c** and are an ideal starting point for further catalyst improvements and theoretical investigations concerning the underlying mechanisms of each investigated enantioselective transformation.

In summary, the triazole‐substituted aryl iodide **6 c** is the most versatile chiral aryl iodide catalyst that has been developed so far for enantioselective reactions based on in situ generated hypervalent iodine compounds. This single catalyst shows a remarkable reactivity in the Kita‐spirocyclizaton, the α‐tosyloxylation of propiophenone, oxidative lactonizations, and in the oxidative rearrangement of allyl alcohols. To our knowledge the observed enantioselectivity for every investigated reaction is the highest ever reported and hence this catalyst can be defined as omnipotent. The initial DFT calculations indicate a significant role of the triazole as a stabilizing donor both in a potential N‐bound state or as a hydrogen‐bond acceptor in a [hydroxy(tosyloxy)iodo]arene derivative. This forces the geometry of the hypervalent iodine centre into a reactive bent state with an unusual vertical alignment between the hypervalent bond and the arene. In‐depth theoretical investigations are now necessary for every investigated reaction to fully explain the observed omnipotence. With this information rational fine tuning of this highly modular catalyst for further enantioselective oxidations will be possible.

## Conflict of interest

The authors declare no conflict of interest.

## Supporting information

As a service to our authors and readers, this journal provides supporting information supplied by the authors. Such materials are peer reviewed and may be re‐organized for online delivery, but are not copy‐edited or typeset. Technical support issues arising from supporting information (other than missing files) should be addressed to the authors.

Supplementary
