# Supplementary material for: A Triazole‐Substituted Aryl Iodide with Omnipotent Reactivity in Enantioselective Oxidations
Source: Angew Chem Int Ed Engl. 2019 Dec 12;59(4):1479–84. doi: 10.1002/anie.201912023 (PMC7003988; doi:10.1002/anie.201912023)
Supplement: Supplementary file 1 — Supplementary [file ANIE-59-1479-s001.pdf]

Angewandte Chemie
Eine Zeitschrift der Gesellschaft Deutscher Chemiker

Supporting Information

A Triazole-Substituted Aryl Iodide with Omnipotent Reactivity in Enantioselective Oxidations**

*Ayham H. Abazid and Boris J. Nachtsheim**

anie_201912023_sm_miscellaneous_information.pdf

Table of Content

1. General Information S4

2. Synthesis of Chiral Iodoarenes. S5

2.1. Synthesis of propargyl alcohol derivatives (GP1) S5

2.1.1. Synthesis of (S)-1-(3-chloro-2-iodophenyl)prop-2-yn-1-ol (8a) S6

2.1.2. Synthesis of (S)-1-(2-iodo-3-methylphenyl)prop-2-yn-1-ol (8b) S6

2.1.3. Synthesis of (S)-1-(2-iodo-3-methoxyphenyl)prop-2-yn-1-ol (8c) S7

2.2. General Procedure for the Synthesis of 1,4-Triazoles (GP2) S7

2.2.1. Synthesis of (R)-(1-benzyl-1H-1,2,3-triazol-4-yl)(3-chloro-2- iodophenyl) methanol (9a) S7

2.2.2. Synthesis of (R)-(1-benzyl-1H-1,2,3-triazol-4-yl)(2-iodo-3-methyl phenyl)methanol (9b) S8

2.2.3. Synthesis of (R)-(1-benzyl-1H-1,2,3-triazol-4-yl)(2-iodo-3- methoxyphenyl) methanol (9c) S8

2.3. General Procedure for the Synthesis of *O*-Trialkylsilyl- Protected Iodoarenes (GP3) S9

2.3.1. Synthesis of (R)-1-benzyl-4-((3-chloro-2- iodophenyl)((triisopropylsilyl) oxy)methyl)-1H-1,2,3-triazole (6a) S9

2.3.2. Synthesis of (R)-1-benzyl-4-((2-iodo-3- methylphenyl) ((triisopropylsilyl)oxy)methyl)-1H-1,2,3-triazole (6b) S9

2.3.3. Synthesis of (R)-1-benzyl-4-((2-iodo-3 methoxyphenyl) ((triisopropylsilyl) oxy)methyl)-1H-1,2,3-triazole (6c) S10

3. Evaluation the Catalysts in Enantioselective Reactions: S10

3.1. Enantioselective Spirocyclizations S10

3.1.1. Typical Procedure of Enantioselective Kita-Spirocyclization S11

Mixture of enantiomer product with the racemic compound: S12

3.1.2. Synthesis and screening the reactivity of triazolium salt (6d) in Enantioselective Kita Spirocyclizations S13

3.2. Enantioselective 4-Hydroxylation of Phenols S14

3.2.1. Optimization of the Reaction Conditions S14

3.2.2. Typical Procedure of 4-Hydroxylation of Phenols S15

3.3. Enantioselective α-Oxidation of Ketones S16

3.3.1. Optimization of the Reaction Conditions S17

3.3.2. Typical Procedure of α- Oxidation of Ketones S17

3.4. α-Tosyloxylation of Propiophenone S19

3.4.1. Optimization of the Reaction Conditions S19

3.4.2. Typical Procedure of α-*Tosyloxylation* of Propiophenone S20

3.4. Enantioselective Oxidative Rearrangement of Allylic Alcohols S21

3.4.1. Preparation of the allylic alcohol 19 .... S21

3.4.2.Typical Procedure of Enantioselective Oxidative Rearrangement of Allylic Alcohols... ....S22

4. NMR Spectra for New Compounds S24

4.1.NMR of (*S*)-1-(3-chloro-2-iodophenyl)prop-2-yn-1-ol (8a) in $CDCl_3$........S25
4.2.NMR of (*S*)-1-(2-iodo-3-methylphenyl)prop-2-yn-1-ol (8b) in $CDCl_3$........S26
4.3.NMR of (*S*)-1-(2-iodo-3-methoxyphenyl)prop-2-yn-1-ol (8c) in $CDCl_3$........S27
4.4.NMR of of (*R*)-(1-benzyl-1*H*-1,2,3-triazol-4-yl)(3-chloro-2- iodophenyl)methanol (9a) in $CDCl_3$........S28
4.5.NMR of (*R*)-(1-benzyl-1*H*-1,2,3-triazol-4-yl)(2-iodo-3-methyl phenyl) methanol (9b) in $CDCl_3$........S29
4.6.NMR of (*R*)-(1-benzyl-1*H*-1,2,3-triazol-4-yl)(2-iodo-3- methoxyphenyl) methanol (9c) in $CDCl_3$........S30
4.7.NMR of (*R*)-1-benzyl-4-((3-chloro-2- iodophenyl)((triisopropylsilyl) oxy)methyl)-1*H*-1,2,3-triazole (6a) in $CDCl_3$........S31
4.8.NMR of (*R*)-1-benzyl-4-((2-iodo-3- methylphenyl) ((triisopropylsilyl) oxy)methyl)-1*H*-1,2,3-triazole (6b) in $CDCl_3$........S32
4.9.NMR of (*R*)-1-benzyl-4-((2-iodo-3 methoxyphenyl) ((triisopropylsilyl) oxy)methyl)-1*H*-1,2,3-triazole (6c) in $CDCl_3$........S33
4.10.NMR of (*R*)-1-benzyl-4-((2-iodo-3-methoxyphenyl)((triisopropylsilyl) oxy)methyl)-3-methyl-1*H*-1,2,3-triazol-3-ium (6d) in $CDCl_3$........S34
4.11.NMR of 3,4-dihydro-1'H,5H-spiro[furan-2,2'-naphthalene]-1',5- (11) in $CDCl_3$..S35
4.12.NMR of 2-bromo-4-hydroxy-4-methylcyclohexa-2,5-dien-1-one (13) in $CDCl_3$ S36
4.13.NMR of 5-benzoyldihydrofuran-2(3H)-one (15) in $CDCl_3$........S37
4.14.NMR of 1-oxo-1-phenylpropan-2-yl 4-methylbenzenesulfonate (17) in $CDCl_3$.S38
4.15.NMR of 1,1-diphenylprop-2-en-1-ol (19) in $CDCl_3$........S39
4.16.NMR of (*S*)-3-(benzyloxy)-1,2-diphenylpropan-1-one (20) in $CDCl_3$........S40

5. HPLC Chromatograms S41

6. CD Spectra S43

7. Computational Studies........S44

7.1. Computational Details........S44
a. Coordinates........S45

8. TD-DFT/TDA Excited states for (R)-11 ....S68

9. References S73

1. General Information

Unless otherwise noted, all reactions were carried out under a nitrogen atmosphere using standard *Schlenk* techniques. All chemicals were purchased from commercial suppliers and either used as received or purified according to *Purification of Common Laboratory Chemicals*. Dry tetrahydrofuran (THF), dichloromethane (CH_2Cl_2) and acetonitrile (MeCN) were obtained from an *inert* PS-MD-6 solvent purification system. All other solvents were dried using standard methods.

Thin layer chromatography was performed on fluorescence indicator marked precoated silica gel 60 plates (*Macherey-Nagel*, ALUGRAM Xtra SIL G/UV_{254}) and visualized by UV light (254 nm/366 nm). Flash column chromatography was performed on silica gel (0.040 – 0.063 mm) with the solvents given in the procedures. Retention factors were determined at chamber saturation at 25 °C. Developments were carried out between 3.0 – 3.5 cm.

NMR spectra were recorded on a *Bruker AVANCE NEO 600 MHz* spectrometer at 20 °C. Chemical shifts for ^{1}H-NMR spectra are reported as *δ* (parts per million) relative to the residual proton signal of $CDCl_3$ at 7.26 ppm (s), or DMSO-d_6 at 2.50 ppm (quin). Chemical shifts for ^{13}C-NMR spectra are reported as *δ* (parts per million) relative to the signal of CDCl3 at 77.0 ppm (t), or DMSO-d_6 at 39.5 ppm (sept). The following abbreviations are used to describe splitting patterns: br. = broad, s = singlet, d = doublet, t = triplet, td = triplet of doublets, tt = triplet of triplets, q = quartet, sept = septet, m = multiplet. Coupling constants *J* are given in Hertz.

ESI and APCI mass spectra were recorded on an *Advion* Expression CMSL *via* ASAP probe or direct inlet. High resolution (HR) EI mass spectra were recorded on a double focusing mass spectrometer ThermoQuest MAT 95 XL from *Finnigan MAT*. HR-ESI mass spectra were recorded on a *Bruker* impact II. All Signals are reported with the quotient from mass to charge m/z.
IR spectra were recorded on a *Nicolet* Thermo iS10 scientific spectrometer with a diamond ATR unit.

Melting points of solids, compounds that solidified after chromatography, were measured on a *Büchi* M-5600 Melting Point apparatus and are uncorrected. The measurements were performed with a heating rate of 2 °C/min and the melting points are reported in °C.

Low temperature reactions were cooled using a *Julabo* FT902 cryostat. If not otherwise noted, solvents were removed on a *Büchi* Rotavapor R-300 with 40 °C water bath temperature.

HPLC chromatograms were recorded on *AGILENT TECHNOLOGIES 1260 INFINITY.* UV detection monitored at 214nm*, used Columns: Reprosil Chiral- OM, 5 µm (250x4,6* mm) and Reprosil Chiral- AM, 5 µm (250x4,6 mm) were purchased from *DR. MAISCH GmbH.* CD-spectra were recorded on a Jasco J-810 CD-Spectrometer at 25 °C in Chloroform with a concentration of 9.2 mmol

2. Synthesis of Chiral Iodoarenes.

2.1. Synthesis of propargyl alcohol derivatives (GP1)

A solution of ethynylmagnesium bromide (1.25 equiv, 0.5M in THF) was cooled to 0 ^{o}C. Subsequently, 2-iodobenzaldehyde derivatives (1.0 equiv.) dissolved in dry THF was added over 0.5 h. The resulting reaction mixture was stirred for an additional 2 h at 0 ^{o}C. Saturated NH_4Cl (100 mL) was added, phases were separated and the aqueous layer was extracted with EtOAc (3x40 mL). The combined organic layers were washed with brine (100 mL), dried over Na_2SO_4 and the solvent was removed under reduced pressure. The crude racemic of propargyl alcohol derivatives **8**(a-c) were used without further purification in the following kinetic resolution. The racemate (1.0 equiv) was dissolved in dry toluene and CALB (6 mg mmol-1), isopropenyl acetate (1.5 equiv.) and Na_2CO_3 (1.0 equiv.) were added successively.[1] The resulting mixture was stirred at room temperature and the progress was monitored by HPLC. After 3 days full conversion of one enantiomer was observed. The reaction mixture was filtered off, washed with toluene and the solvent was removed under reduced pressure. The crude reaction mixture was purified by column chromatography on silica gel using cyclohexane and EtOAc (10:1 to 2:1) to obtain the propargyl acetate (*R*) as well as the free propargyl alcohol **8a-c** (*S*).

2.1.1. Synthesis of (*S*)-1-(3-chloro-2-iodophenyl)prop-2-yn-1-ol (8a)

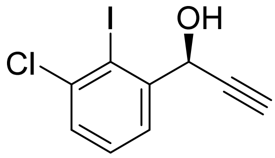

Following GP1, 3-chloro-2-iodobenzaldehyde[2] (5.00 g, 18.8 mmol) was stirred in THF (19 ml) with ethynylmagnesium bromide (46.9 ml, 23.46 mmol) to afford the racemic product of **8a** (4.55 g, 15.6 mmol, **83%**) as a yellow solid. Then the racemic (4.5 g, 15.4 mmol) undergoes the kinetic resolution step by dissolving in dry toluene (29 ml) with CALB (90.0 mg, 6 mg $mmol^{-1}$) and isopropenyl acetate (2.5 ml, 23.1 mmol) and stirring for 3 days to isolate the enantiomer (*S*) of **8a** (2.00 g, 6.80 mmol, **44%**) as a pale-yellow solid. Mp: 29-31 ^{0}C. ^{1}H NMR (600 MHz, Chloroform-d) δ 7.69 (dd, J = 7.7, 1.5 Hz, 1H), 7.48 (dd, J = 7.9, 1.6 Hz, 1H), 7.37 (t, J = 7.8 Hz, 1H), 5.79 (dd, J = 5.2, 2.2 Hz, 1H), 2.71 (d, J = 2.2 Hz, 1H), 2.56 (d, J = 5.3 Hz, 1H). ^{13}C NMR (150 MHz, Chloroform-d) δ 145.1, 139.7, 129.6, 129.4, 125.7, 102.6, 82.3, 75.4, 69.5. HR-MS (EI, 70 eV): calculated for C_9H_6ClIO $[M]^{+\cdot}$: m/z = 291.91520, found: 291.91464 (Dev.: 0.56 mu; 1.91 ppm). IR (ATR): $\tilde{\nu}$ (cm^{-1}) =3292, 3278, 1464, 1205, 1132, 1054, 1025, 960, 758, 645 cm^{-1}. HPLC (OM column, hexane-*i*-PrOH=94:6 as eluent, 1.0 mL/min^{-1}): t=14.318 min, for >99.99% ee.

2.1.2. Synthesis of (*S*)-1-(2-iodo-3-methylphenyl)prop-2-yn-1-ol (8b)

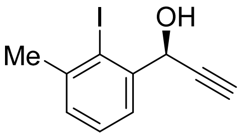

Following GP1, 2-iodo-3-methylbenzaldehyde [3] (4.00 g, 16.3 mmol) was stirred in THF (16.3 ml) with ethynylmagnesium bromide (40.6 ml, 20.32 mmol) to afford the racemic product of **8b** (4.08 g, 15.0 mmol, **92%**) as a white solid. Then the racemic (3.5 g, 12.9 mmol) undergoes the kinetic resolution step by dissolving in dry toluene (24.5 ml) with CALB (77.0 mg, 6 mg $mmol^{-1}$) and isopropenyl acetate (2.1 ml, 19.3 mmol) and stirring for 3 days to isolate the enantiomer (*S*) **8b** (1.51 g, 5.60 mmol, **43%**) as a white solid. Mp: 32-34 ^{0}C. ^{1}H NMR (600 MHz, Chloroform-d) δ 7.60 (dd, J = 7.7, 2.3 Hz, 1H), 7.29 (t, J = 7.7 Hz, 1H), 7.24 (dd, J = 7.9, 2.2 Hz, 1H), 5.82 (dd, J = 5.2, 2.5 Hz, 1H), 2.67 (d, J = 2.3 Hz, 1H), 2.50 (s, 3H). ^{13}C NMR (150 MHz, Chloroform-d) δ 142.7, 142.6, 130.1, 128.4, 125.3, 105.5, 82.9, 75.1, 69.3, 29.7. IR (ATR): $\tilde{\nu}$ (cm^{-1}) =3286, 3264, 1457, 1112, 1123, 1057, 1027, 962, 759, 642 cm^{-1}. HPLC (OM column, hexane-*i*-PrOH=90:10 as eluent, 1.0 mL/min-1): t=5.285 min, for >99.99% ee.

2.1.3. Synthesis of (*S*)-1-(2-iodo-3-methoxyphenyl)prop-2-yn-1-ol (8c)

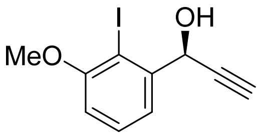

Following GP1, 2-iodo-3-methoxybenzaldehyde[2] (3.50 g, 13.36 mmol) was stirred in THF (13.4 ml) with ethynylmagnesium bromide (33.4 ml, 16.7 mmol) to afford the racemic product of **8c** (3.42 g, 11.9 mmol, **89%**) as a white solid. Then the racemic (3.30 g, 11.5 mmol) undergoes the kinetic resolution step by dissolving in dry toluene (21.8 ml) with CALB (92.0 mg, 6 mg $mmol^{-1}$) and isopropenyl acetate (1.9 ml, 17.2 mmol) and stirring for 3 days to isolate the enantiomer (*S*) **8c** (1.48 g, 5.14 mmol, **45%**) as a white solid. Mp: 33-35 ^{0}C. ^{1}H NMR (600 MHz, Chloroform-d) δ 7.42 (dd, *J* = 7.7, 1.4 Hz, 1H), 7.36 (t, *J* = 7.9 Hz, 1H), 6.82 (dd, *J* = 8.1, 1.4 Hz, 1H), 5.80 (dd, *J* = 5.3, 2.2 Hz, 1H), 5.30 (s, 1H), 3.90 (s, 3H), 2.67 (d, *J* = 2.3 Hz, 1H). ^{13}C NMR (150 MHz, Chloroform-d) δ 158.1, 143.8, 129.7, 120.3, 111.0, 90.6, 82.7, 75.1, 68.7, 56.7. HR-MS (EI, 70 eV): calculated for $C_{10}H_9IO_2$ $[M]^{+\cdot}$: m/z = 287.96418, found: 287.96454 (Dev.: 0.36 mu; 1.25 ppm). IR (ATR): $\tilde{\nu}$ (cm^{-1}) =3255, 3260, 1454, 1183, 1106, 1029, 1002, 928, 737, 629 cm^{-1}. HPLC (OM column, hexane-*i*-PrOH=93:7 as eluent, 1.0 mL/min^{-1}): t=13.835 min, for >99.99% ee.

2.2. General Procedure for the Synthesis of 1,4-Triazoles (GP2)

To a suspension of propargyl alcohol **8a-c** (*S*) (1.0 equiv.) in distilled water (1M) were added benzyl azide (1.3 equiv.) and tris(1- benzyl-1H-1,2,3-triazol-4-yl)methanol copper chloride TTMCuCl (0.005 equiv.).[3] The reaction mixture was vigorously stirred for 17 h at the room temperature. The precipitate formed was dissolved in EtOAc (40 ml), the phases separated and the aqueous layer was extracted with EtOAc (3x20). The combined organic layers were washed with brine, dried over Na_2SO_4 and the solvent was removed under reduced pressure. The crude product was purified by recrystallization from toluene.

2.2.1. Synthesis of (*R*)-(1-benzyl-1*H*-1,2,3-triazol-4-yl)(3-chloro-2- iodophenyl) methanol (9a)

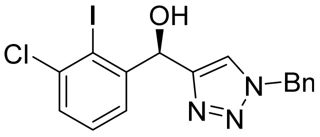

Following GP2, a suspension of enantiomer (*S*) **8a** (1.80 g, 6.29 mmol) in distilled water (6.3 ml) with the benzyl azide (1.00 ml, 8.18 mmol) and TTMCuCl (20.0 mg, 31.0 µmol) was stirring for 17 hours to isolated **9a** (2.38 g, 5.60 mmol, **89%**) as a brownish solid. Mp: 135-137 ^{0}C. ^{1}H NMR (600 MHz, Chloroform-d) δ 7.55 (dd, J = 7.8, 1.6 Hz, 1H), 7.45 (dd, J = 7.9, 1.5 Hz, 1H), 7.39 – 7.32 (m, 5H), 7.27 – 7.23 (m, 2H), 7.21 (s, 1H), 6.31

(d, J = 3.3 Hz, 1H), 5.51 (d, J = 2.7 Hz, 2H). ^{13}C NMR (150 MHz, Chloroform-d) δ 147.0, 139.3, 134.4, 129.5, 129.1, 128.9, 128.8, 128.3, 128.3, 127.9, 125.8, 102.2, 73.5, 54.2. HR-MS (APCI): calculated for $C_{16}H_{13}ClIN_3O$ $[M+H]^+$: m/z =425.97917, found: 425.98619 (Dev.: 0.28 mu; 0.65 ppm). IR (ATR): $\tilde{\nu}$ (cm^{-1})=3486 (br), 3124, 3062, 1440, 1237, 1147, 1072, 1022, 776, 718 cm^{-1}.

2.2.2. Synthesis of (*R*)-(1-benzyl-1*H*-1,2,3-triazol-4-yl)(2-iodo-3-methyl phenyl)methanol (9b)

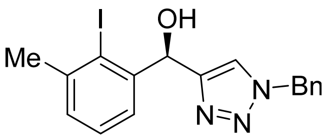

Following GP2, a suspension of enantiomer (*S*) **8b** (1.40 g, 5.15 mmol) in distilled water (5.1 ml) with benzyl azide (0.8 ml, 6.70 mmol) and TTMCuCl (16.0 mg, 26.0 µmol) was stirring for 17 hours to isolated **9b** (1.96 g, 4.84 mmol, **94%**) as off-white solid. Mp: 133-135 ^{0}C. ^1H NMR (600 MHz, Chloroform-d) δ 7.41 (dd, J = 7.6, 1.4 Hz, 1H), 7.37 – 7.32 (m, 3H), 7.27 – 7.24 (m, 1H), 7.23 – 7.17 (m, 4H), 6.34 (s, 1H), 5.48 (d, J = 1.5 Hz, 2H), 3.47 (s, 1H), 2.47 (s, 3H). ^{13}C NMR (150 MHz, Chloroform-d) δ 150.2, 144.4, 142.2, 134.6, 129.5, 129.1, 128.7, 128.3, 127.9, 125.3, 122.0, 105.2, 73.2, 54.2, 29.7. HR-MS (EI, 70 eV): calculated for $C_{17}H_{16}IN_3O$ $[M]^{+\cdot}$: m/z = 405.03382, found: 405.04101 (Dev.: 0.07 mu; 0.18 ppm). IR (ATR): $\tilde{\nu}$ (cm^{-1})=3469 (br), 3105, 3056, 1423, 1225, 1126, 1053, 1015, 763, 717 cm^{-1}.

2.2.3. Synthesis of (*R*)-(1-benzyl-1*H*-1,2,3-triazol-4-yl)(2-iodo-3- methoxyphenyl) methanol (9c)

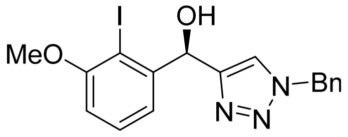

Following GP2, a suspension of enantiomer (*S*) **8c** (1.35 g, 4.69 mmol) in distilled water (4.7 ml) with benzyl azide (0.76 ml, 6.09 mmol) and TTMCuCl (14 mg, 23.0 µmol) was stirring for 17 hours to isolated **9c** (1.82 g, 4.32 mmol, **92%**) as a white solid. Mp: 125-12 ^{0}C. ^1H NMR (600 MHz, Chloroform-d) δ 7.38 – 7.31 (m, 5H), 7.25 – 7.19 (m, 4H), 6.79 (dd, J = 8.2, 1.0 Hz, 1H), 6.32 (s, 1H), 5.48 (s, 2H), 3.89 (s, 3H). ^{13}C NMR (150 MHz, Chloroform-d) δ 157.8, 145.7, 129.7, 129.1, 128.7, 127.9, 123.6, 121.9, 120.3, 110.4, 90.2, 72.8, 56.6, 54.2. A signal is missing due to overlap. HR-MS (APCI)*:* calculated for $C_{17}H_{16}IN_3O_2$ $[M+H]^+$: m/z =422.02869, found: 422.03692 (Dev.: 0.08 mu; 0.19 ppm). IR (ATR): $\tilde{\nu}$ (cm^{-1})=3461 (br), 3102, 3039, 1409, 1203, 1120, 1053, 992, 747, 694 cm^{-1}.

2.3. General Procedure for the Synthesis of *O*-Trialkylsilyl- Protected Iodoarenes (GP3)

The corresponding iodoarene **9a-c** (*R*) (1.0 equiv.) was dissolved in dry DCM (0.15 M) and cooled to 0 °C. Then 2,6-lutidine (2 equiv.) and the corresponding trialkylsilyl trifluoromethanesulfonate (1.2 equiv.) were added consecutively and the reaction mixture was stirred for 6 h at room temperature. Distilled H_2O was added, the phases were separated and the aqueous phase was extracted with DCM (three times). The combined organic layers were washed with brine, dried over Na_2SO_4 and the solvent was removed under reduced pressure. The crude product was purified by column chromatography on silica gel using cyclohexane and EtOAc (20:1 to 7:1).

2.3.1. Synthesis of (*R*)-1-benzyl-4-((3-chloro-2- iodophenyl)((triisopropylsilyl) oxy)methyl)-1*H*-1,2,3-triazole (6a)

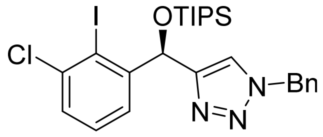

Following GP3, **9a** (2.10 g, 4.93 mmol) was dissolved in dry DCM (32.9 ml) with 2,6-lutidine (1.10 ml, 9.87 mmol) and trialkylsilyl trifluoromethanesulfonate (1.60 ml, 5.92 mmol) was stirring for 16 hours to isolated **6a** (2.58 g, 4.43 mmol, **90%**) as a white solid. Mp: 59 ^{0}C. ^{1}H NMR (600 MHz, Chloroform-d) δ 7.72 (dd, J = 7.8, 1.4 Hz, 1H), 7.40 (dd, J = 7.8, 1.6 Hz, 1H), 7.38 – 7.32 (m, 4H), 7.22 (s, 1H), 7.19 (dd, J = 7.3, 2.1 Hz, 2H), 6.38 (s, 1H), 5.56 – 5.42 (m, 2H), 1.17 (hept, J = 7.5 Hz, 3H), 0.99 (d, J = 7.5 Hz, 18H). ^{13}C NMR (150 MHz, Chloroform-d) δ 150.8, 148.8, 138.8, 134.9, 129.3, 129.0, 128.6, 128.4, 127.7, 125.9, 121.7, 101.6, 74.3, 54.0, 17.91, 12.2. HR-MS (APCI): calculated for $C_{25}H_{33}ClIN_3OSi^+$ $[M+H]^+$: m/z =582.11258, found: 582.11941 (Dev.: 0.48 mu; 0.82 ppm). IR (ATR): $\tilde{\nu}$ (cm^{-1})=3152, 2960, 2876, 1730, 1475, 1382, 1109, 903, 841, 708 cm^{-1}. HPLC (OM column, hexane-i-PrOH=98:2 as eluent, 1.0 mLmin-1): t=17.0 min, for >99.99% ee.

2.3.2. Synthesis of (*R*)-1-benzyl-4-((2-iodo-3- methylphenyl) ((triisopropylsilyl)oxy)methyl)-1*H*-1,2,3-triazole (6b)

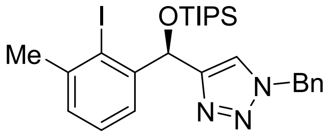

Following GP3, **9b** (1.70 g, 4.20 mmol) was dissolved in dry DCM (28.0 ml) with 2,6-lutidine (0.98 ml, 8.39 mmol) and trialkylsilyl trifluoromethanesulfonate (1.35 ml, 5.03 mmol) was stirring for 16 hours to isolated **6b** (1.93 g, 3.44 mmol, **82%**) as a white solid. Mp: 63 ^{0}C. ^{1}H NMR (600 MHz, Chloroform-d) δ 7.60 (d, J = 8.8 Hz, 1H), 7.36 – 7.31 (m, 3H), 7.25 (t, J = 7.5 Hz, 1H), 7.17 (d, J = 7.2 Hz, 4H), 6.40 (s, 1H), 5.45 (dd, J = 15.1, 15.0

Hz, 1H), 2.44 (s, 3H), 1.15 (hept, J = 7.4 Hz, 3H), 1.00 – 0.94 (m, 18H). ^{13}C NMR (150 MHz, Chloroform-d) δ 151.6, 146.2, 141.7, 135.0, 129.0, 128.5, 128.0, 127.7, 125.3, 121.7, 104.5, 73.9, 53.9, 29.7, 17.9, 12.2. A signal is missing due to overlap. HR-MS (EI, 70 eV): calculated for $C_{26}H_{36}IN_3OSi$ [M+H]$^+$: m/z = 562.16717, found: 562.16452 (Dev.: 0.02 mu; 0.09 ppm). IR (ATR): $\tilde{\nu}$ (cm^{-1})=3140, 2951, 2878, 1726, 1475, 1383, 1102, 896, 834, 702 cm^{-1}. HPLC (OM column, hexane-i-PrOH=98:2 as eluent, 1.0 mLmin-1): t=13.7 min, for >99.99% ee.

2.3.3. Synthesis of (*R*)-1-benzyl-4-((2-iodo-3 methoxyphenyl) ((triisopropylsilyl) oxy)methyl)-1*H*-1,2,3-triazole (6c)

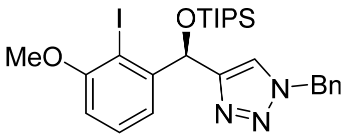

Following GP3, **9c** (1.75 g, 4.15 mmol) was dissolved in dry DCM (27.7 ml) with 2,6-lutidine (0.97 ml, 8.31 mmol) and trialkylsilyl trifluoromethanesulfonate (1.34 ml, 5.00 mmol) was stirring for 16 hours to isolated **6c** (2.09 g, 3.62 mmol, **87%**) as a white solid. Mp: 65 ^{0}C. ^{1}H NMR (600 MHz, Chloroform-d) δ 7.43 (dd, J = 7.8, 1.4 Hz, 1H), 7.36 – 7.29 (m, 4H), 7.19 (s, 1H), 7.16 (dd, J = 7.4, 2.0 Hz, 2H), 6.73 (dd, J = 8.1, 1.4 Hz, 1H), 6.39 (s, 1H), 5.53 – 5.39 (m, 2H), 3.87 (s, 3H), 1.15 (hept, J = 7.5 Hz, 3H), 0.97 (t, J = 7.3 Hz, 18H). ^{13}C NMR (150 MHz, Chloroform-d) δ 157.4, 151.4, 147.5, 135.0, 129.2, 129.0, 128.5, 127.7, 121.6, 120.6, 109.8, 89.7, 73.5, 56.5, 53.9, 17.9, 12.2. HR-MS (EI, 70 eV): calculated for $C_{26}H_{36}IN_3O_2Si$ [M]$^{+\cdot}$: m/z = 577.16207, found: 577.16930 (Dev.: 0.12 mu; 0.21 ppm). IR (ATR): $\tilde{\nu}$ (cm^{-1})=3122, 2930, 2853, 1702, 1443, 1350, 1078, 869, 804, 676 cm^{-1}. HPLC (OM column, hexane-i-PrOH=98:2 as eluent, 1.0 mLmin-1): t=28.0 min, for >99.99% ee.

3. Evaluation the Catalysts in Enantioselective Reactions:

3.1. Enantioselective Spirocyclizations

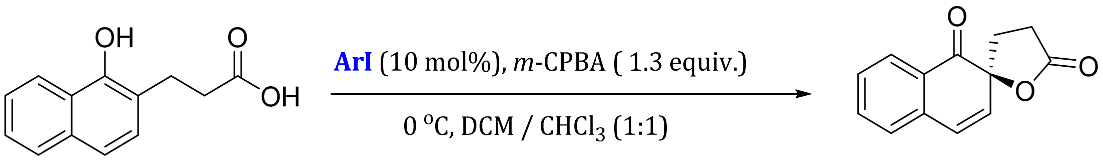

10 **11**

Catalyst	Time	Yield %	*ee* %
5	37 h	63	92
6a	28 h	70	83
6b	32 h	75	95
6c	24 h	85	97
**6c**[a]	**32 h**	**85**	**99**

a: This reaction occurred under – 10 °C and adding 6 equiv of EtOH

3.1.1. Typical Procedure of Enantioselective Kita-Spirocyclization

The naphthol **10** (20 mg, 9 µmmol, 1 equiv.) was dissolved in a mixture of DCM: $CHCl_3$ (1:1) (4.6 ml, 0.02 M) and cooled to 0 °C. Then the chiral iodoarene **6c** (10 mol %) and m-CPBA (75%, 28 mg, 0.12 mmol, 1.3 equiv.) were added consecutively. The reaction mixture was stirred at 0°C until full conversion of the starting material (indicated by TLC). Saturated $Na_2S_2O_3$ and 1 M Na_2CO_3 were added and the phases were separated. The aqueous layer was extracted with EtOAc (two times) and the combined organic layers were washed with brine and dried over Na_2SO_4. The solvent was removed under reduced pressure and the crude product was purified by column chromatography on silica gel using cyclohexane and EtOAc (5:1 to 2:1) to afford compound **11** as a white solid**.** Mp: 103-105 ^{0}C. ^{1}H NMR (600 MHz, Chloroform-d) δ 8.04 (d, *J* = 7.7 Hz, 1H), 7.65 (td, *J* = 7.5, 1.3 Hz, 1H), 7.43 (td, *J* = 7.6, 1.1 Hz, 1H), 7.29 (d, *J* = 1.8 Hz, 1H), 6.68 (d, *J* = 9.9 Hz, 1H), 6.23 (d, *J* = 9.9 Hz, 1H), 2.93 (ddd, *J* = 17.6, 11.3, 9.6 Hz, 1H), 2.62 (ddd, *J* = 17.6, 9.6, 2.1 Hz, 1H), 2.45 (ddd, *J* = 13.5, 9.6, 2.1 Hz, 1H), 2.21 (ddd, *J* = 13.5, 11.3, 9.7 Hz, 1H). ^{13}C NMR (150 MHz, Chloroform-d) δ 196.6, 176.5, 136.8, 135.7, 132.3, 129.0, 128.0, 128.0, 127.8, 127.4, 83.4, 31.3, 26.6. IR (ATR): $\tilde{\nu}$ (cm^{-1})= 1785, 1693, 1603, 1487, 1458, 1326, 1296, 1180, 1123, 1032, 930, 794, 702 cm^{-1}. MS (ESI): *m/z* = 237.0 [M+Na]$^+$. The analytical data are in accordance with the literature. [4]

The enantiomeric excess was determined by HPLC analysis on the purified product: Chiracel AM column, 80:20 hexane/*i*-PrOH, 1.0 mL/min, t_R = 5.75 min (S), t_R = 9.9 min (R).

Racemic Product

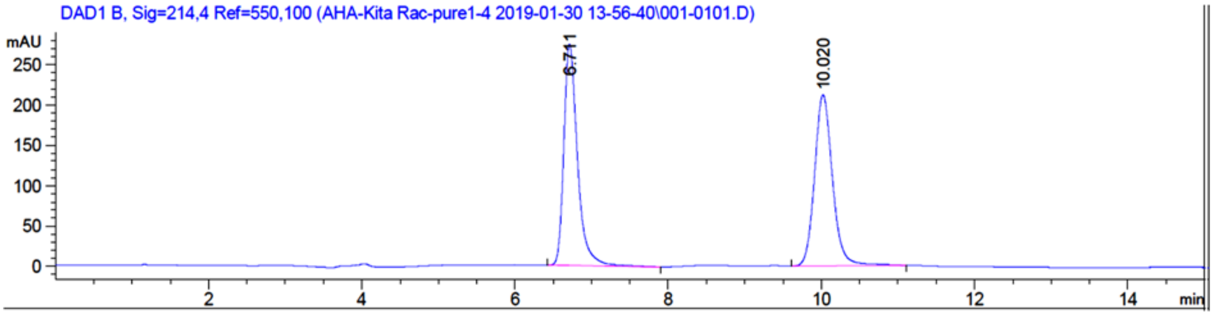

Peak #	RetTime [min]	Type	Width [min]	Area [mAU*s]	Height [mAU]	Area %
1	6.711	BB	0.1807	3404.25635	274.50613	49.6873
2	10.020	BB	0.2414	3447.09961	211.77893	50.3127

Enantioselective Product:

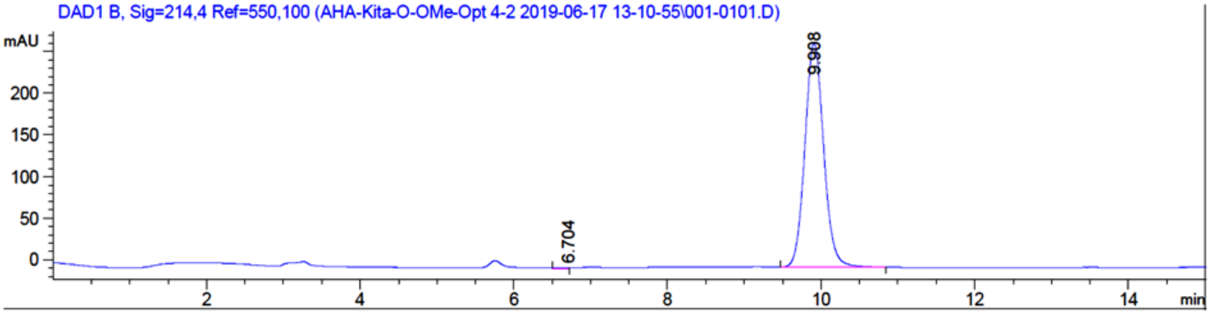

Peak #	RetTime [min]	Type	Width [min]	Area [mAU*s]	Height [mAU]	Area %
1	6.704	MM R	0.2100	19.03521	1.51096	0.4153
2	9.908	BB	0.2598	4564.32422	269.26074	99.5847

Mixture of enantiomer product with the racemic compound:

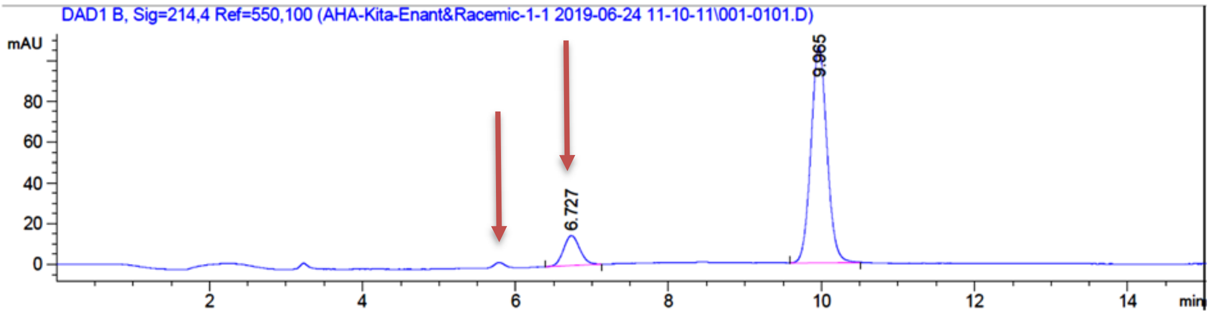

Peak #	RetTime [min]	Type	Width [min]	Area [mAU*s]	Height [mAU]	Area %
1	6.727	BB	0.1809	217.70120	14.77281	12.3543
2	9.965	BB	0.2203	1544.44861	106.72050	87.6457

3.1.2. Synthesis and screening the reactivity of triazolium salt (6d) in Enantioselective Kita Spirocyclizations

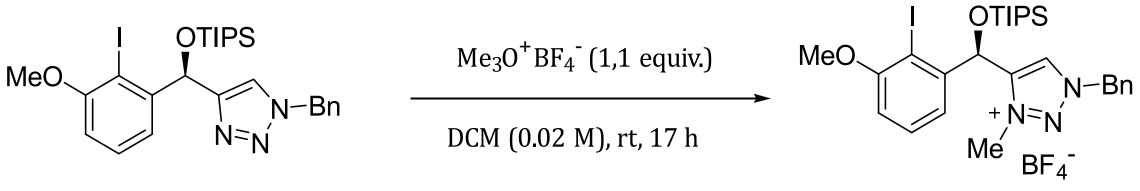

6c **6d**

To a solution of **6c** (40 mg, 6.9 μmol) in 3.5 mL dry DCM at room temperature was added trimethyloxonium tetrafluoroborate (11 mg, 0.076 mmol). The mixture was stirred at room temperature for 17 h, then the solvent was removed under vacuum to afford of **6d** (39.5 mg, 0.07 mmol, 96%) as off-white solid. Mp: 78 ^{0}C. ^{1}H NMR (600 MHz, Chloroform-d) δ 8.83 (s, 1H), 7.47 – 7.50 (m, 3H), 7.38 – 7.42 (m, 5H), 6.84 (d, J = 9.4 Hz, 1H), 6.47 (s, 1H), 5.79 (d, J = 4.8 Hz, 2H), 4.12 (s, 3H), 3.87 (s, 3H), 1.09 – 1.18 (m, 4H), 0.88– 0.97 (m, 17H). ^{13}C NMR (150 MHz, Chloroform-d) δ 158.3, 144.5, 140.6, 130.4, 130.25, 129.9, 129.5, 129.4, 121.3, 111.8, 90.2, 72.0, 57.8, 56.7, 55.5, 17.8, 12.1. HR-MS (EI, 70 eV): calculated for $C_{27}H_{39}IN_3O_2Si$ $[M]^{+\cdot}$: m/z = 592.18502, found: 592.18943 (Dev.: 0.04 mu; 0.12 ppm). IR (ATR): $\tilde{\nu}$ (cm-1)=3240, 2983, 2875, 1734, 1465, 1377, 1090, 898, 820, 703 682 cm^{-1}. HPLC (OM column, hexane-i-PrOH=98:2 as eluent, 1.0 mLmin-1): t=24.0 min, for >99.99% ee.

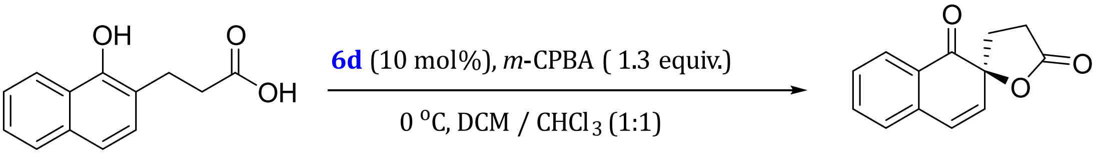

10 **11** **32% Yield 61 ee%**

The catalyst **6d** provided low reactivity and stereoselectivity in Kita Spirolactonization due to the blocking of N-I bound intermediate which increase the reactivity of the catalyst.

3.2. Enantioselective 4-Hydroxylation of Phenols

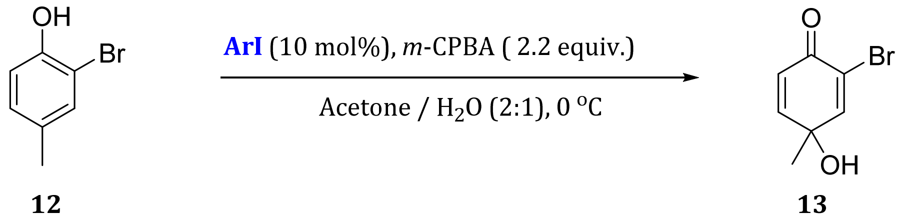

Catalyst	Time	Yield %	*ee* %
5	26 h	65	76
6a	20 h	72	63
6b	20 h	70	88
**6c**	**16 h**	**91**	**93**

3.2.1. Optimization of the Reaction Conditions

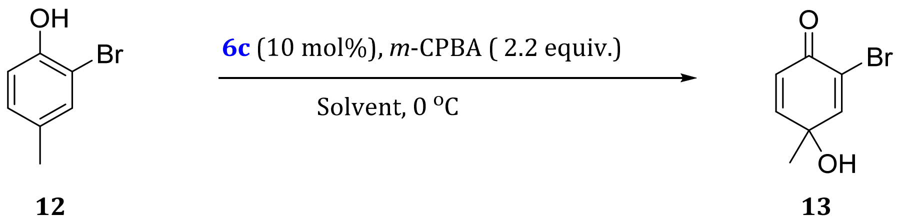

Solvent	Time	Yield %	*ee* %
**Acetone/H_2O[a]**	**16 h**	**92**	**93**
MeCN/H_2O[b]	25 h	70	78
DMF/H_2O[b]	36 h	40	55
Butanone/H_2O[a]	20 h	78	83

a: Acetone or Butanone: H_2O (2:1), b: DMF or MeCN:H_2O (9:1)

3.2.2. Typical Procedure of 4-Hydroxylation of Phenols

To a solution of compound **12** (50 mg, 0.27 mmol, 1 equiv.) in mixture of Acetone (110 μL) and water (55 μL) at 0 °C was added chiral aryl iodide **6c** (10 mol %) and m-CPBA (75%, 101 mg, 0.59 mmol, 2.2 equiv.) and the mixture was stirred at 0 °C. Progress of the reaction was monitored by TLC analysis. The reaction was quenched with saturated $Na_2S_2O_3$ aq. (2 mL) and stirred for 5 min at 0 °C, followed by the addition of saturated $NaHCO_3$ aq. (2 mL). The organic layer was extracted with EtOAc, washed with brine, dried over anhydrous Na_2SO_4, and evaporated under reduced pressure. The residue was purified by column chromatography. The residue was purified by column chromatography to provide **13** as a pale orange solid. Mp: 103-105 ^{0}C. ^{1}H NMR (600 MHz, Chloroform-d) δ 7.33 (d, J = 2.8 Hz, 1H), 6.92 (dd, J = 9.9, 2.8 Hz, 1H), 6.25 (d, J = 9.9 Hz, 1H), 2.2 (s, 1H), 1.53 (s, 3H). ^{13}C NMR (150 MHz, Chloroform-d) δ 178.1, 152.2, 151.9, 125.7, 123.6, 70.0, 26.5. HR-MS (EI, 70 eV)= calculated for $C_7H_7BrO_2Na$ $[M]^+$ $[M]^{+\cdot}$: m/z = 224.95218, found 224.95224 (Dev.: 0.06 mu; 0.14 ppm). IR (ATR): $\tilde{\nu}$ (cm^{-1})=3474, 3045, 2980, 2937, 1660, 1594, 1052 cm^{-1}.

The enantiomeric excess was determined by HPLC analysis on the purified product: Chiracel OM column, 95:5 hexane/*i*-PrOH, 1.0 mL/min, t_R = 14.82 min (major), t_R = 17.1 min (minor). Due to missing comparable literature samples the absolute configuration was not determined yet.

Racemic Product:

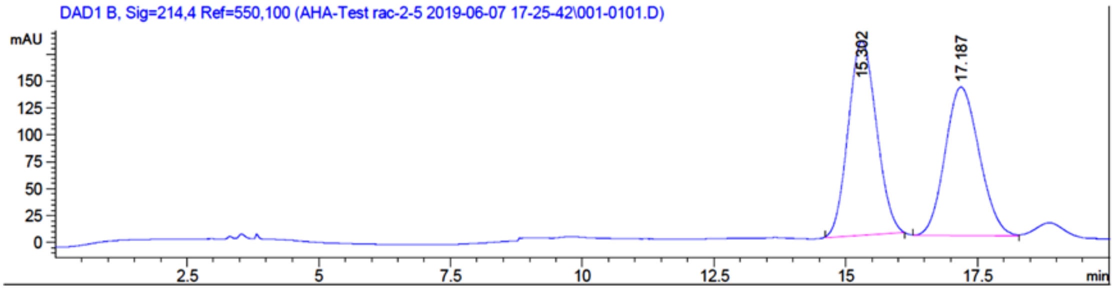

Peak #	RetTime [min]	Type	Width [min]	Area [mAU*s]	Height [mAU]	Area %
1	15.302	MM R	0.6210	6727.67969	180.57063	51.8562
2	17.187	MM R	0.8005	6246.05566	138.04660	48.1438

Enantioselective Product:

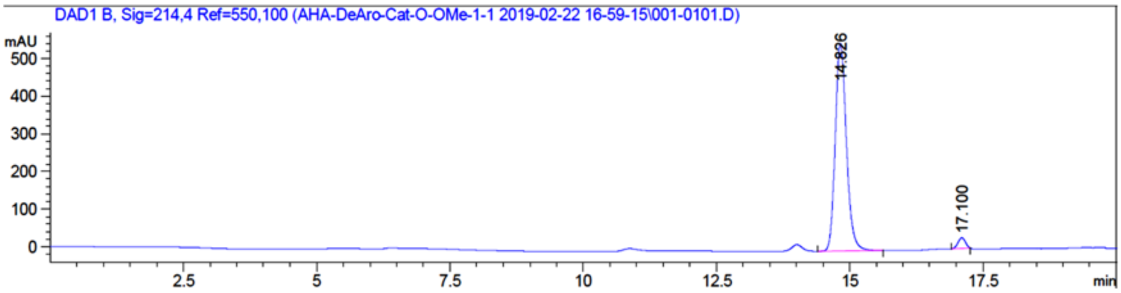

Peak #	RetTime [min]	Type	Width [min]	Area [mAU*s]	Height [mAU]	Area %
1	14.826	BB	0.2220	8031.82568	551.10516	96.6835
2	17.100	MM R	0.1614	275.51602	28.45077	3.3165

3.3. Enantioselective α-Oxidation of Ketones

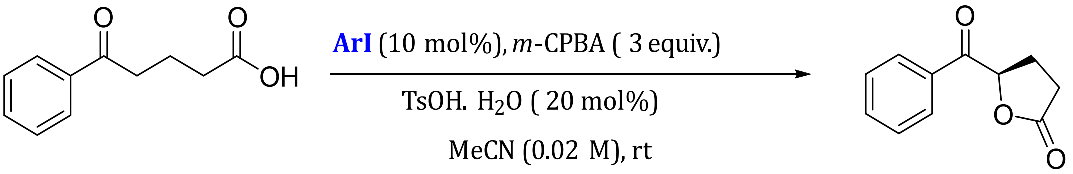

14 **15**

Catalyst	Time	Yield %	*ee* %
5	32 h	61	40
6a	24 h	65	36
6b	28 h	69	56
**6c**	**18 h**	**83**	**68**

3.3.1. Optimization of the Reaction Conditions

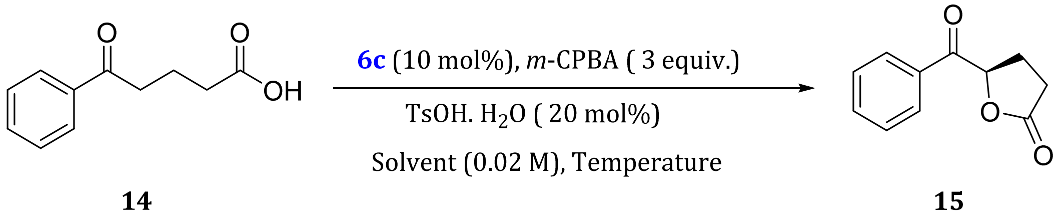

Solvent	Temperature	Time	Yield %	*ee* %
MeCN	rt	18	83	68
DCM	rt	18	70	58
$CHCl_3$	rt	18	85	74
EtOAc	rt	18	74	62
$CHCl_3$	0 °C	30	80	82
**$CHCl_3$/MeOH [a]**	**0 °C**	**20**	**88**	**81**
$CHCl_3$/H_2O [b]	0 °C	24	75	77
Toluene	0 °C	27	60	25
Toluene/H_2O [b]	0 °C	22	72	50
$CHCl_3$/MeOH	-20 °C	72	65	86

a: MeOH (10 Equiv), b: Toluene or $CHCl_3$:H_2O (5:1)

3.3.2. Typical Procedure of α- Oxidation of Ketones

5-Oxo-5-phenylpentanoic acid **14** (20 mg, 0.1 mmol, 1 equiv) was added to a solution of chiral aryl iodide **6c** (10 mol %), m-CPBA (75%, 0.31 mmol, 3 equiv.) and *p*-TsOH hydrate (4 mg, 0.02 mmol, 0.2 equiv.) in (0,4 ml) of a mixture of $CHCl_3$ and 10 equiv of MeOH (0.4 mL) at 0 °C, and the solution was stirred until precipitation occurred. The reaction was quenched by addition of sat. aq. sodium thiosulfate (3 mL), extracted with $CHCl_3$ (3 × 5 mL), washed with sat. aq $NaHCO_3$ (5 mL), dried (Na_2SO_4), filtered and concentrated. The residue was purified by flash chromatography using cyclohexane and EtOAc (9:1) to provide **15** as a white solid. Mp: 93- 95 ^{0}C. ^{1}H NMR (600 MHz,

Chloroform-d) δ 7.98 (d, J = 7.5 Hz, 2H), 7.65 (t, J = 7.4 Hz, 1H), 7.57 – 7.45 (m, 2H), 5.85 – 5.78 (m, 1H), 2.65 – 2.43 (m, 4H). ^{13}C NMR (150 MHz, Chloroform-d) δ 194.3, 176.3, 133.6, 133.2, 129.0, 128.8, 78.3, 26.8, 25.0. IR (ATR): $\tilde{\nu}$ (cm^{-1})= 2982, 1762, 1680, 1461, 1365, 1224 cm^{-1}. MS (EI, m/z): Calculated for [$C_{11}H_{10}O_3$] $(M+NH_4)^+$ 208.1 found 208.4. The analytical data are in accordance with the literature.[5]

The enantiomeric excess was determined by HPLC analysis on the purified product: Chiracel OM column, 80:20 hexane/*i*-PrOH, 1.0 mL/min, t_R = 10.04 min (R), t_R = 12.7 min (S).

Racemic Product:

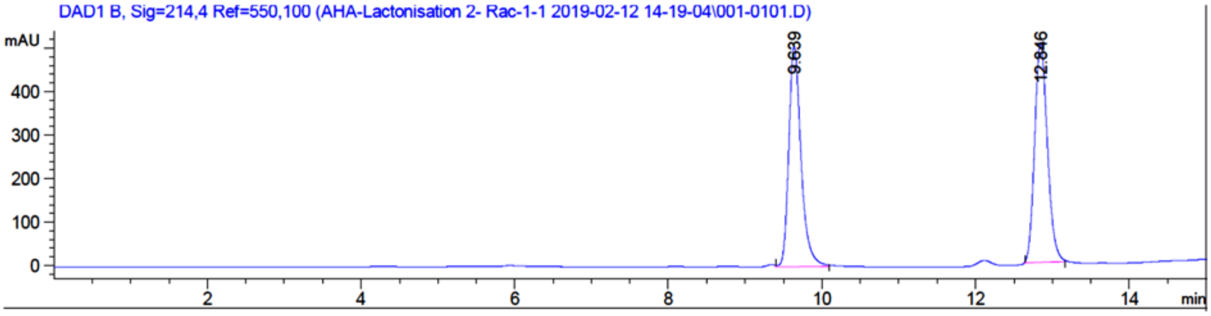

Peak #	RetTime [min]	Type	Width [min]	Area [mAU*s]	Height [mAU]	Area %
1	9.639	MM R	0.2191	5630.65967	506.68408	49.2589
2	12.846	MM R	0.1914	5800.09521	505.19043	50.7411

Enantioselective Product:

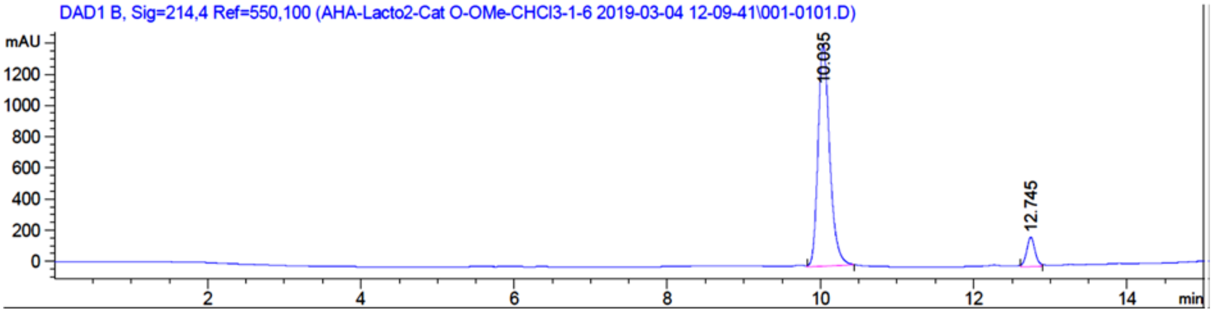

Peak #	RetTime [min]	Type	Width [min]	Area [mAU*s]	Height [mAU]	Area %
1	10.035	MM R	0.1756	1.50466e4	1428.17053	91.1665
2	12.745	MM R	0.1282	1457.93958	189.61247	8.8335

3.4. α-Tosyloxylation of Propiophenone

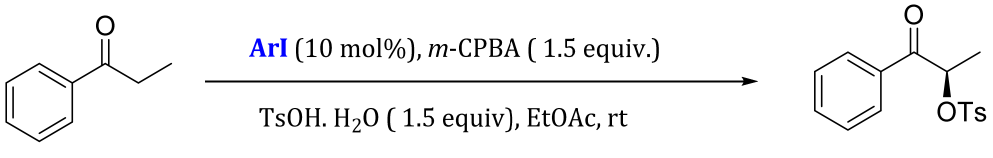

16 17

Catalyst	Time	Yield %	*ee* %
5	44 h	68	57
6a	33 h	72	55
6b	40 h	76	66
**6c**	**30 h**	**84**	**80**

3.4.1. Optimization of the Reaction Conditions

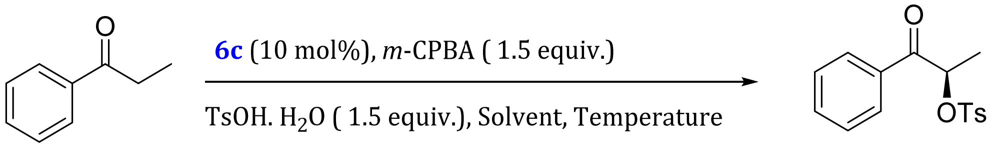

16 17

Solvent	Temperature	Time	Yield %	*ee* %
DCM	rt	36	72	68
MeCN	rt	48	78	65
EtOAc	rt	30	84	80
EtOAc/$CHCl_3$[a]	rt	36	73	76
**EtOAc**	**0 °C**	**48**	**90**	**88**
EtOAc	-20 °C	72	64	90

a: Mixture of EtOAc: $CHCl_3$ (1:1)

3.4.2. Typical Procedure of α-Tosyloxylation of Propiophenone

Catalyst **6c** (10 mol %) was dissolved in $CHCl_3$ (0.38 mL) with m-CPBA (75%, 51 mg, 0.22 mmol, 1.5 equiv) and *p*-TsOH hydrate (43 mg, 0.22 mmol, 1.5 equiv). The reaction mixture was stirred for one hour then the solvent was removed in vacuo. A solution of propiophenone **16** (20 µl, 0.15 mmol, 1 equiv.) in EtOAc (0.38 mL) was added and the reaction was stirred until disappearance of the starting material (monitored by TLC). The reaction mixture was washed with a saturated aqueous solution of $Na_2S_2O_3$, and the aqueous layer was extracted twice with EtOAc. The combined organic layers were washed with a saturated aqueous solution of $NaHCO_3$, then brine. The organic layer was dried over Na_2SO_4, and the solvent was evaporated under reduced pressure. The crude mixture was purified by column chromatography on silica gel with cyclohexane/ EtOAc (7:3) to provide 41 mg (90% yield and 88% ee) of **17** as a white solid. Mp: 68-69 ^{0}C. ^{1}H NMR (600 MHz, Chloroform-d) δ 7.88 (dd, *J* = 8.4, 1.2 Hz, 2H), 7.76 (d, *J* = 8.3 Hz, 2H), 7.62 – 7.57 (m, 1H), 7.46 (t, *J* = 7.8 Hz, 2H), 7.27 (d, *J* = 6.8 Hz, 2H), 5.79 (q, *J* = 7.0 Hz, 1H), 2.41 (s, 3H), 1.60 (d, *J* = 7.0 Hz, 3H). ^{13}C NMR (150 MHz, Chloroform-d) δ 194.9, 145.0, 133.9, 133.7, 133.5, 129.8, 128.9, 77.4, 21.7, 18.8. HR-MS (EI, 70 eV): calculated for $C_{16}H_{16}NaO_4S$ $[M+Na]^+$ 327.0663, found 327.0656. (Dev.: - 0.07 mu; -0.17 ppm). IR (ATR): $\tilde{\nu}$ (cm^{-1})=1170, 1370, 1700 cm^{-1}.

The enantiomeric excess was determined by HPLC analysis on the purified product: Chiracel AM column, 98:2 hexane/*i*-PrOH, 1.5 mL/min, t_R = 14.52 min (S), t_R = 16.9 min (R).

Racemic Product:

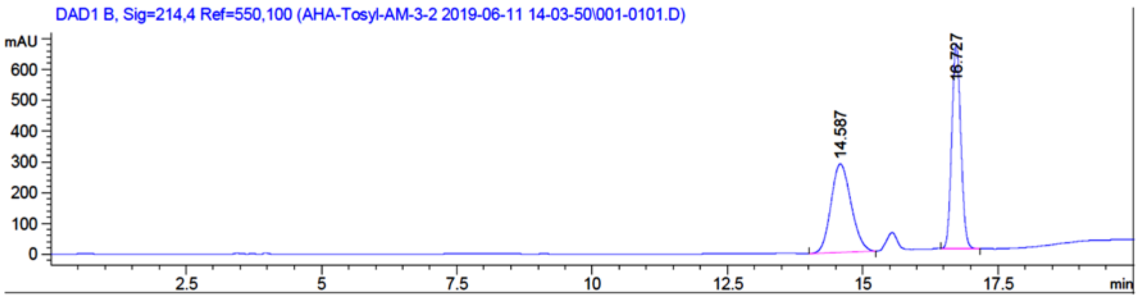

Peak #	RetTime [min]	Type	Width [min]	Area [mAU*s]	Height [mAU]	Area %
1	14.587	BB	0.3959	7449.69043	289.30524	49.0035
2	16.727	BB	0.1820	7752.66895	667.39752	50.9965

Enantioselective Product:

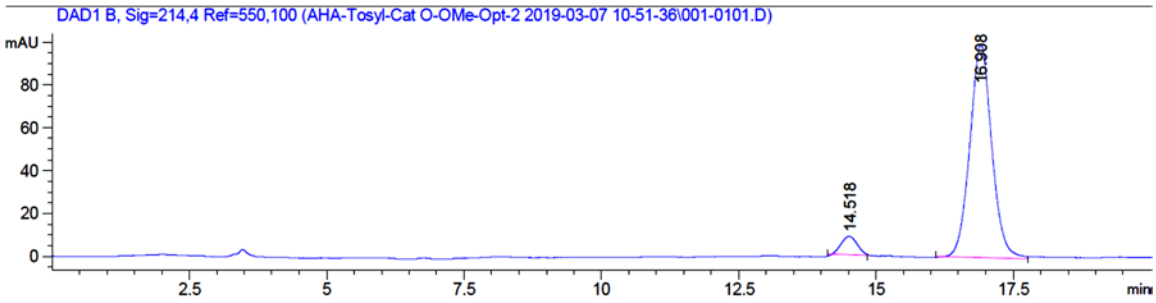

Peak #	RetTime [min]	Type	Width [min]	Area [mAU*s]	Height [mAU]	Area %
1	14.518	MM R	0.3340	171.09436	8.53758	5.8208
2	16.908	MM R	0.4633	2768.26880	99.57507	94.1792

3.4. **Enantioselective Oxidative Rearrangement of Allylic Alcohols**

3.4.1. Preparation of the allylic alcohol 19

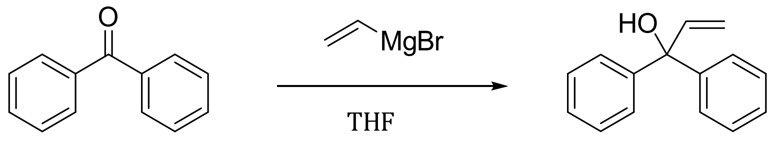

18 **19**

Under N_2, to a solution of Benzophenone **18** (11 mmol, 1.0 equiv.) in THF (13.2 ml) was added a solution of vinyl magnesium bromide (13.2 mL, 1 M in THF, 1.2 equiv.) at 0 °C over 15 min. The reaction mixture was stirred for 2 h at 0 °C, then stirred at room temperature for 6 h and quenched with saturated aqueous NH_4Cl. The organic layer was extracted with EtOAc, washed with brine, dried over anhydrous Na_2SO_4, and evaporated under reduced pressure. The crude material was purified by flash chromatography (Cyclohexane: EtOAc 95:5) to afford the allylic alcohol **19** (2.03 g, 9.65 mmol, 88%) as a colorless oil. ^{1}H NMR (600 MHz, Chloroform-d) δ 7.44 – 7.40 (m, 4H), 7.36 (t, J = 7.6 Hz, 4H), 7.30 (t, J = 7.3 Hz, 2H), 6.54 (dd, J = 16.8, 10.8 Hz, 1H), 5.38 – 5.35 (m, 1H), 5.34 (s, 1H), 2.38 (s, 1H). ^{13}C NMR (150 MHz, Chloroform-d) δ 145.8, 143.6, 128.2, 127.3, 127.0, 114.1, 79.5. HR-MS (EI, 70 eV): calculated for

($C_{15}H_{14}NaO$) m/z $[M+Na]^+$= 233.09421, found 233.09419 (Dev.: 0.02 mu; 0.06 ppm). IR (ATR): $\tilde{\nu}$ (cm^{-1})= 3462, 3067, 3031, 2923, 2857, 1591, 1494, 1458, 1340, 1163, 990, 928, 753, 704.

3.4.2. Typical Procedure of Enantioselective Oxidative Rearrangement of Allylic Alcohols.

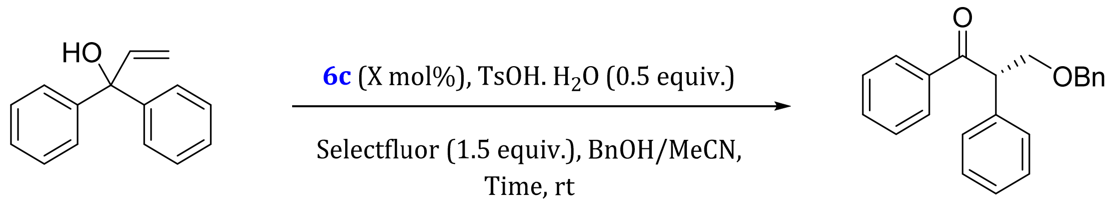

19 **20**

Catalyst Loading	Time	Yield %	*ee* %
20 mol %	8 h	82	95
15 mol %	12 h	81	94
10 mol %	16 h	84	95
**10 mol %[a]**	**24 h**	**80**	**98**

a: The reaction occur under 10 °C.

Under N_2, the dry RBF was charged with catalyst **6c** (0.05 mmol, 20 mol %), $TsOH.H_2O$ (20 mg, 0.12 mmol, 0.5 equiv.) and Selectfluor (130 mg, 0.36 mmol, 1.5 equiv), and benzyl alcohol (1.2 mL, 4.81 mmol). A solution of allylic alcohol **19** (50 mg, 0.24 mmol, 1.0 equiv.) in MeCN (1.2 mL) was added dropwise to the reaction mixture over 10 min at 10 °C. After that, the reaction mixture was stirred for 24 hours at the same temperature. The crude material was purified by flash chromatography with (Cyclohexane: EtOAc 97:3) to provide 100 mg (80% yield and 98% ee) of **20**. ^1H NMR (600 MHz, Chloroform-d) δ 7.98 (dd, J = 8.4, 1.3 Hz, 2H), 7.46 – 7.43 (m, 1H), 7.33 – 7.23 (m, 12H), 4.94 (dd, J = 8.6, 5.4 Hz, 1H), 4.59 (d, J = 12.2 Hz, 1H), 4.50 (d, J = 12.0 Hz, 1H), 4.32 – 4.26 (m, 1H), 3.74 (dd, J = 9.2, 5.4 Hz, 1H). ^{13}C NMR (150 MHz, Chloroform-d) δ 198.4, 138.3, 136.9, 136.5, 133.1, 129.1, 128.8, 128.6, 128.5, 128.4, 127.7, 127.6, 127.2, 73.5, 72.4, 54.1. IR (ATR): $\tilde{\nu}$ (cm^{-1})= 3074, 3023, 2915, 2852, 1674, 1612, 1483, 1245, 1204, 1080, 742, 689, 619, 538. MS (EI, m/z): Calculated for $[C_{21}H_{18}O_2]$: m/z = 302.2 $(M)^+$. The analytical data are in accordance with the literature.[6]

The enantiomeric excess was determined by HPLC analysis on the purified product: Chiracel OM column, 98:2 hexane/*i*-PrOH, 1.5 mL/min, t_R = 8.6 min (minor), t_R = 9.8 min (major). Due to missing comparable literature samples the absolute configuration of the major isomer was not determined yet.

Racemic Product:

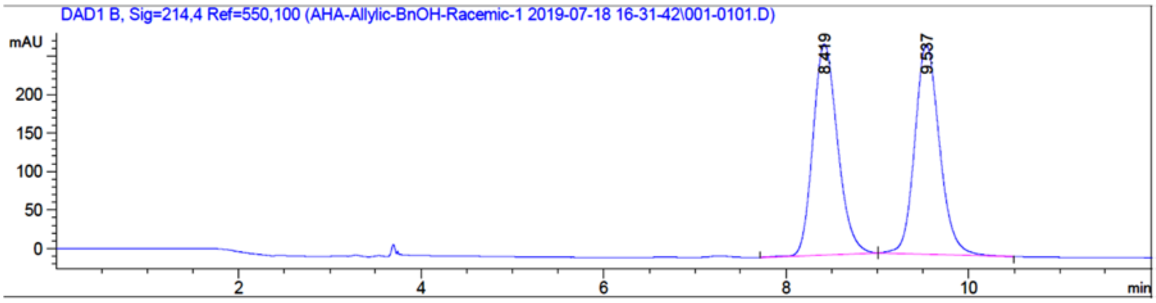

Peak #	RetTime [min]	Type	Width [min]	Area [mAU*s]	Height [mAU]	Area %
1	8.419	BB	0.2891	5136.49609	274.11166	50.0394
2	9.537	BB	0.2894	5128.41309	272.08228	49.9606

Enantioselective Product:

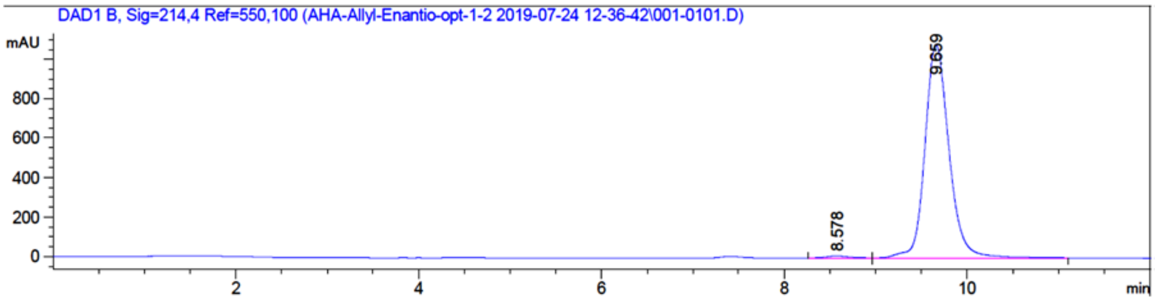

Peak #	RetTime [min]	Type	Width [min]	Area [mAU*s]	Height [mAU]	Area %
1	8.578	BB	0.1981	156.54196	9.38473	0.7859
2	9.659	BB	0.2786	1.97614e4	1081.88391	99.2141

4. NMR Spectra for New Compounds

4.1. NMR of (*S*)-1-(3-chloro-2-iodophenyl)prop-2-yn-1-ol (8a) in $CDCl_3$

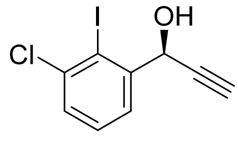

8a

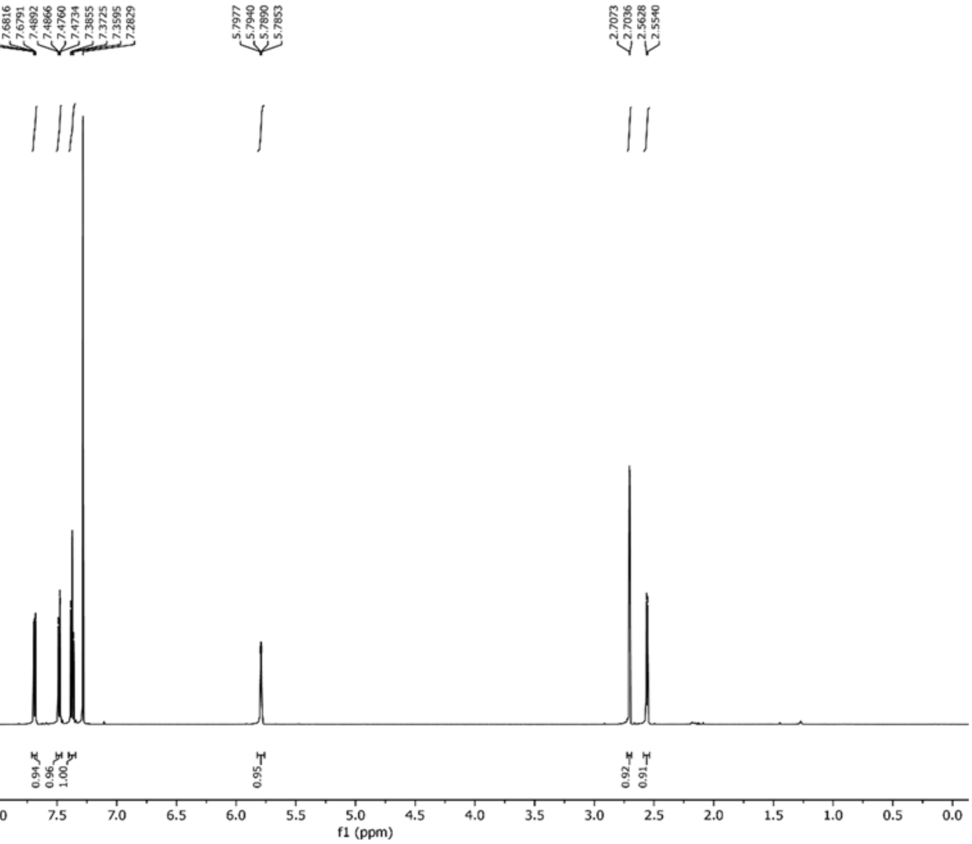

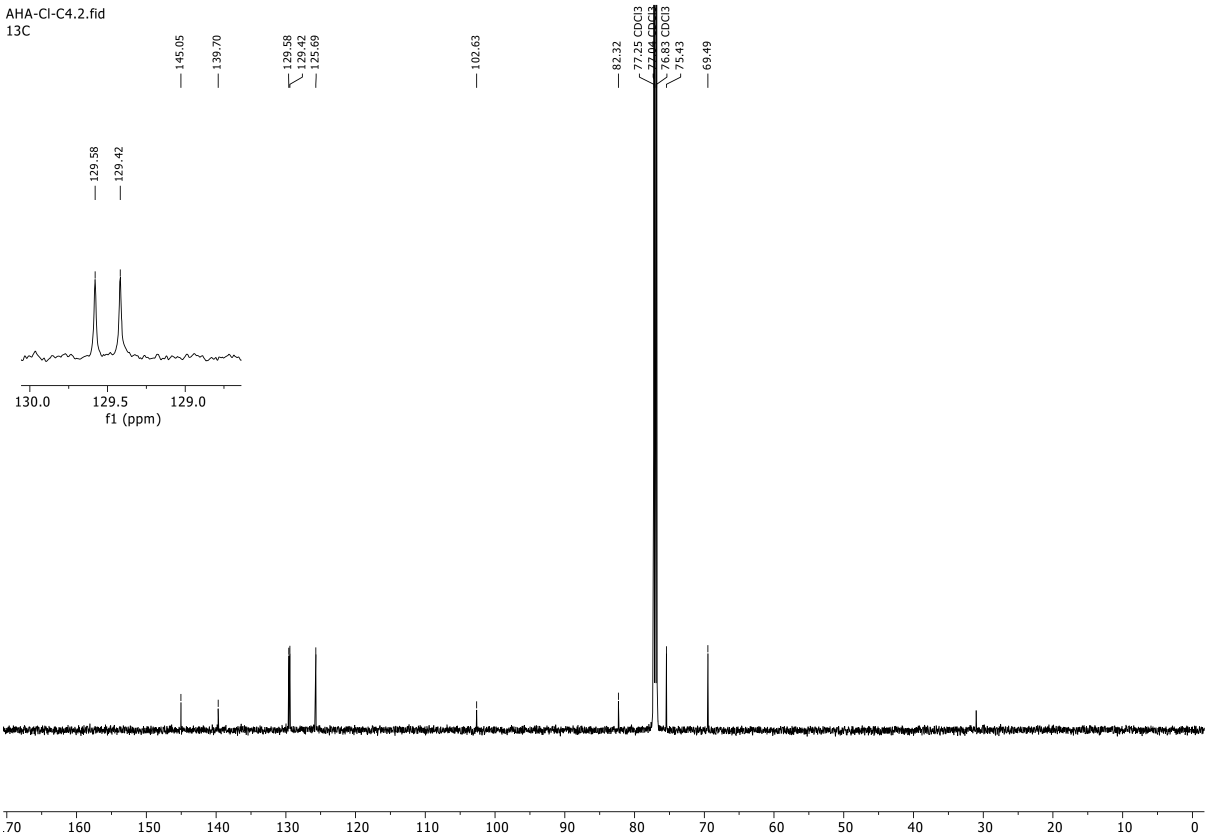

4.2. NMR of (*S*)-1-(2-iodo-3-methylphenyl)prop-2-yn-1-ol (8b) in $CDCl_3$

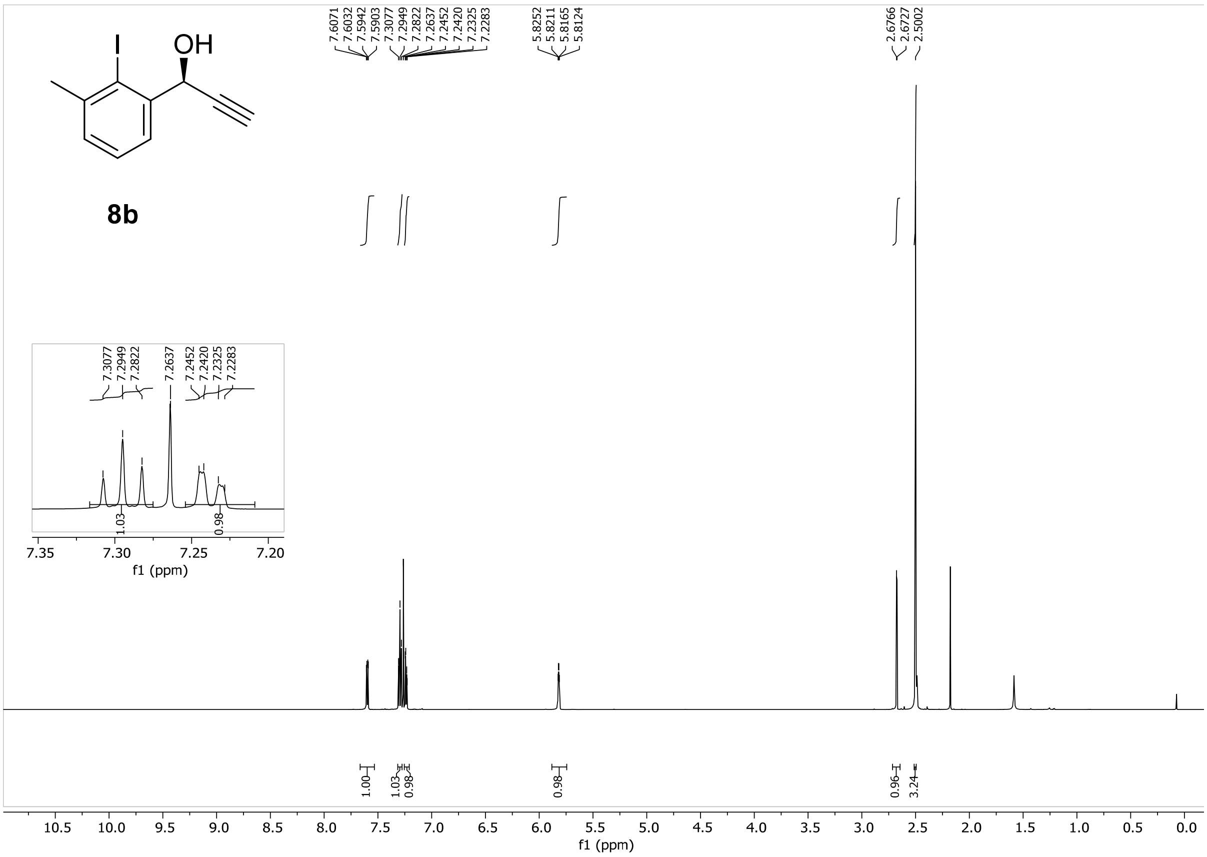

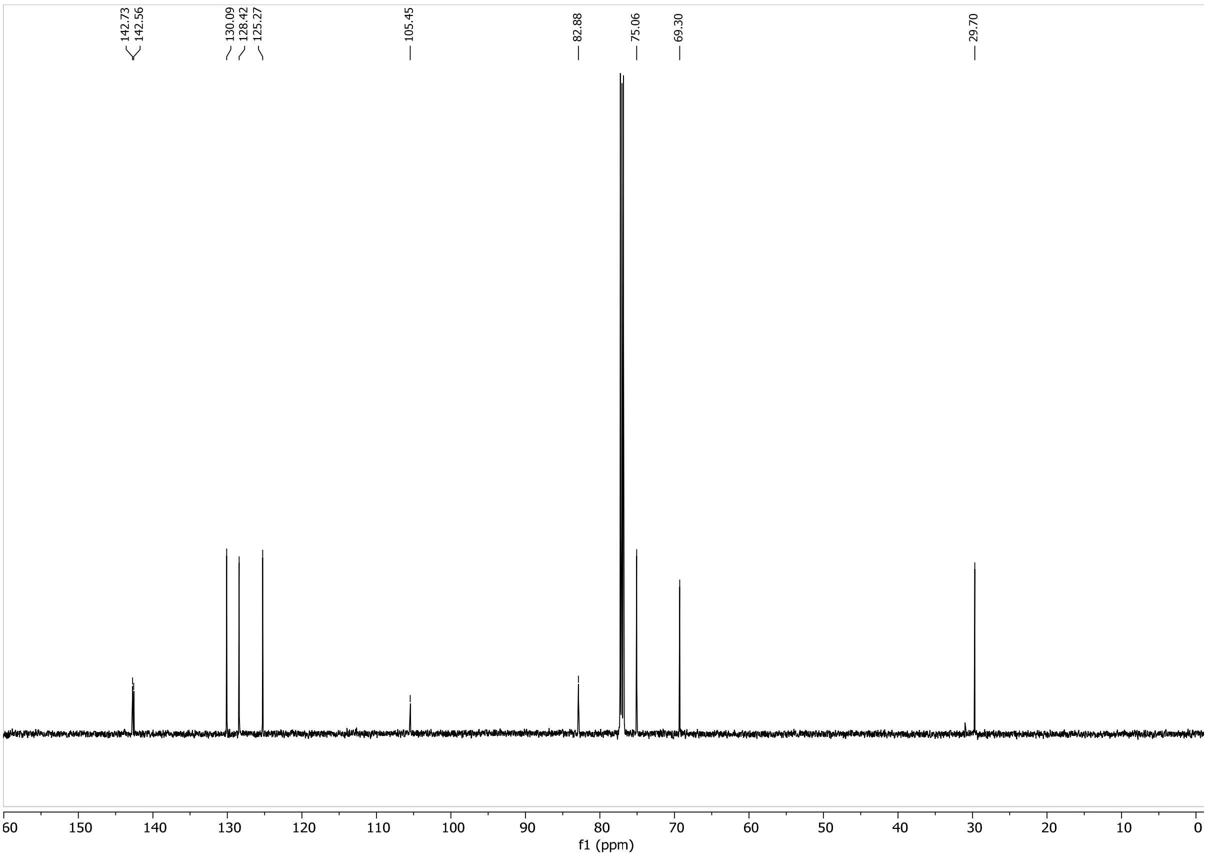

4.3. NMR of (*S*)-1-(2-iodo-3-methoxyphenyl)prop-2-yn-1-ol (8c) in $CDCl_3$

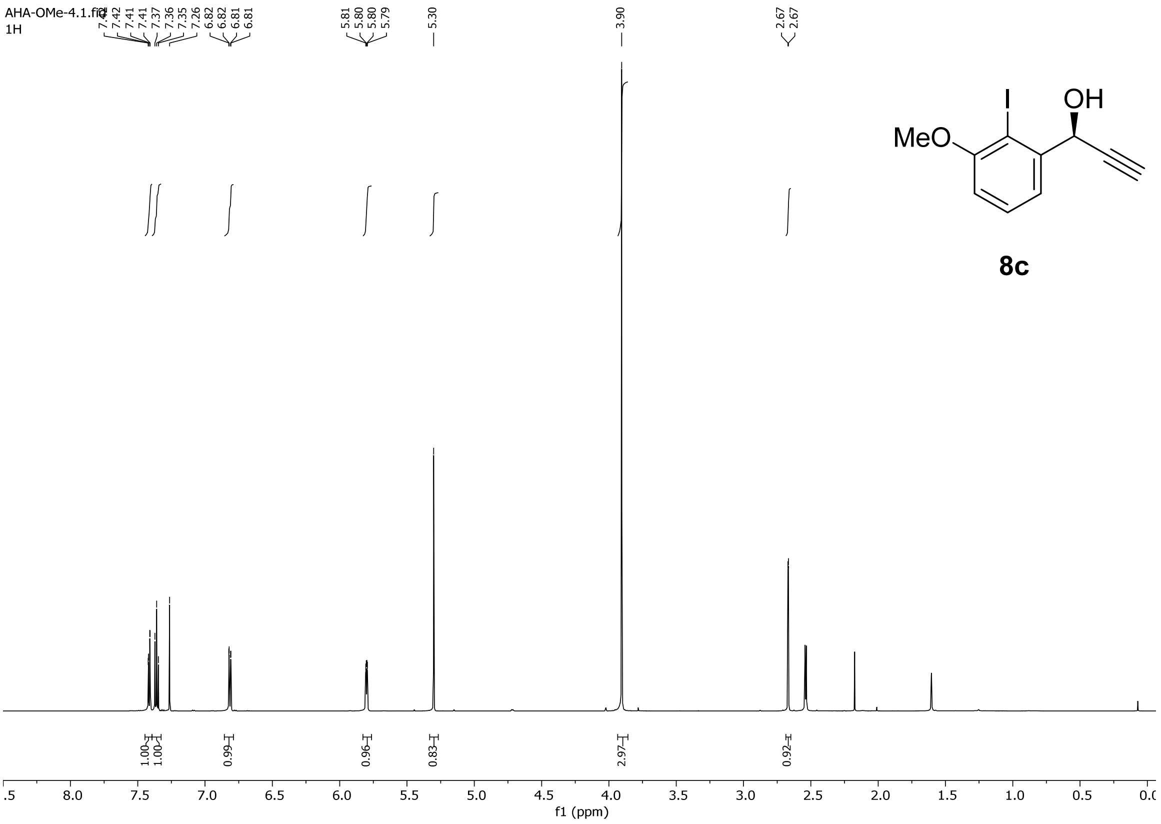

8c

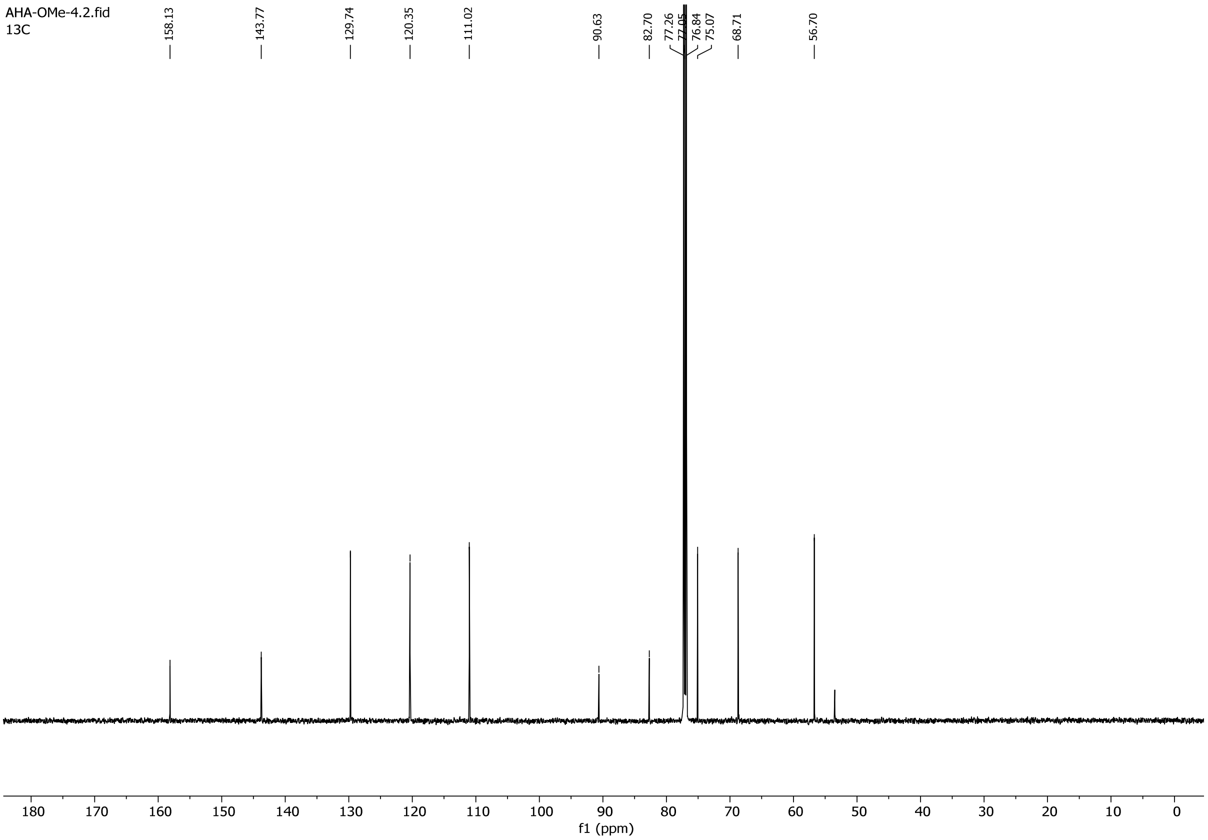

4.4. NMR of of (*R*)-(1-benzyl-1*H*-1,2,3-triazol-4-yl)(3-chloro-2- iodophenyl) methanol (9a) in $CDCl_3$

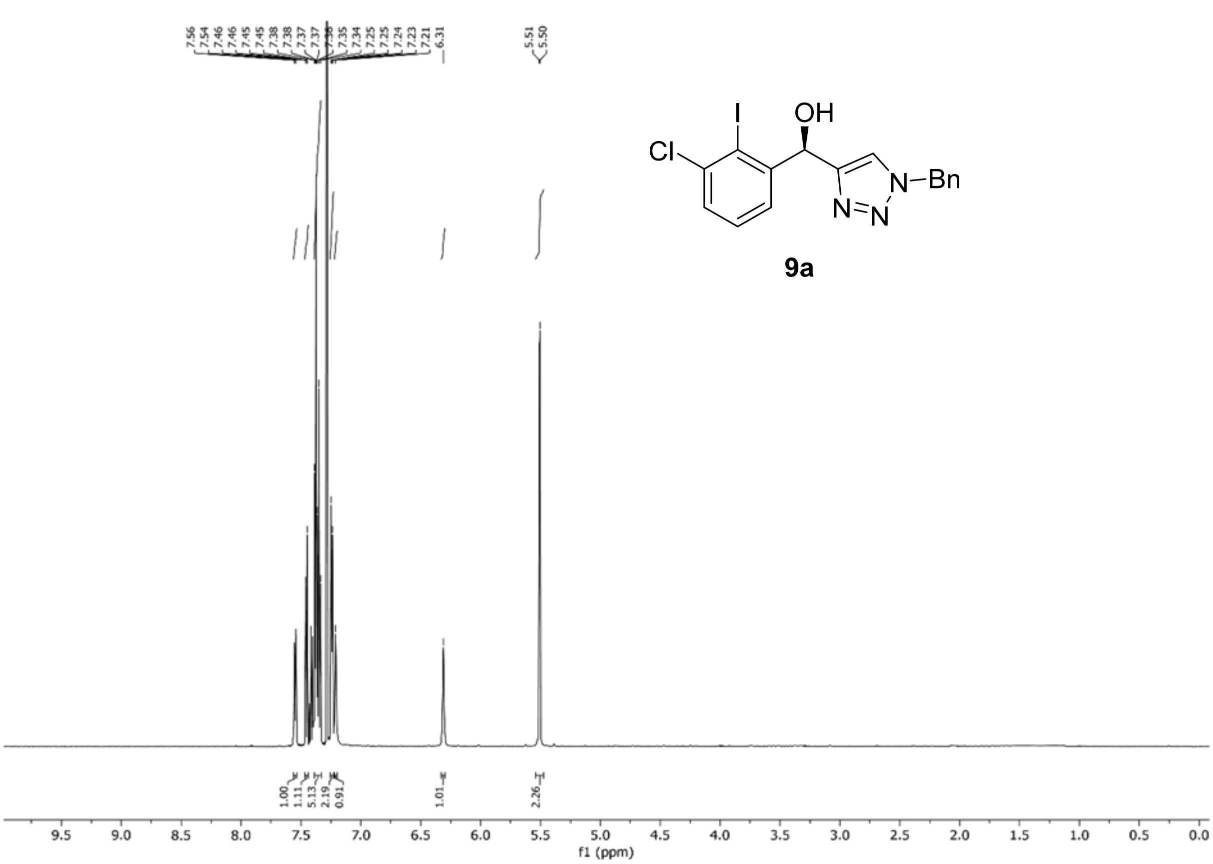

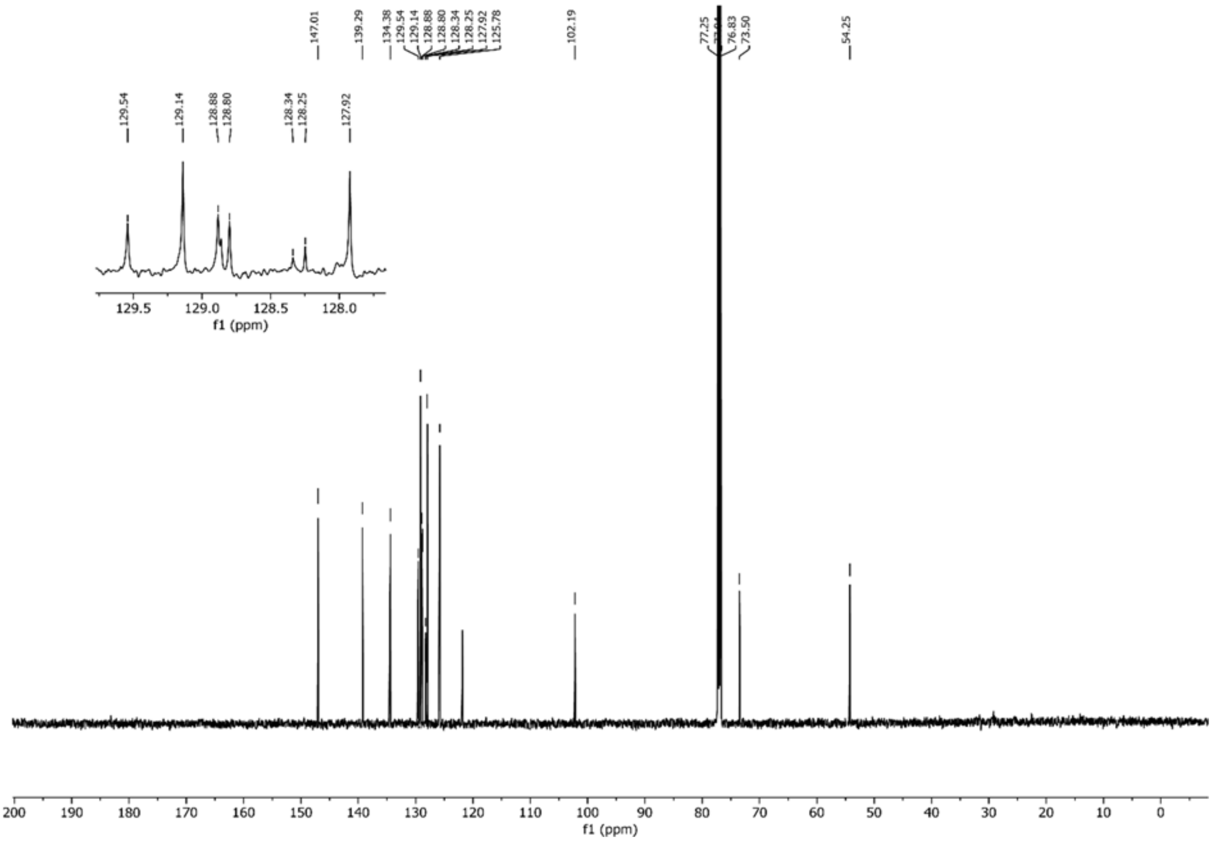

4.5. NMR of (*R*)-(1-benzyl-1*H*-1,2,3-triazol-4-yl)(2-iodo-3-methyl phenyl) methanol (9b) in $CDCl_3$

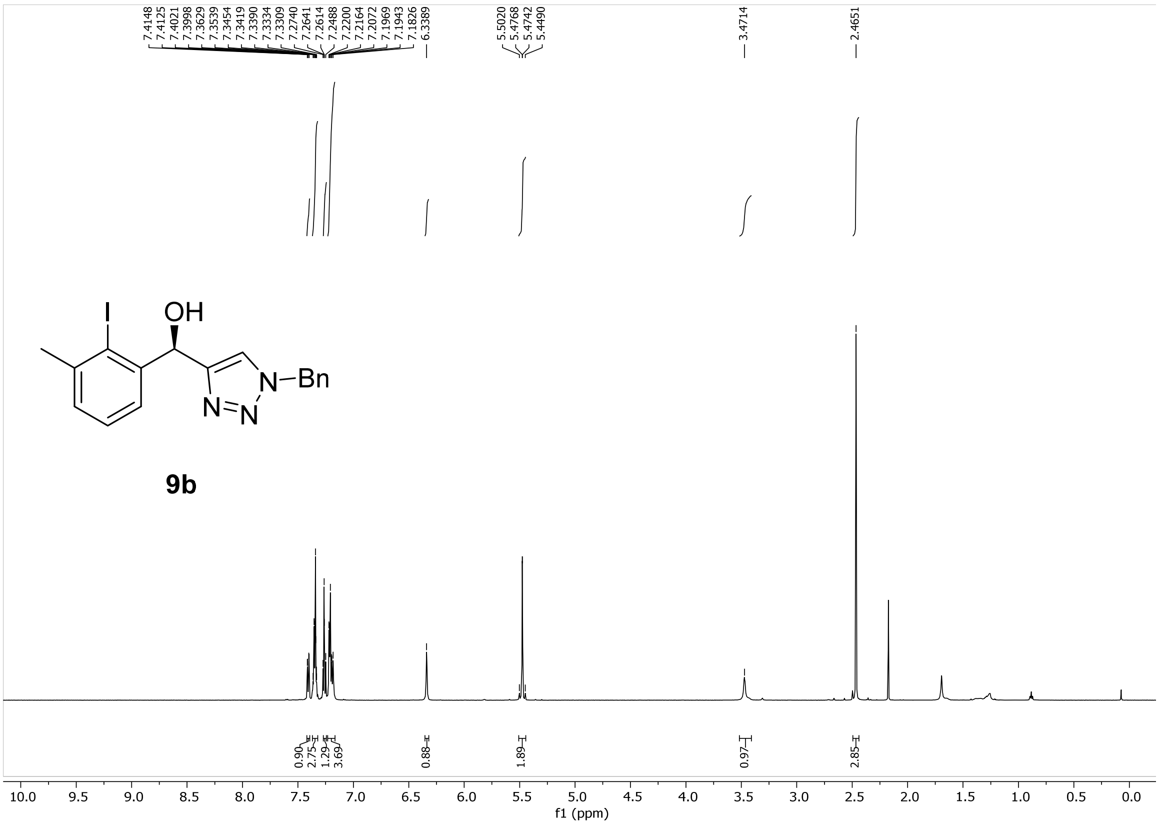

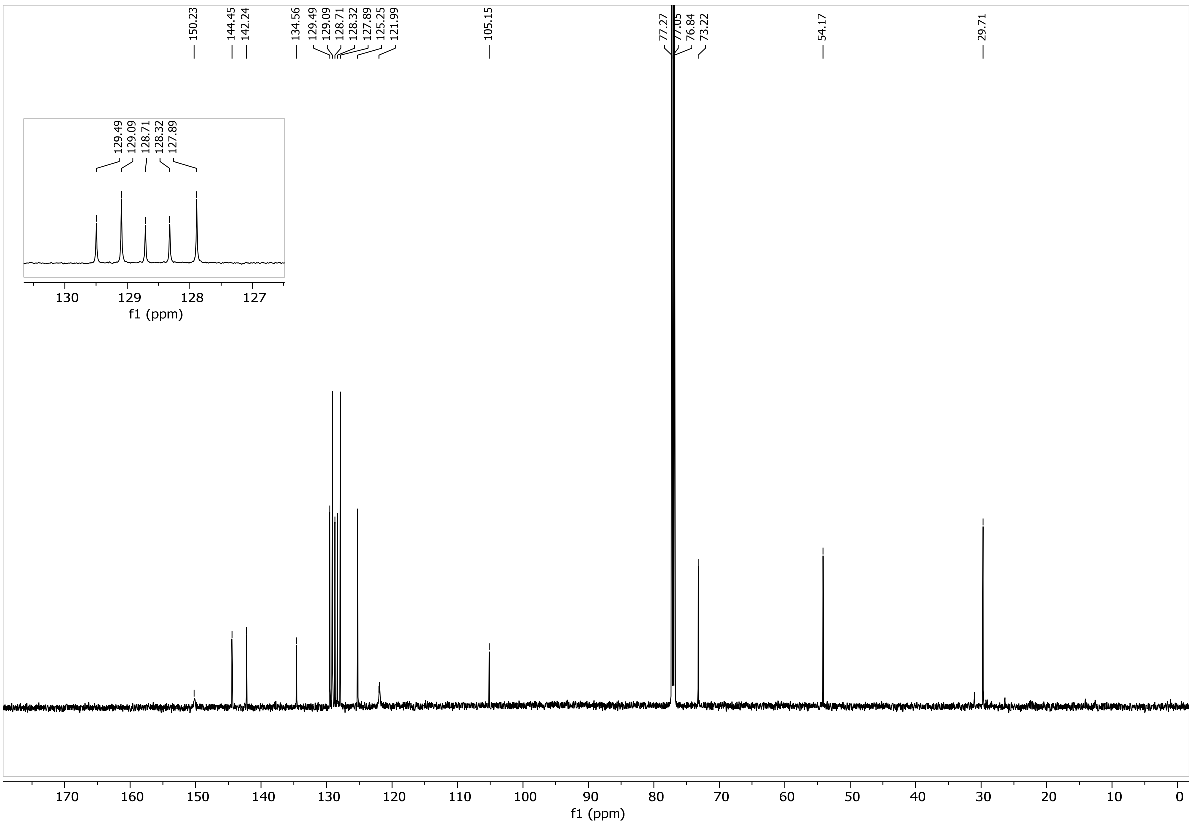

4.6. NMR of (*R*)-(1-benzyl-1*H*-1,2,3-triazol-4-yl)(2-iodo-3- methoxyphenyl) methanol (9c) in $CDCl_3$

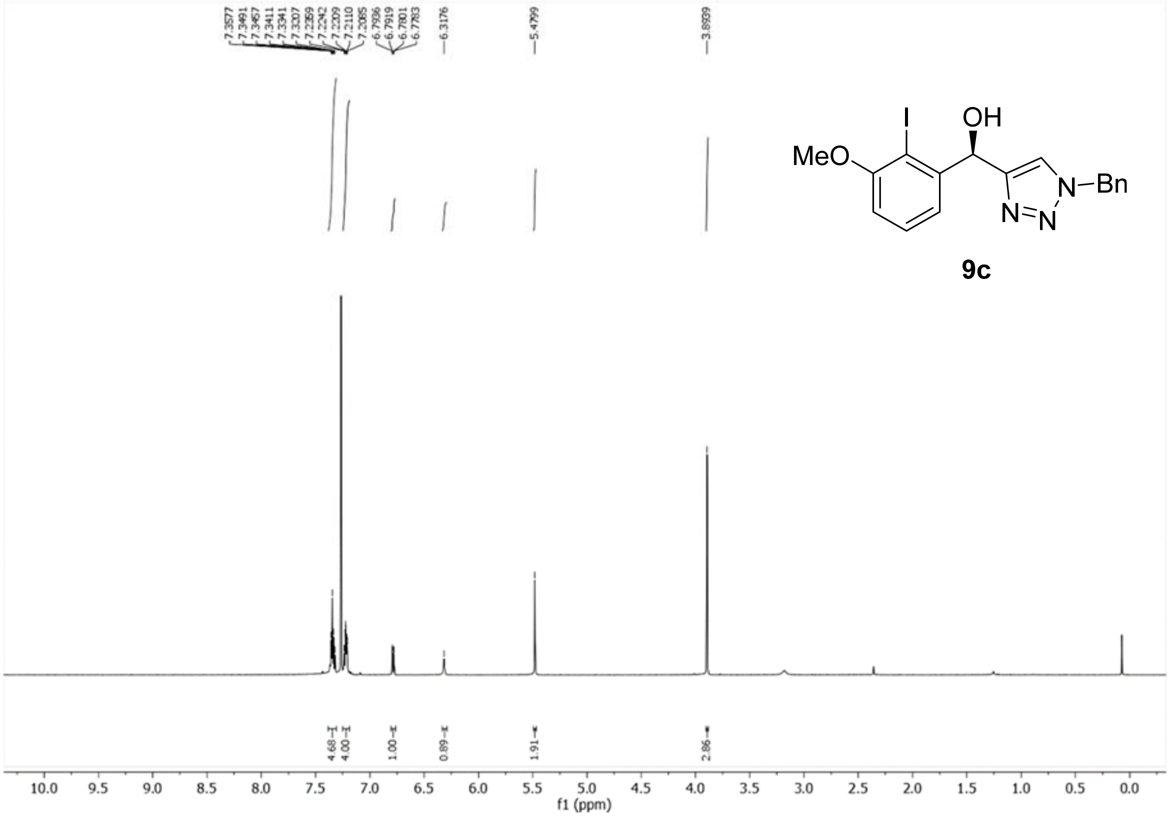

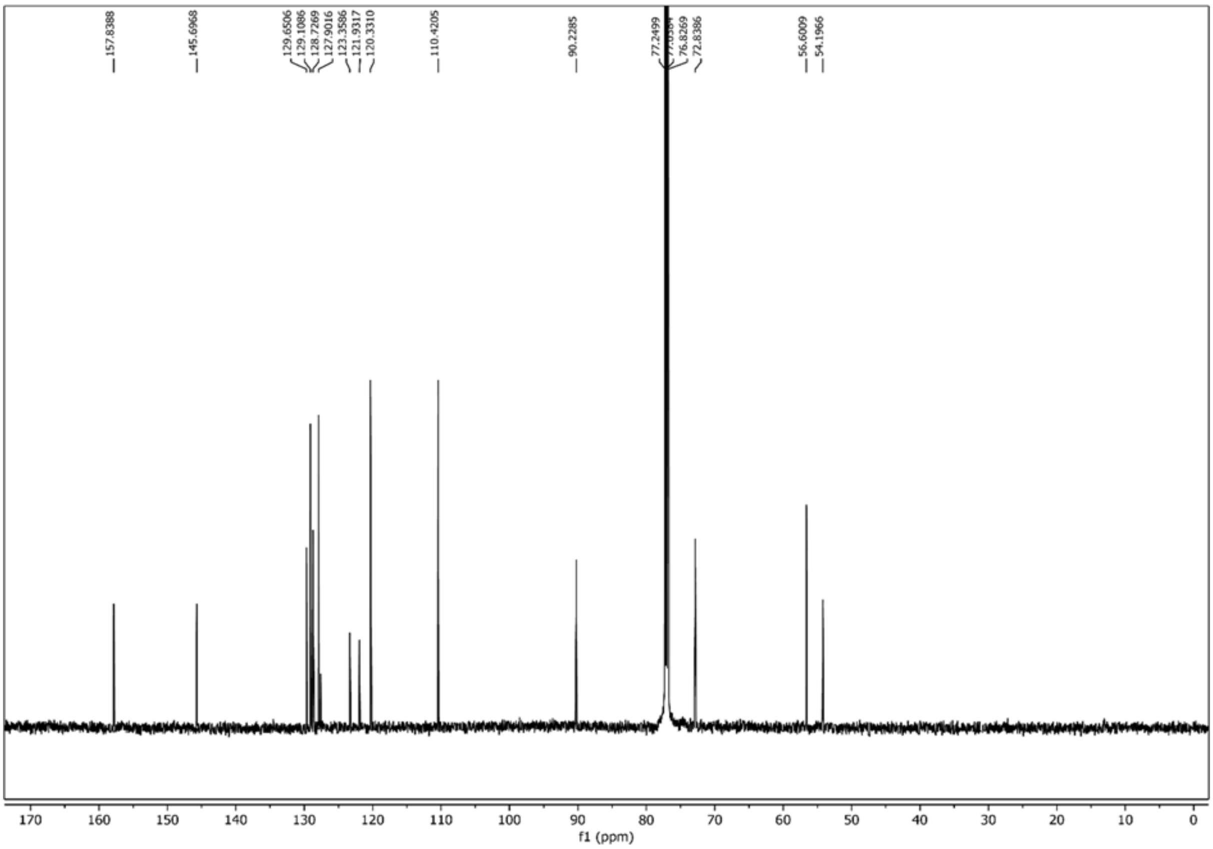

4.7. NMR of (*R*)-1-benzyl-4-((3-chloro-2-iodophenyl)((triisopropylsilyl)oxy)methyl)-1*H*-1,2,3-triazole (6a) in $CDCl_3$

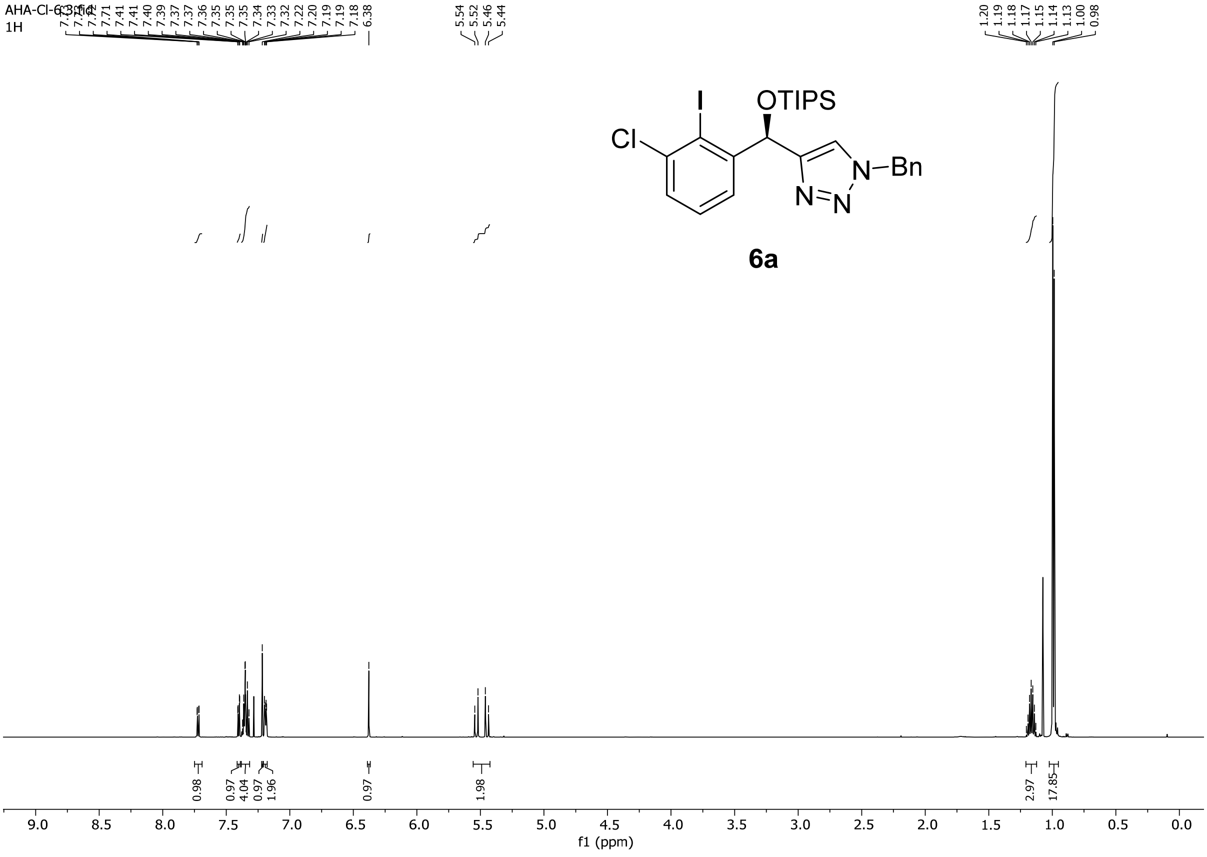

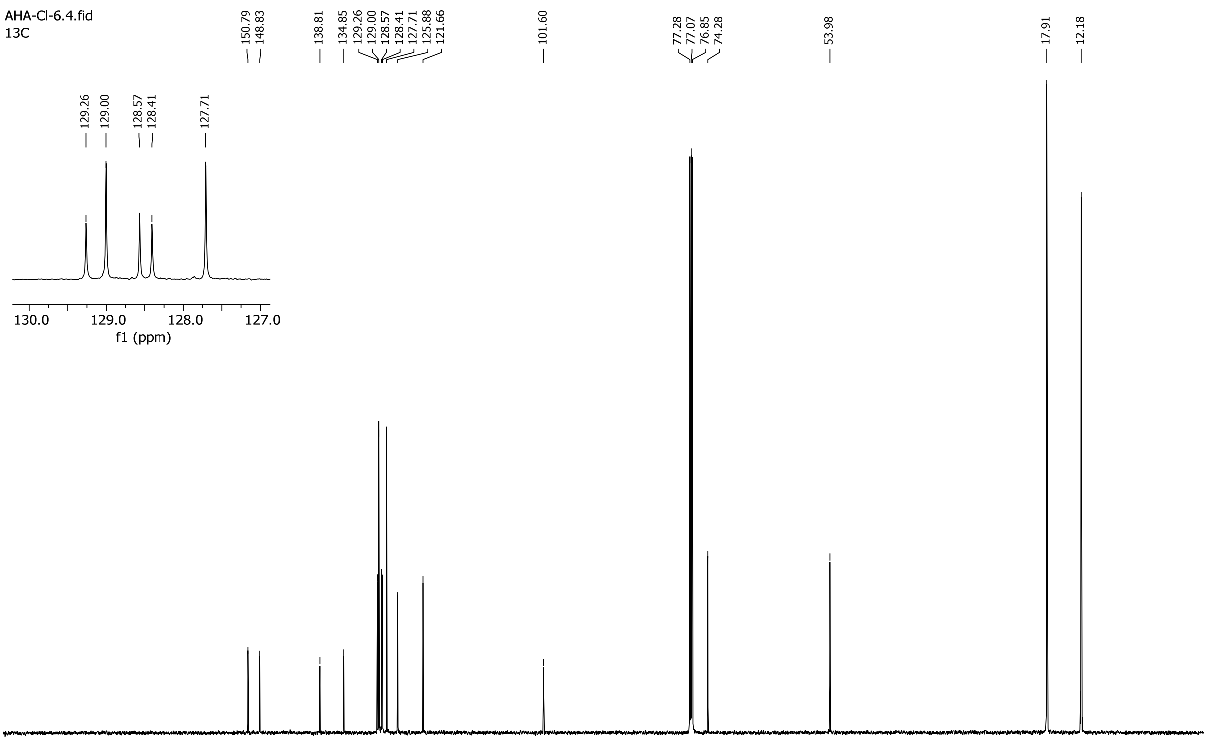

4.8. NMR of (*R*)-1-benzyl-4-((2-iodo-3- methylphenyl) ((triisopropylsilyl) oxy)methyl)-1*H*-1,2,3-triazole (6b) in $CDCl_3$

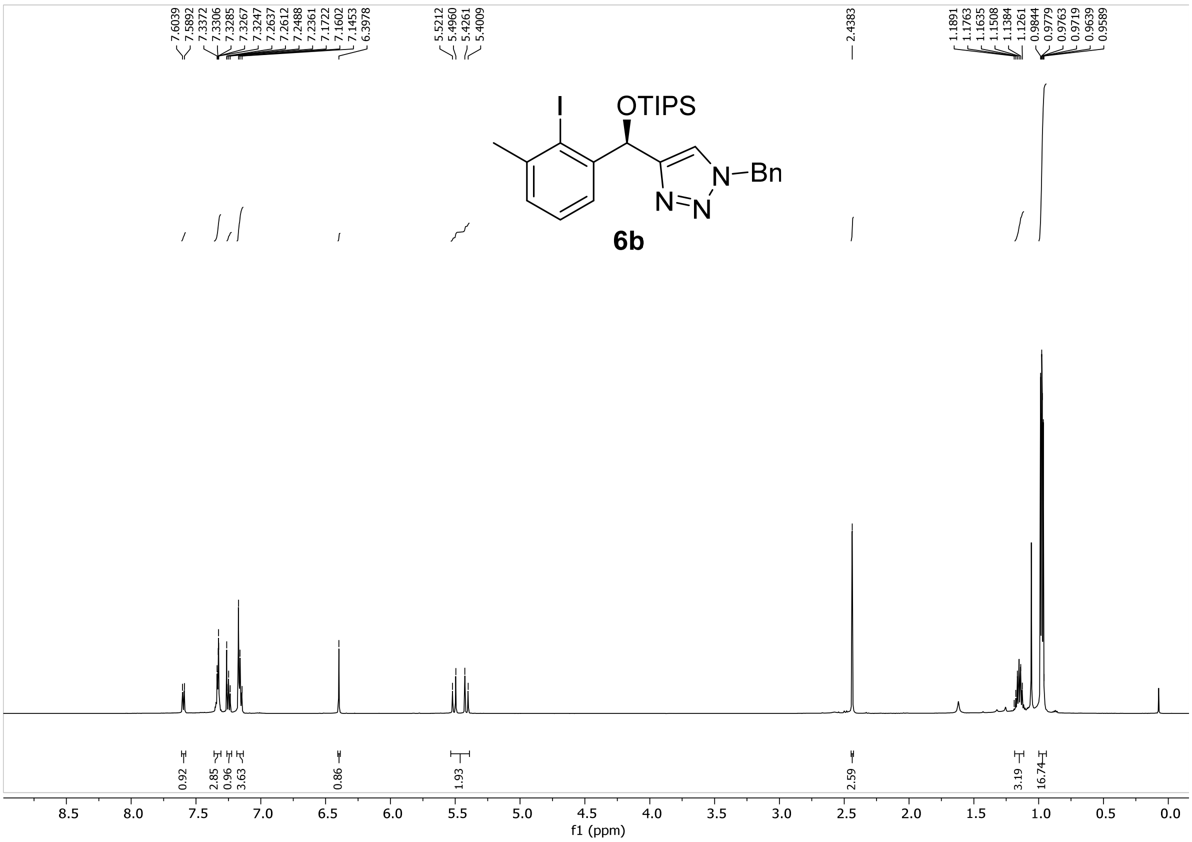

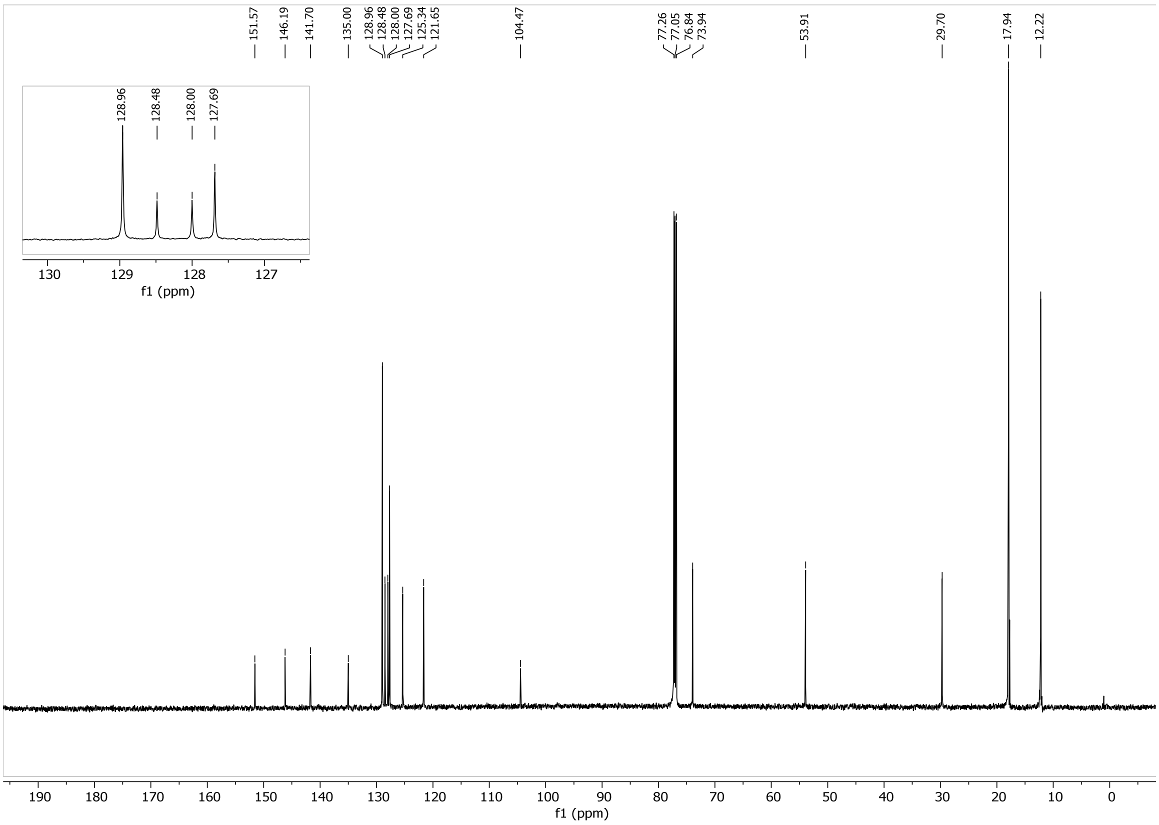

4.9. NMR of (*R*)-1-benzyl-4-((2-iodo-3 methoxyphenyl) ((triisopropylsilyl) oxy)methyl)-1*H*-1,2,3-triazole (6c) in $CDCl_3$

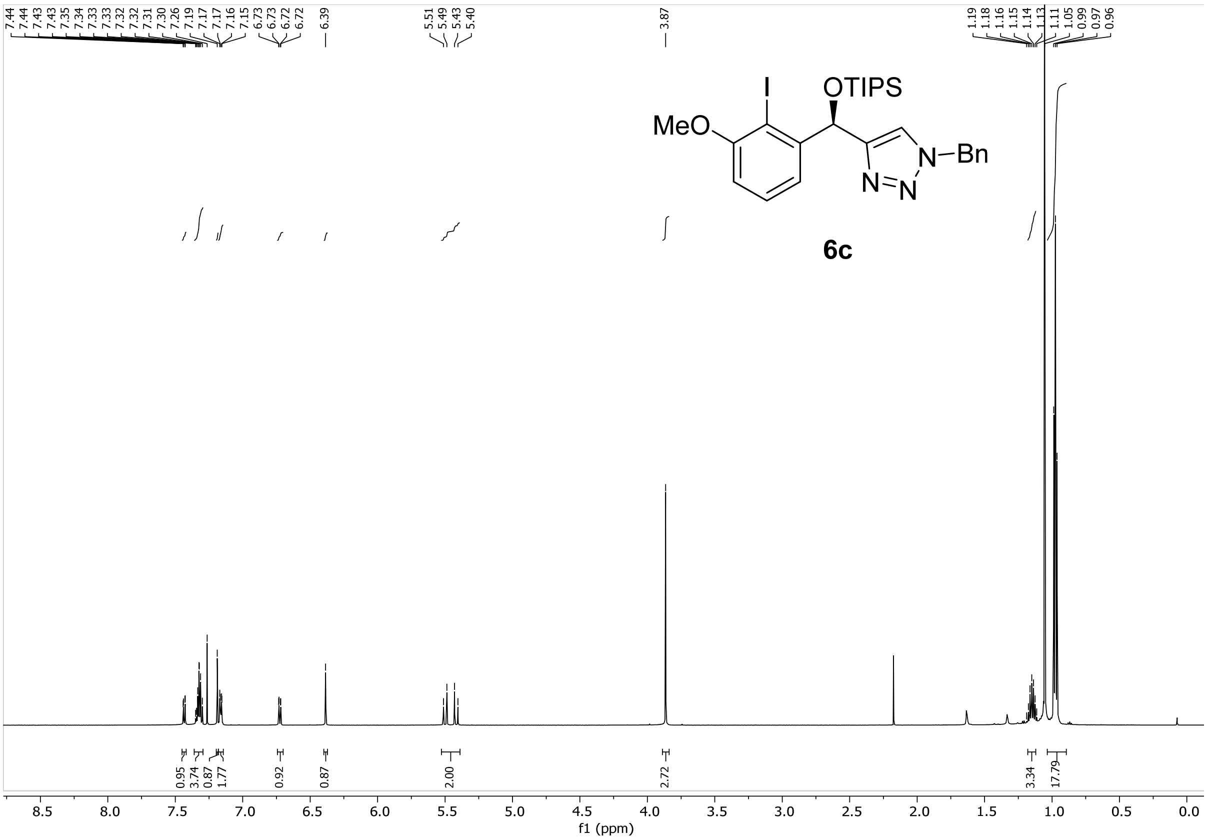

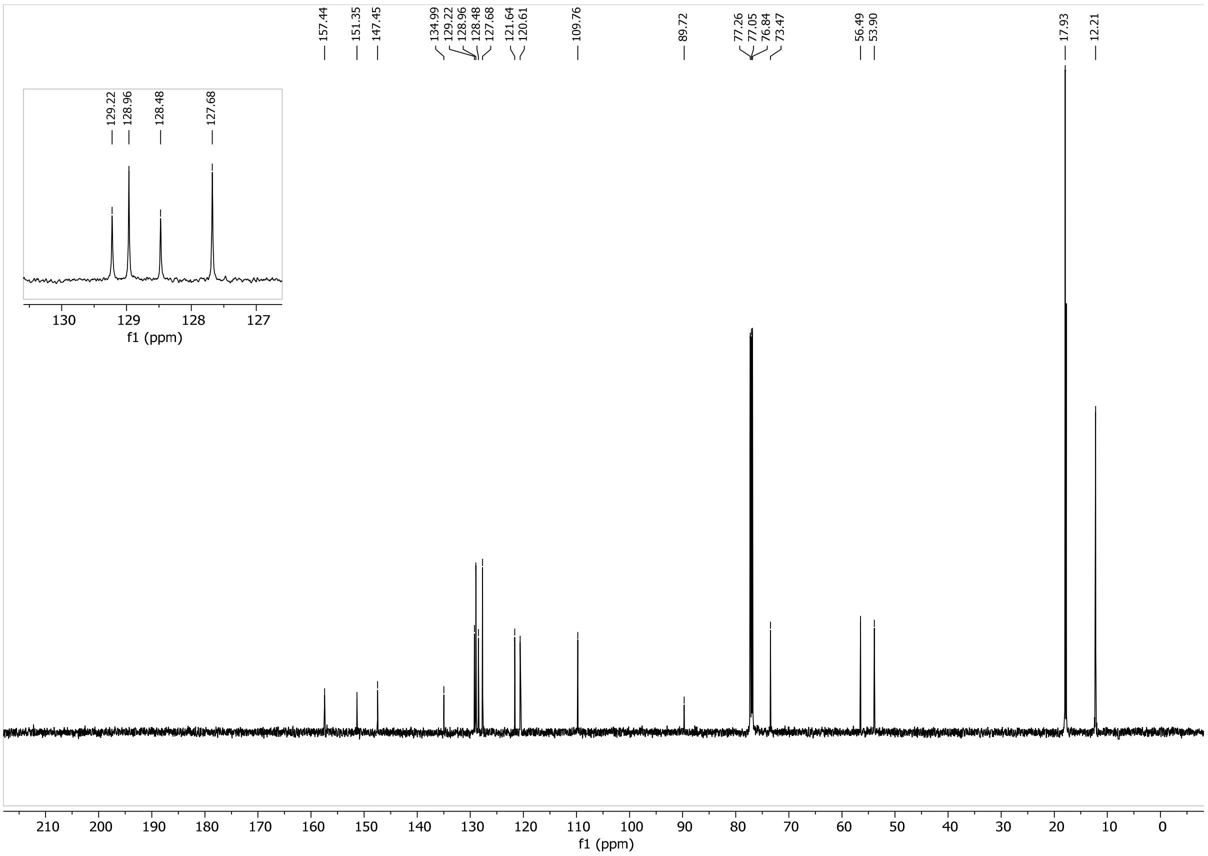

4.10. NMR of (*R*)-1-benzyl-4-((2-iodo-3-methoxyphenyl)((triisopropylsilyl)oxy)methyl)-3-methyl-1*H*-1,2,3-triazol-3-ium (6d) in $CDCl_3$

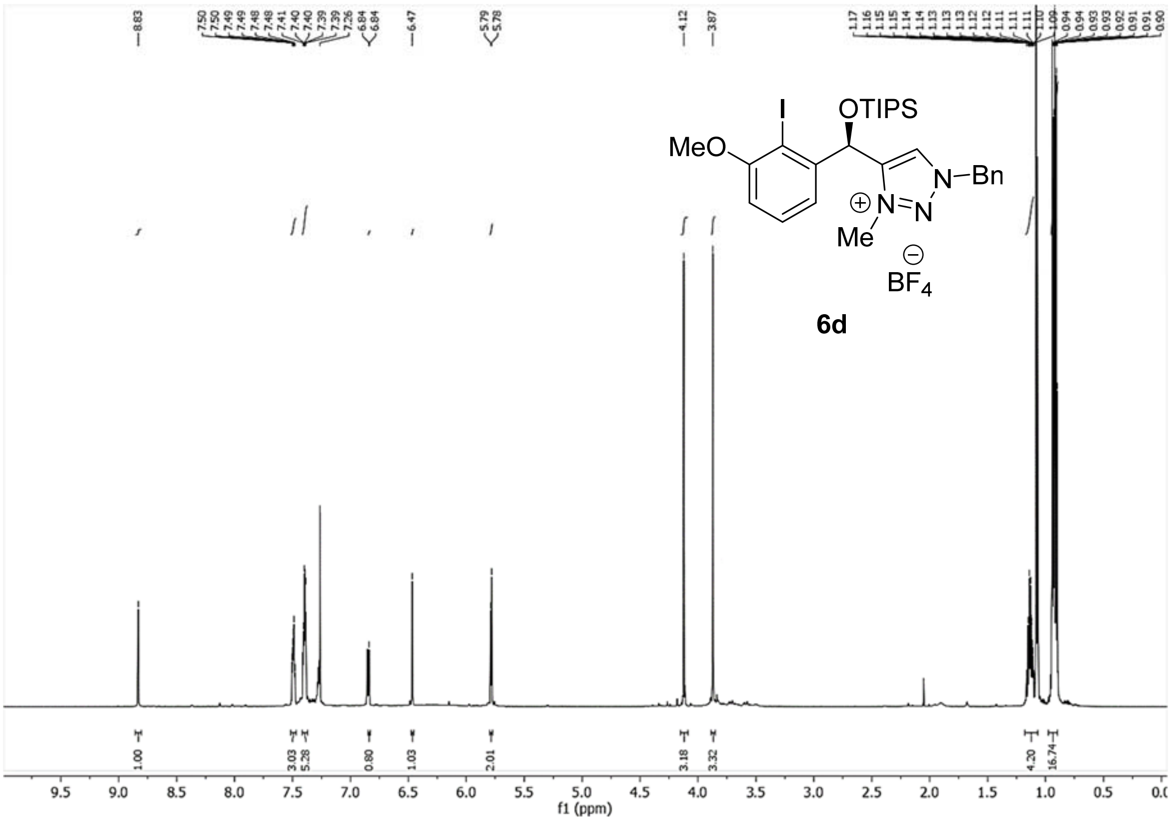

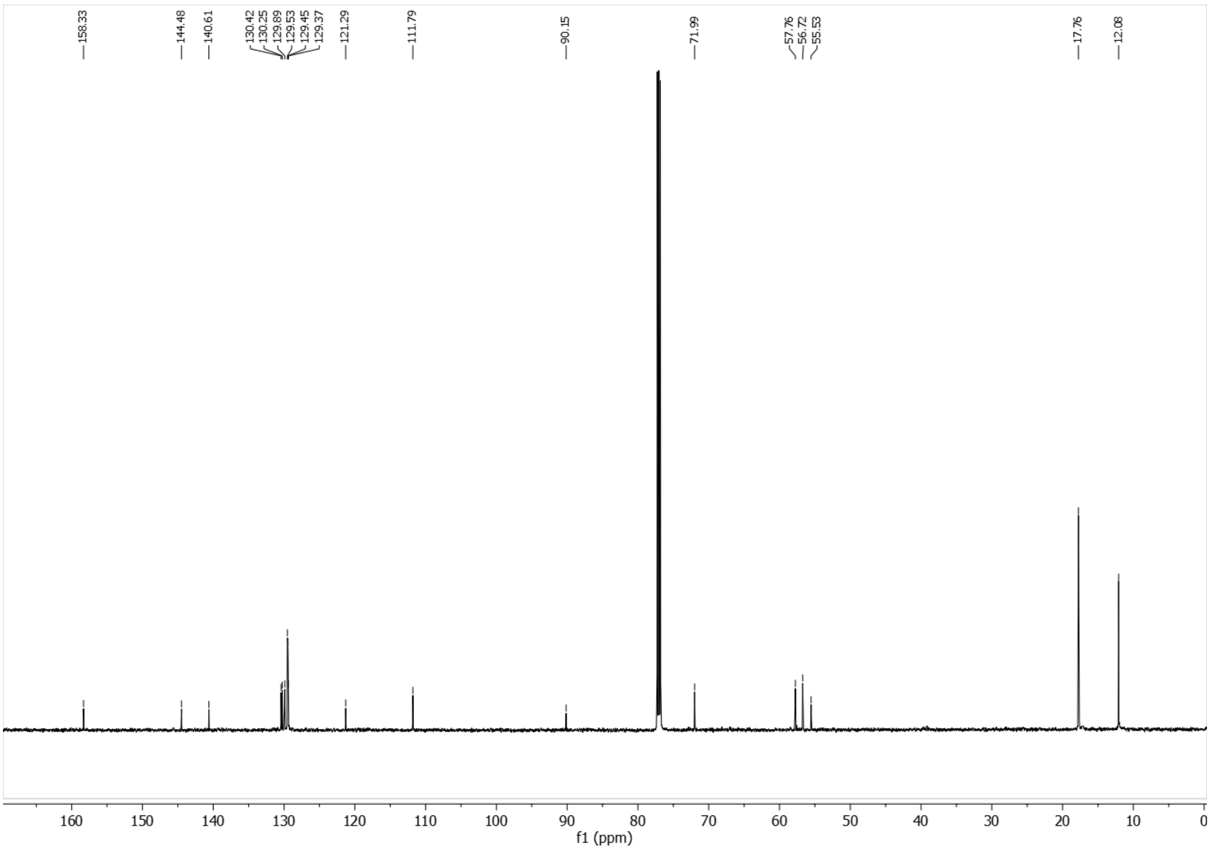

4.11. NMR of 3,4-dihydro-1'H,5H-spiro[furan-2,2'-naphthalene]-1',5- (11) in $CDCl_3$

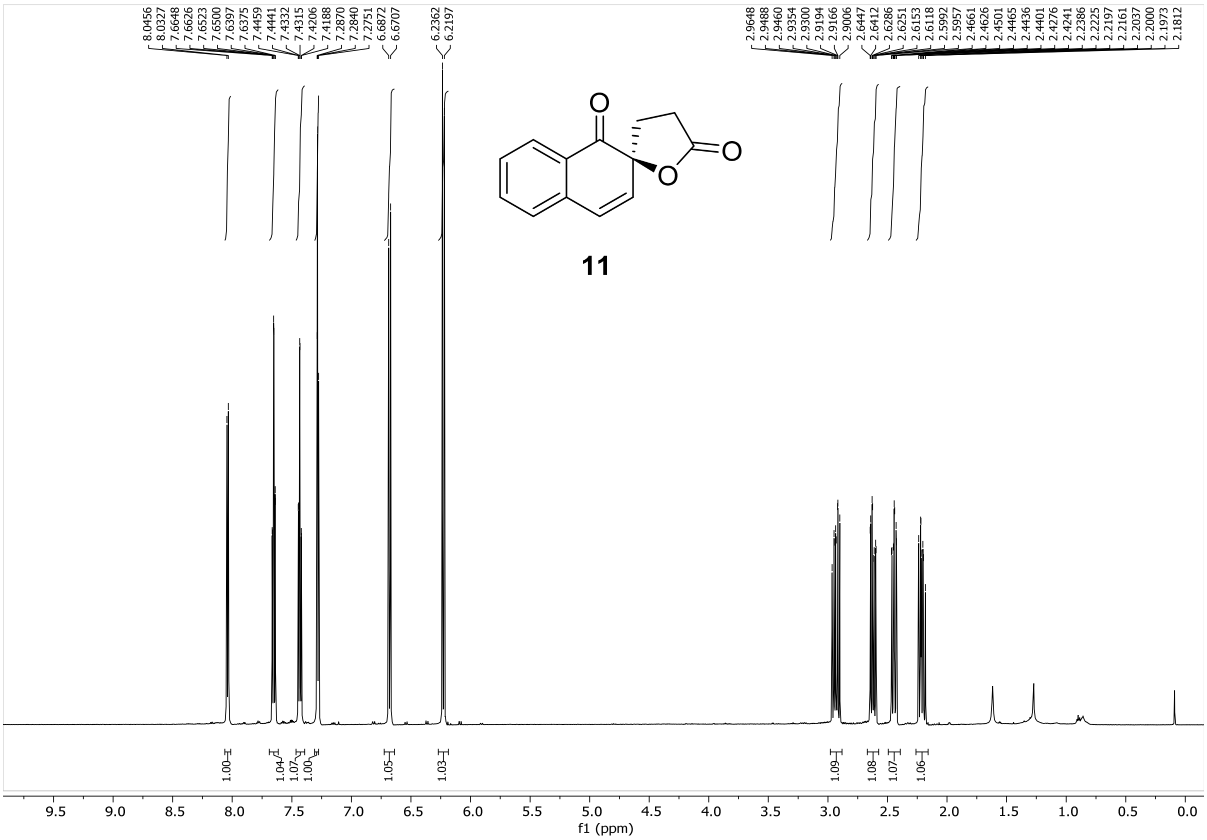

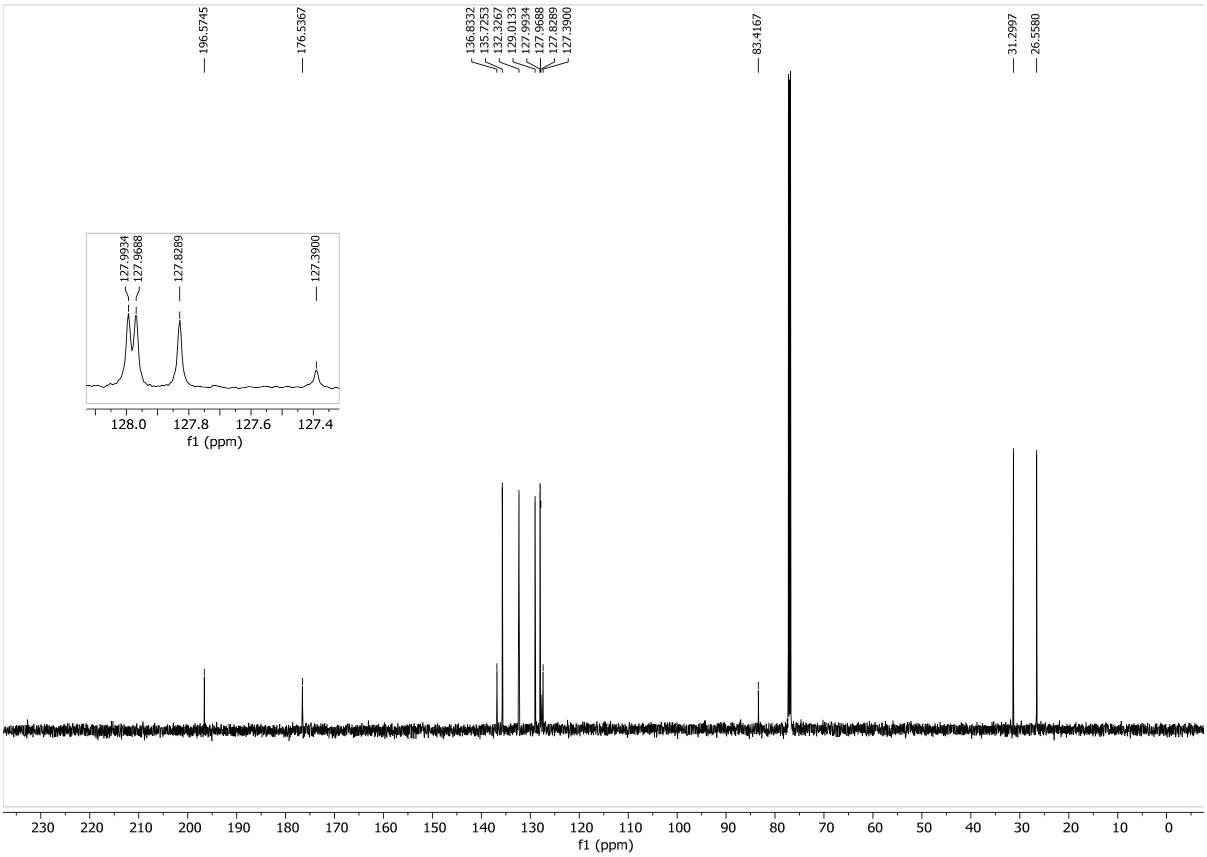

4.12. NMR of 2-bromo-4-hydroxy-4-methylcyclohexa-2,5-dien-1-one (13) in $CDCl_3$

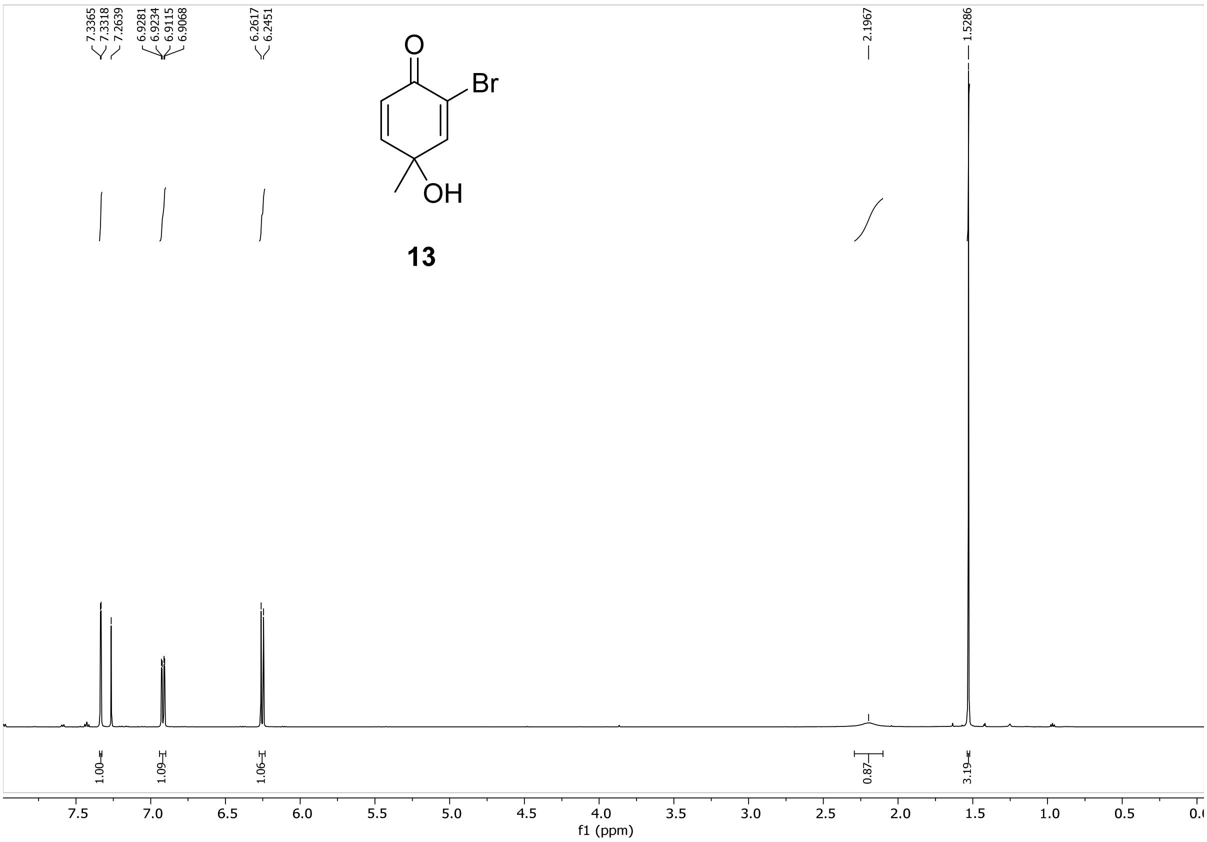

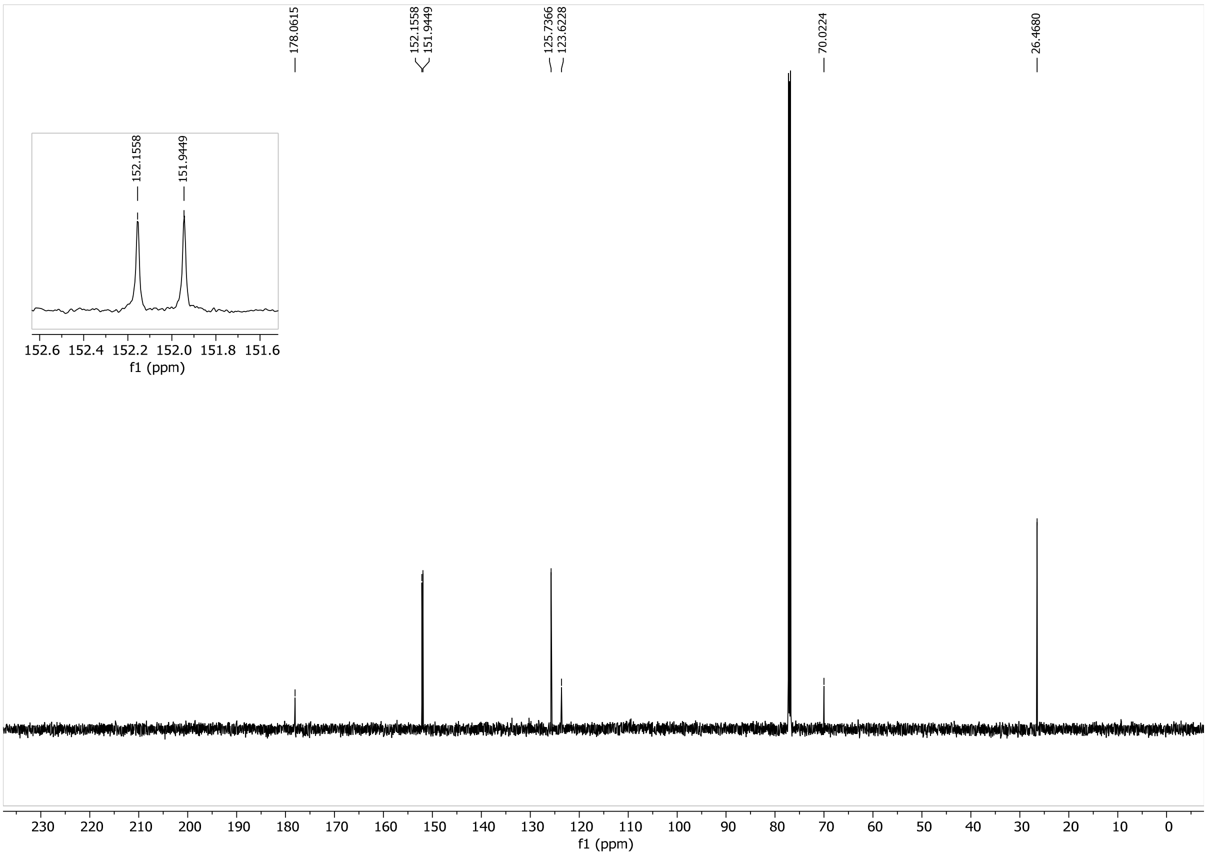

4.13. NMR of 5-benzoyldihydrofuran-2(3H)-one (15) in $CDCl_3$

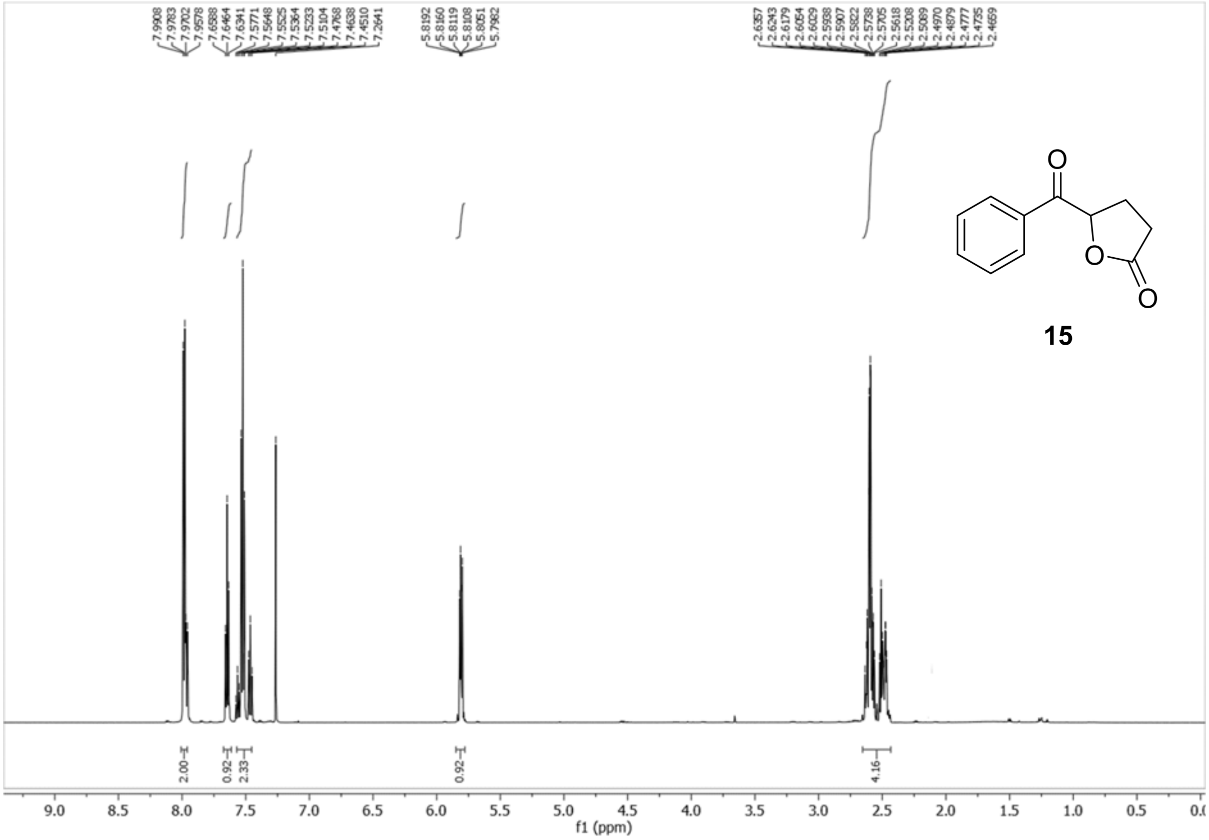

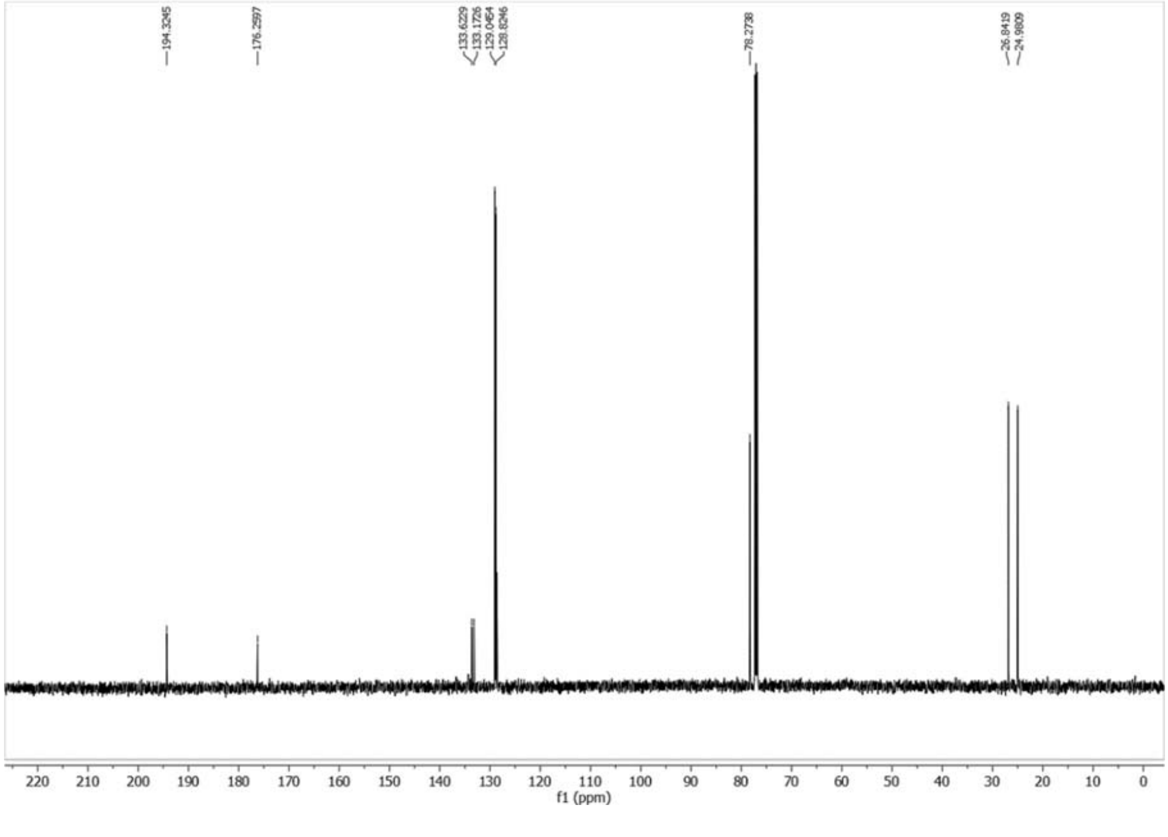

4.14. NMR of 1-oxo-1-phenylpropan-2-yl 4-methylbenzenesulfonate (17) in $CDCl_3$

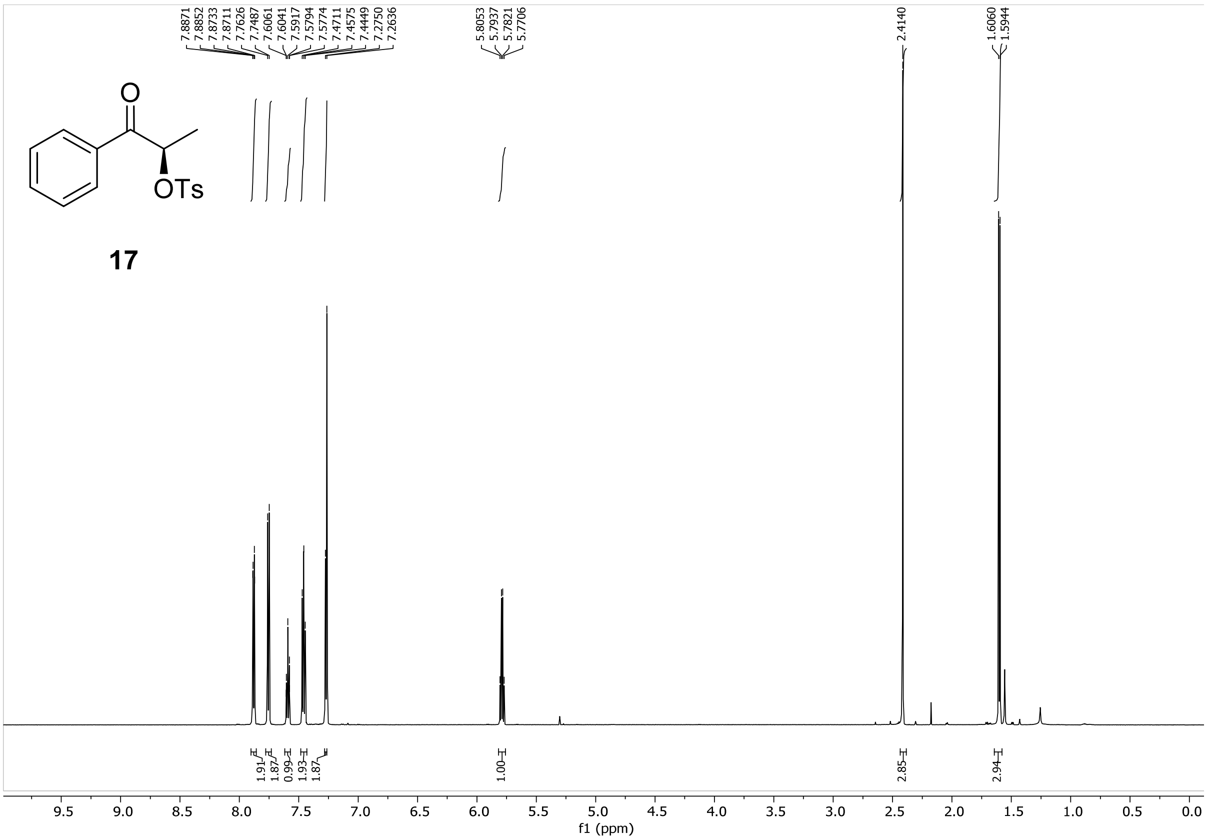

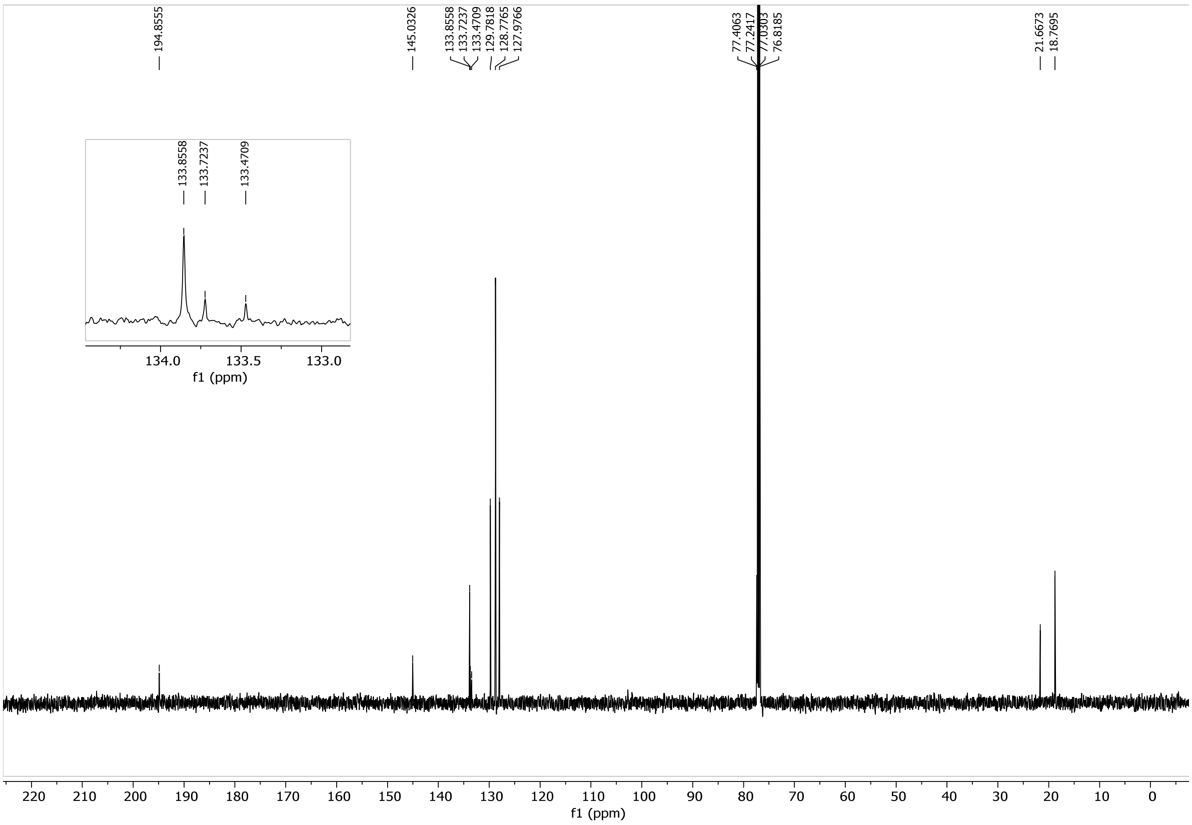

4.15. NMR of 1,1-diphenylprop-2-en-1-ol (19) in $CDCl_3$

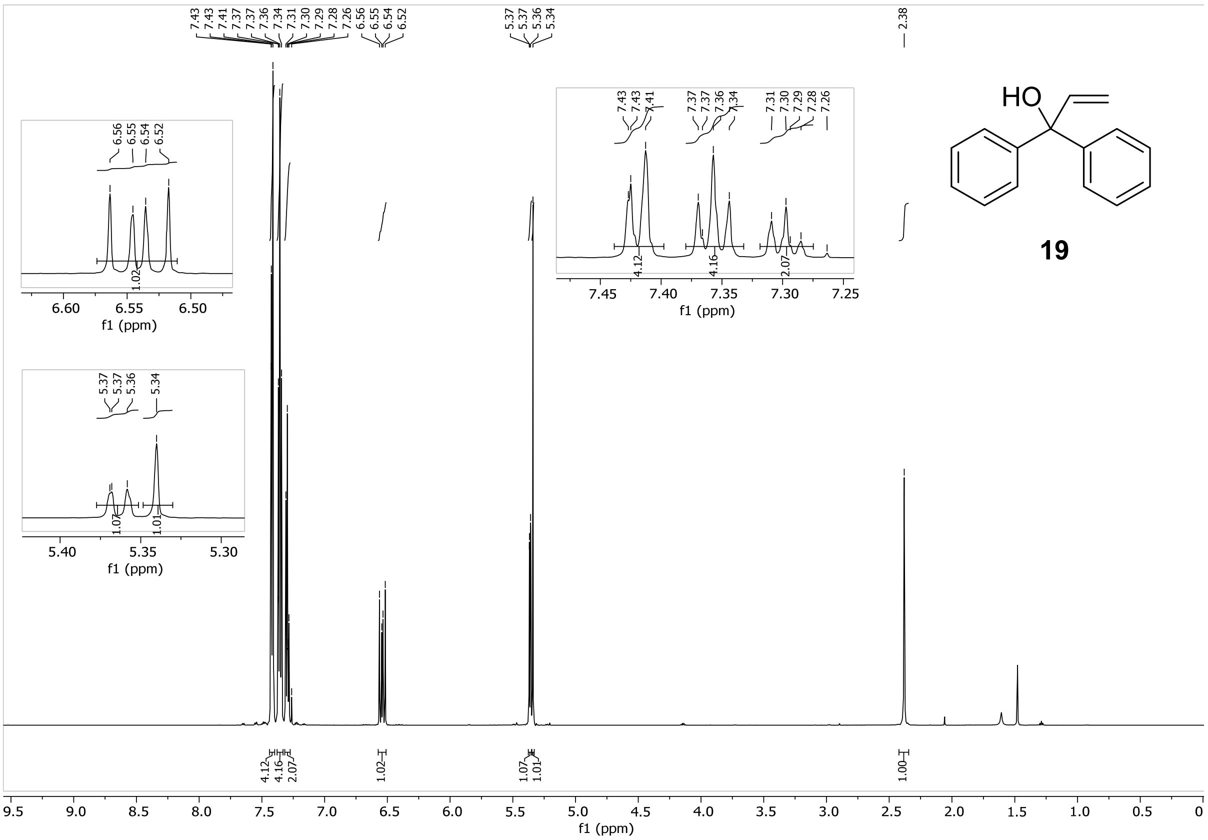

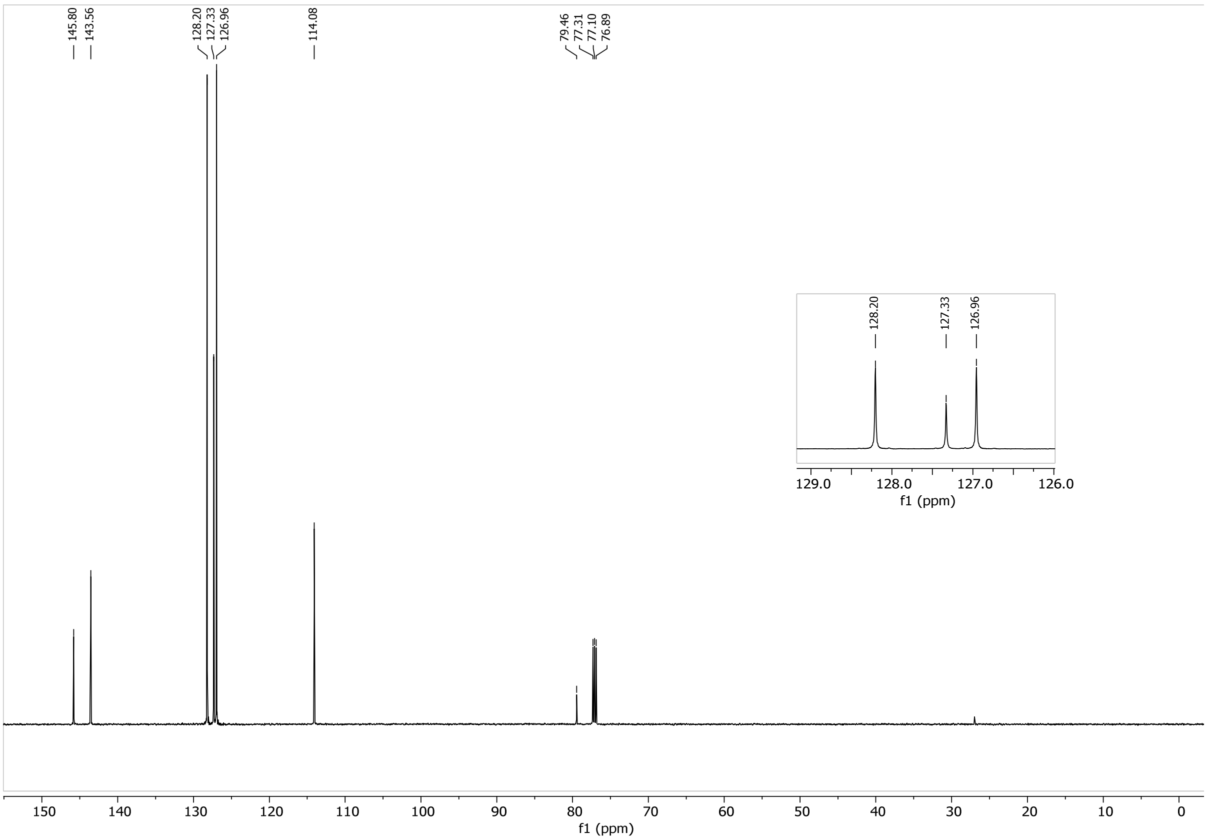

4.16. NMR of (*S*)-3-(benzyloxy)-1,2-diphenylpropan-1-one (20) in $CDCl_3$

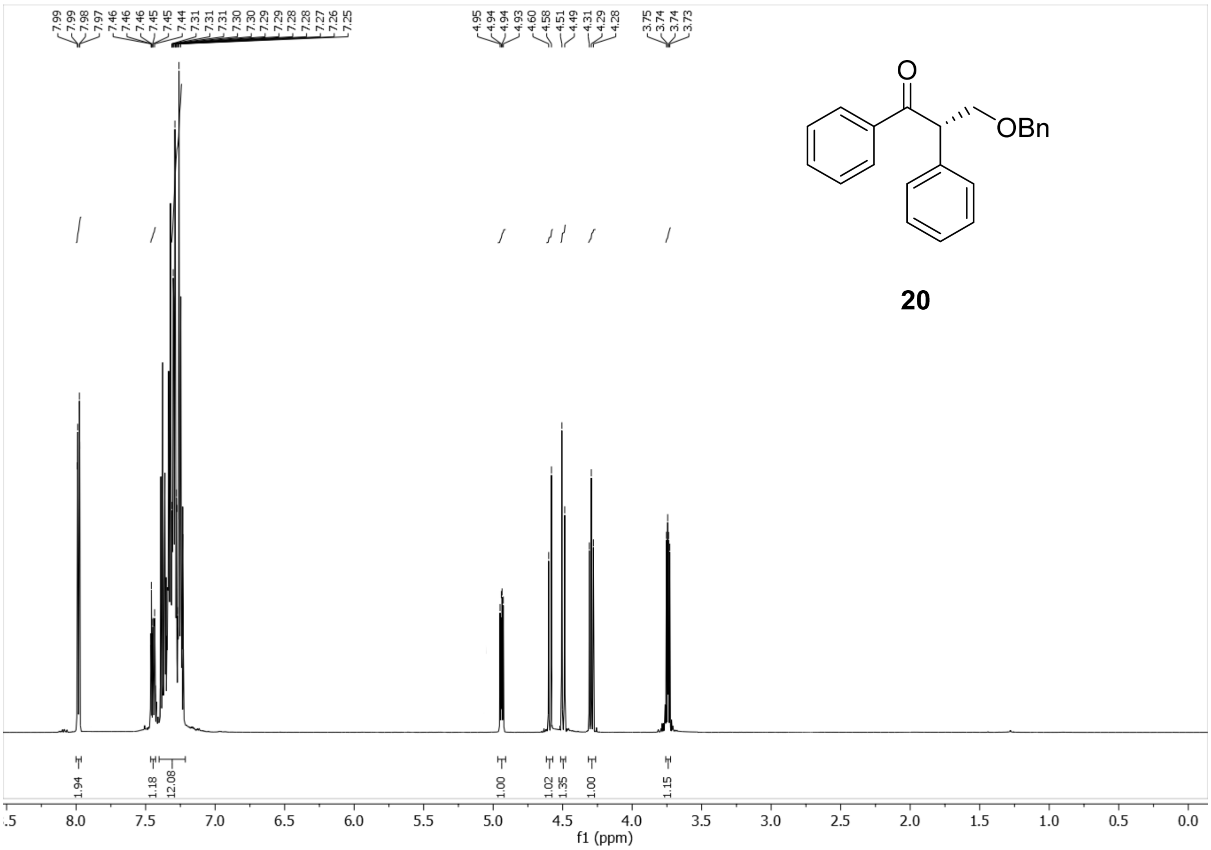

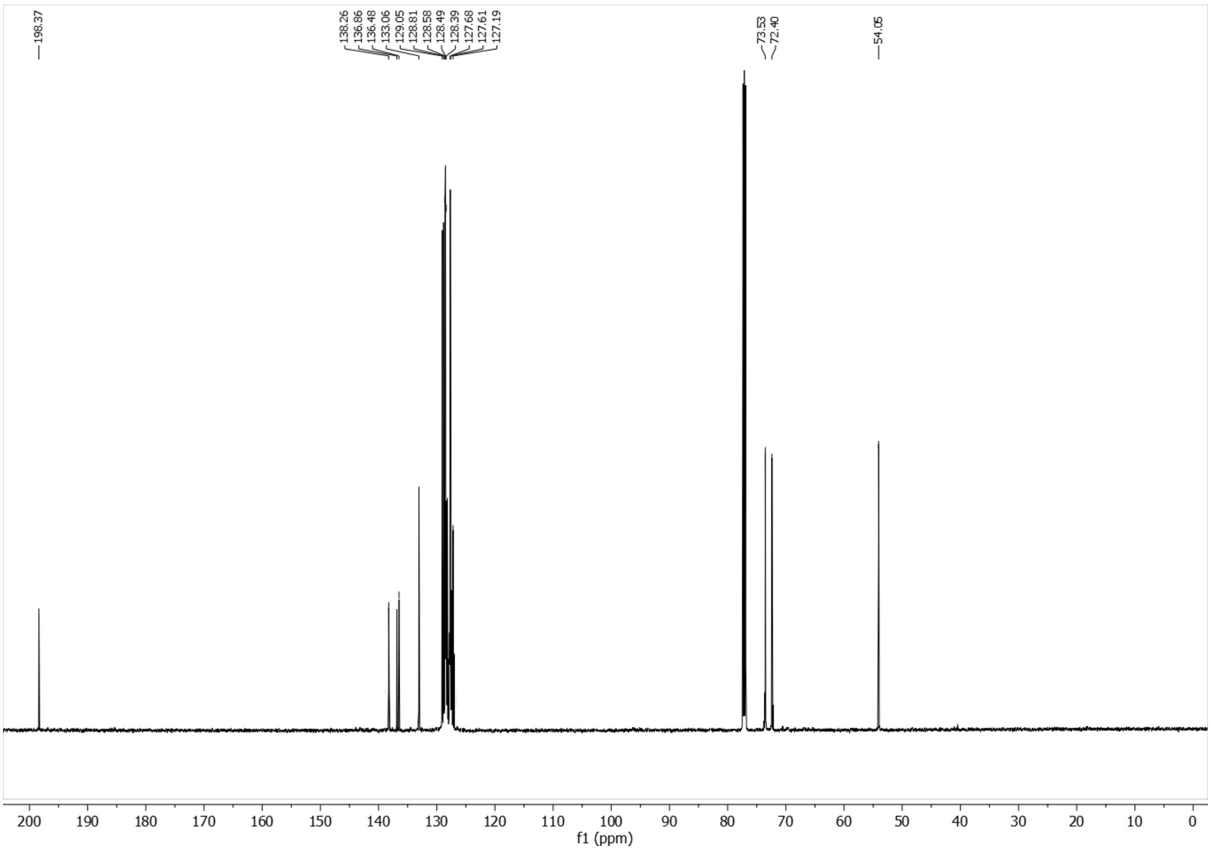

5. HPLC Chromatograms

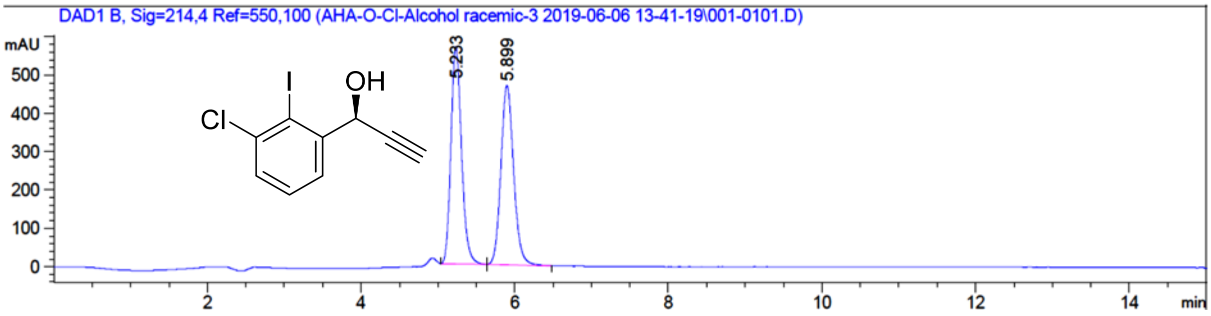

Peak #	RetTime [min]	Type	Width [min]	Area [mAU*s]	Height [mAU]	Area %
1	5.233	BB	0.1466	5422.77686	567.09229	50.1677
2	5.899	BB	0.1761	5386.52051	468.60800	49.8323

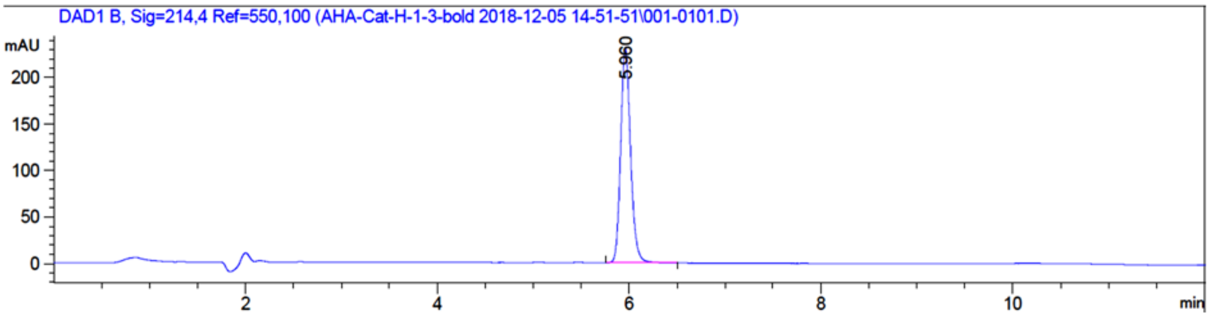

Peak #	RetTime [min]	Type	Width [min]	Area [mAU*s]	Height [mAU]	Area %
1	5.960	BB	0.1100	1658.67871	231.71870	100.0000

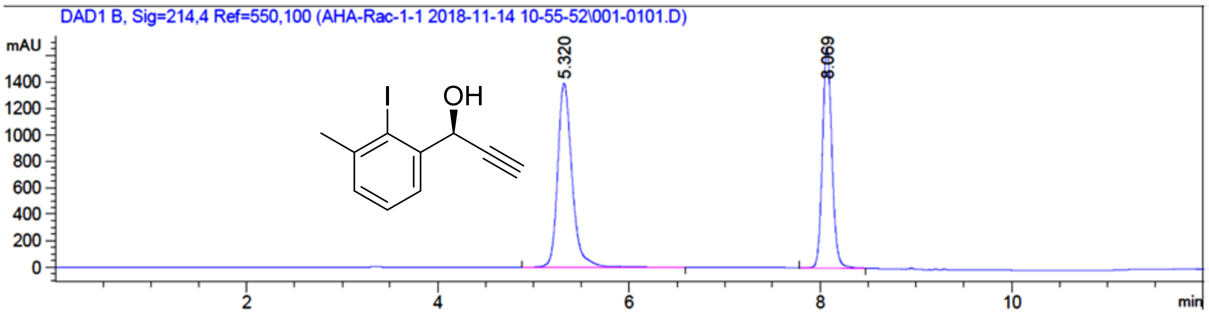

Peak #	RetTime [min]	Type	Width [min]	Area [mAU*s]	Height [mAU]	Area %
1	5.320	BB	0.1597	1.45177e4	1391.96545	55.9275
2	8.069	BB	0.1064	1.14403e4	1669.70959	44.0725

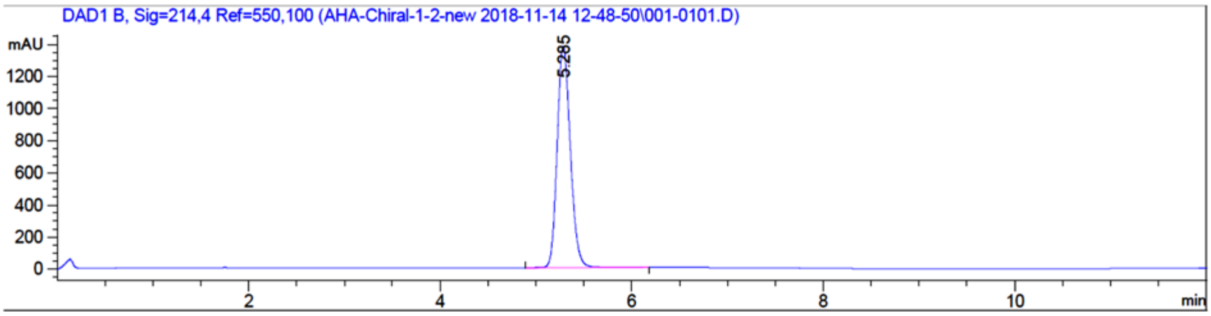

Peak #	RetTime [min]	Type	Width [min]	Area [mAU*s]	Height [mAU]	Area %
1	5.285	BB	0.1481	1.32003e4	1380.33459	100.0000

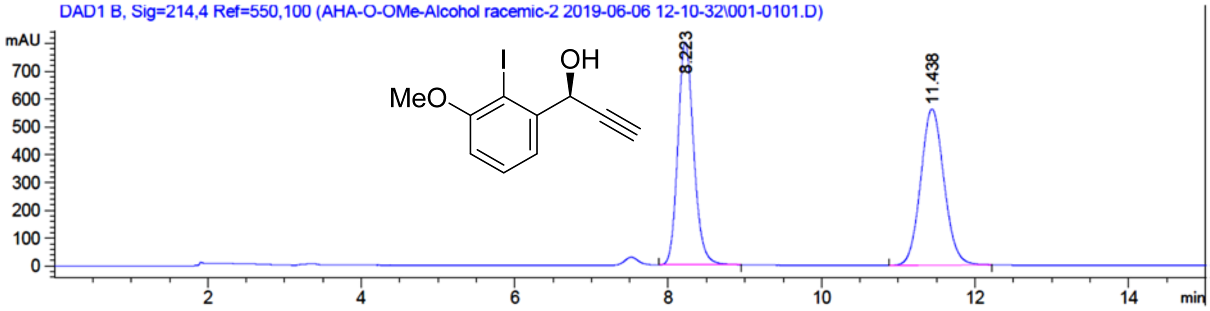

Peak #	RetTime [min]	Type	Width [min]	Area [mAU*s]	Height [mAU]	Area %
1	8.223	BB	0.2219	1.14641e4	801.33411	49.1239
2	11.438	BB	0.3137	1.18730e4	561.50305	50.8761

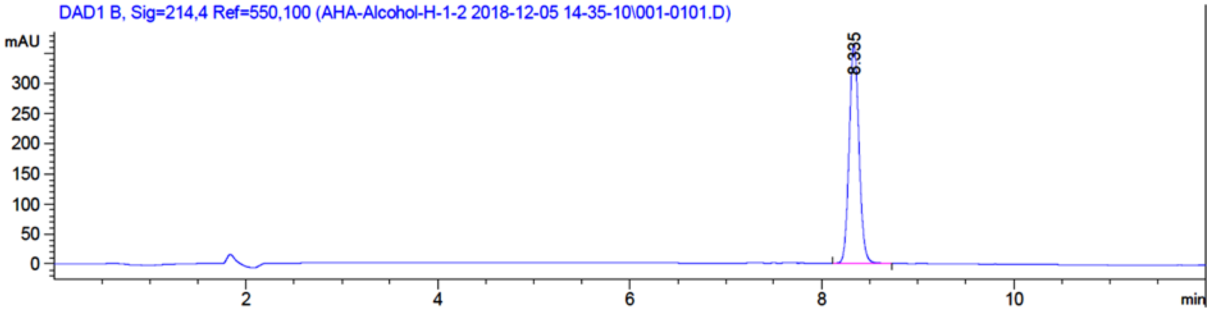

Peak #	RetTime [min]	Type	Width [min]	Area [mAU*s]	Height [mAU]	Area %
1	8.335	BB	0.1093	2578.35815	365.33478	100.0000

6. CD Spectra

Spectrum as calculated by Orca Spectrum

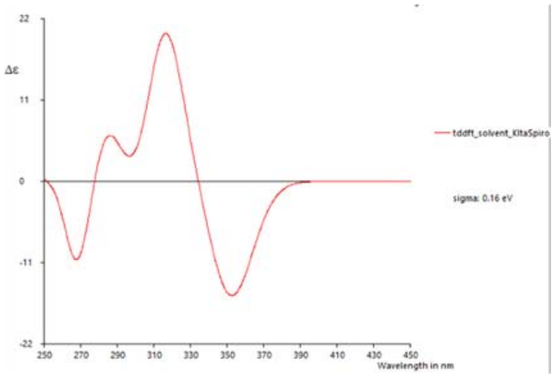

Measured CD Spectrum in CHCl3

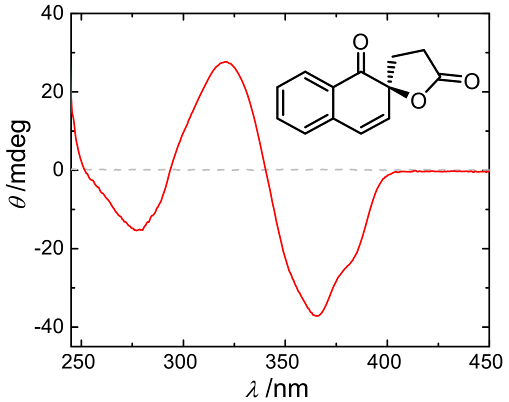

7. Computational Studies

7.1. Computational Details

All quantum chemical calculations were performed with ORCA 4.1 and Orca 4.2. Geometries were pre-optimized with Grimme's tight binding method GFN2-xTB.[7] Local minima were further optimized with the composite method PBEh-3c[8] in the gas phase using the RIJCOSX approximation with the ma-def2-SVP[9] basis sets for oxygen and nitrogen and the def2-TZVP[10] basis set for iodine. Minimum structures were verified on the potential energy hyper surface by the absence of imaginary frequencies in the harmonic frequency calculation. The double hybrid functional PWPB95-D3[11] using the RIJCOSX approximation and the def2-TZVP basis sets with ma-def-TZVP basis sets for oxygen and nitrogen and the def2-TZVPP basis set for iodine applying the SDM solvation model for chloroform (ε = 4.71) [3] was used for single point calculations on the optimized geometries. Def2/J and the def2-TZVP/C auxiliary set were applied.[10, 12] The CD-spectrum to confirm the absolute configuration of the spirolactone (*R*)-**11** was calculated with TDDFT on a PBE0/D3BJ/def2-TZVP level of theory.

Calculated Single Point Energies and Thermodynamic Corrections[a]

**Compound**	**Final single point energy incl. dispersion and solvent correction** (PWPB95-D3/ma-def2-TZVP(O,N)/TZVPP(I)	**Total Entropy Correction at 298.15 K** to the (PBEh3c/ma-def2-SVP(O,N)/TZVP(I)	**Total thermal and non-thermal corrections to the electronic energy and thermal enthalpy corrections at 298.15 K** (PBEh3c/ma-def2-SVP(O,N)/TZVP(I)	**Free Gibbs Energy**
**5-OH-N**	-2769,64226530	-0,11814626	0,76125410	-2768,99915746
**5-OH-O**	-2769,64362520	-0,11726128	0,76104321	-2768,99984327
**5-OH-OTs1**	-2769,63705849	-0,12073132	0,76177451	-2768,99601530
**5-OH-OTs2**	-2769,63399840	-0,11801990	0,76158957	-2768,99042873
**6c-OH-N**	-2884,13540506	-0,12440181	0,79734138	-2883,46246549
**6c-OH-O**	-2884,12953886	-0,12397170	0,79711752	-2883,45639304
**6c-OH-OTs1**	-2884,13756531	-0,12453322	0,79812895	-2883,46396958
**6c-OH-OTs2**	-2884,13648420	-0,12369627	0,79767038	-2883,46251009
**6c-OH$^+$**	-1989,28833029	-0,10073488	0,64983380	-1988,73923137
**6c-OH-N$^+$**	-1989,28585402	-0,10146378	0,64978811	-1988,73752969
**TsO$^-$**	-894,80518127	-0,04682318	0,14569600	-894,70630845

[a]All values are given in Eh.

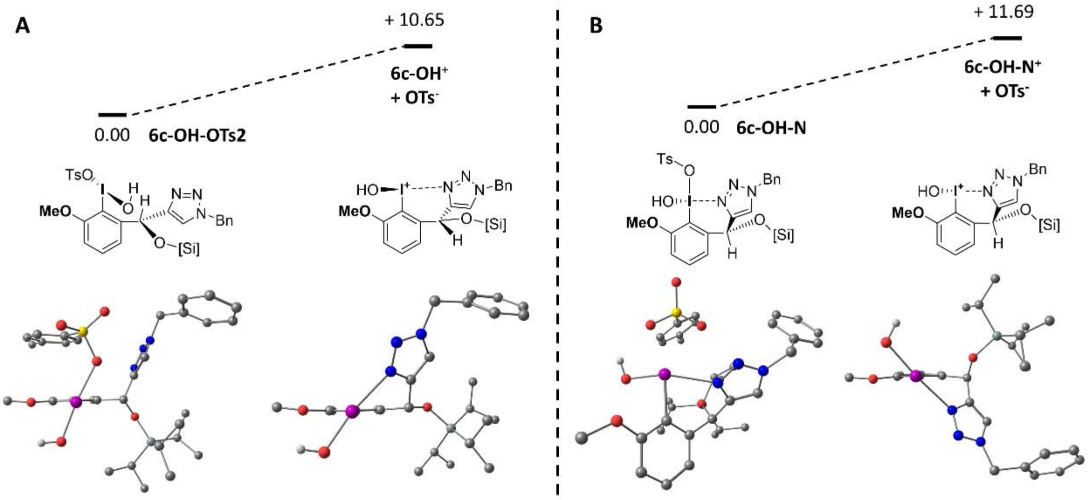

Figure S1 – Relative Gibb free energies for the dissociation of tosylate from complex **6c-OH-OTs 2(A)** and **6c-OH-N (B)**.

a. Coordinates

5-OH-N

6	3.521522000	0.666893000	0.605534000
6	2.578451000	1.310240000	1.394657000
6	1.299042000	0.795484000	1.502295000
6	0.964050000	-0.361159000	0.815289000
6	1.885803000	-1.020716000	0.012722000
6	3.174153000	-0.486383000	-0.079146000
53	4.637603000	-1.400977000	-1.273346000
6	1.439477000	-2.266895000	-0.732346000
6	2.264495000	-3.445392000	-0.310429000
7	3.600120000	-3.313209000	-0.213659000
7	4.158513000	-4.429111000	0.059481000
7	3.206452000	-5.316056000	0.151535000
6	1.993588000	-4.763797000	-0.074186000
6	3.531881000	-6.722095000	0.325153000
6	3.329040000	-7.488081000	-0.954673000
6	2.461971000	-8.571752000	-0.992158000
6	2.275491000	-9.279996000	-2.171201000
6	2.953552000	-8.900184000	-3.318281000
6	3.818390000	-7.814325000	-3.283451000
6	4.010885000	-7.108278000	-2.107937000
8	1.590767000	-2.088120000	-2.092932000

14	0.587540000	-2.140463000	-3.450732000
6	-1.184823000	-2.438411000	-2.821547000
6	-2.258116000	-2.085690000	-3.854760000
6	-1.404517000	-3.863148000	-2.305417000
6	0.824007000	-0.428305000	-4.222164000
6	0.366281000	-0.302579000	-5.675207000
6	0.232445000	0.679921000	-3.349774000
6	1.262995000	-3.494061000	-4.585593000
6	1.816966000	-4.709287000	-3.841655000
6	0.277239000	-3.930288000	-5.672291000
1	2.853430000	2.211777000	1.925135000
1	0.559406000	1.292419000	2.115419000
1	-0.041452000	-0.757308000	0.894913000
1	0.395697000	-2.460205000	-0.451483000
1	1.082574000	-5.337169000	-0.075588000
1	2.917338000	-7.125294000	1.130999000
1	4.571017000	-6.750659000	0.652205000
1	1.930469000	-8.871325000	-0.095911000
1	1.600366000	-10.125182000	-2.191385000
1	2.809269000	-9.449289000	-4.239390000
1	4.351277000	-7.511304000	-4.174817000
1	4.683909000	-6.257685000	-2.104631000
1	-1.320173000	-1.748489000	-1.976167000
1	-2.178994000	-2.700184000	-4.752654000
1	-2.204735000	-1.042717000	-4.166455000
1	-3.258382000	-2.246968000	-3.444562000
1	-1.339276000	-4.594834000	-3.111663000
1	-2.395043000	-3.968657000	-1.855245000
1	-0.674639000	-4.162758000	-1.550485000
1	1.915548000	-0.302978000	-4.217233000
1	-0.709516000	-0.448108000	-5.788905000
1	0.867082000	-1.022641000	-6.323777000
1	0.593928000	0.692751000	-6.065648000
1	-0.857614000	0.624966000	-3.302279000
1	0.483289000	1.665907000	-3.748439000
1	0.611304000	0.645519000	-2.326927000
1	2.112723000	-3.019142000	-5.089933000
1	1.055012000	-5.225006000	-3.251829000
1	2.640597000	-4.444185000	-3.181361000
1	2.211582000	-5.438958000	-4.553051000
1	-0.566615000	-4.486580000	-5.258752000
1	0.773154000	-4.592823000	-6.386408000
1	-0.132812000	-3.094424000	-6.241598000
8	5.126256000	0.404991000	-1.860888000
1	5.854522000	0.340407000	-2.487380000
6	4.639362000	-1.602205000	-5.548171000

6	3.824458000	-1.457158000	-6.664375000
6	3.472796000	-2.549050000	-7.446732000
6	3.971409000	-3.801749000	-7.087764000
6	4.787557000	-3.957525000	-5.982923000
6	5.118943000	-2.853938000	-5.204631000
16	6.155612000	-3.105033000	-3.769876000
8	7.351928000	-3.781397000	-4.235998000
8	6.397861000	-1.734295000	-3.223729000
8	5.327929000	-3.878988000	-2.821850000
6	2.567241000	-2.399018000	-8.633191000
1	4.905473000	-0.733913000	-4.961855000
1	3.460885000	-0.470822000	-6.927310000
1	3.720752000	-4.668339000	-7.688570000
1	5.175027000	-4.935822000	-5.729651000
1	2.992268000	-2.866001000	-9.522292000
1	2.381313000	-1.351524000	-8.866800000
1	1.599831000	-2.872105000	-8.451897000
1	4.518035000	1.078010000	0.525065000

5-OH-O

6	4.662230000	-1.581036000	-3.743999000
6	5.318313000	-0.671491000	-2.928884000
6	4.877569000	-0.448411000	-1.632810000
6	3.776449000	-1.131108000	-1.143915000
6	3.101484000	-2.049487000	-1.938979000
6	3.565513000	-2.249210000	-3.227457000
53	2.637769000	-3.699114000	-4.413042000
6	1.947450000	-2.839811000	-1.367459000
6	2.439756000	-3.962121000	-0.513822000
7	2.666997000	-3.811407000	0.804923000
7	3.074589000	-4.928725000	1.286809000
7	3.119791000	-5.803045000	0.304031000
6	2.738155000	-5.254144000	-0.858165000
6	3.486100000	-7.191019000	0.523593000
6	2.374621000	-8.097958000	0.071182000
6	1.249045000	-8.275123000	0.870338000
6	0.190749000	-9.051256000	0.426732000
6	0.250088000	-9.658074000	-0.821241000
6	1.369156000	-9.484965000	-1.620537000
6	2.429443000	-8.706587000	-1.176549000
8	1.166725000	-3.330422000	-2.428670000
14	-0.562139000	-3.455137000	-2.499418000
6	-1.225370000	-3.461928000	-0.722535000
6	-2.702911000	-3.056183000	-0.660717000
6	-1.014057000	-4.788338000	0.014042000
6	-1.023673000	-1.899494000	-3.482267000
6	-2.433805000	-1.881047000	-4.074071000
6	-0.740927000	-0.623711000	-2.683575000
6	-0.914862000	-5.031866000	-3.488900000
6	-0.051571000	-6.243694000	-3.122408000
6	-2.398938000	-5.414471000	-3.400152000
1	6.179172000	-0.142483000	-3.313676000
1	5.394522000	0.259246000	-0.999218000
1	3.441861000	-0.972875000	-0.125795000
1	1.366966000	-2.163421000	-0.728186000
1	2.734538000	-5.803211000	-1.785872000
1	3.689007000	-7.289755000	1.589120000
1	4.410623000	-7.396682000	-0.016231000
1	1.199906000	-7.798551000	1.842903000
1	-0.678955000	-9.187424000	1.056170000
1	-0.575076000	-10.266834000	-1.167131000
1	1.422047000	-9.950870000	-2.595455000
1	3.289460000	-8.555931000	-1.818227000
1	-0.666484000	-2.691265000	-0.175989000
1	-3.344885000	-3.749889000	-1.205422000

1	-2.883250000	-2.058227000	-1.059974000
1	-3.047235000	-3.052328000	0.375726000
1	-1.614893000	-5.589416000	-0.418799000
1	-1.316472000	-4.690242000	1.058724000
1	0.022517000	-5.121870000	0.011967000
1	-0.326675000	-1.907336000	-4.332417000
1	-3.209840000	-1.917983000	-3.308553000
1	-2.604008000	-2.714329000	-4.756263000
1	-2.594669000	-0.964455000	-4.646237000
1	-1.380591000	-0.539611000	-1.803047000
1	-0.924110000	0.262997000	-3.294627000
1	0.295562000	-0.561179000	-2.346561000
1	-0.713266000	-4.777153000	-4.539591000
1	-0.159150000	-6.531395000	-2.076144000
1	1.011826000	-6.097122000	-3.305555000
1	-0.347557000	-7.108789000	-3.719866000
1	-2.660847000	-5.761939000	-2.399851000
1	-2.618555000	-6.234348000	-4.086881000
1	-3.075438000	-4.597033000	-3.650737000
8	3.976389000	-3.768491000	-5.730582000
1	4.732689000	-4.379229000	-5.394014000
6	5.849924000	-4.865817000	-1.575175000
6	6.238184000	-4.666059000	-0.263896000
6	6.878351000	-5.672518000	0.458834000
6	7.139682000	-6.876826000	-0.184076000
6	6.744658000	-7.094092000	-1.498597000
6	6.084440000	-6.090951000	-2.188466000
16	5.427104000	-6.413184000	-3.816087000
8	6.113662000	-7.581443000	-4.335130000
8	3.972543000	-6.580221000	-3.611722000
8	5.697278000	-5.155074000	-4.581979000
6	7.267359000	-5.444684000	1.889035000
1	5.369897000	-4.061385000	-2.115829000
1	6.031252000	-3.713449000	0.209892000
1	7.654952000	-7.666650000	0.349386000
1	6.951955000	-8.039343000	-1.982069000
1	7.694635000	-6.339875000	2.338736000
1	6.402631000	-5.149622000	2.485525000
1	8.007370000	-4.647553000	1.976300000
1	5.015140000	-1.765313000	-4.748979000

5-OH-OTs1

6	3.748138000	-4.137147000	-4.031825000
6	4.477259000	-3.079766000	-4.545542000
6	4.168837000	-1.784762000	-4.163401000
6	3.143561000	-1.544558000	-3.265125000
6	2.403332000	-2.586233000	-2.716241000
6	2.723345000	-3.874913000	-3.133109000
53	1.657095000	-5.535094000	-2.464901000
6	1.277895000	-2.247564000	-1.746468000
6	0.029511000	-1.948521000	-2.526924000
7	-0.635430000	-2.867320000	-3.257579000
7	-1.645558000	-2.318896000	-3.827623000
7	-1.657638000	-1.051662000	-3.490232000
6	-0.625970000	-0.756188000	-2.673959000
6	-2.702309000	-0.167079000	-3.969451000
6	-2.136536000	1.090148000	-4.569171000
6	-2.454196000	2.331233000	-4.035462000
6	-1.932200000	3.491525000	-4.591721000
6	-1.084008000	3.414069000	-5.683732000
6	-0.760231000	2.174415000	-6.220790000
6	-1.284899000	1.018994000	-5.668332000
8	1.577221000	-1.140245000	-0.977398000
14	2.169409000	-0.942958000	0.603441000
6	2.528328000	-2.643227000	1.354619000
6	3.420907000	-2.566570000	2.598073000
6	1.280404000	-3.471034000	1.674066000
6	3.726053000	0.114610000	0.365444000
6	4.140243000	0.937328000	1.586572000
6	4.909280000	-0.696056000	-0.169022000
6	0.825334000	0.046367000	1.519399000
6	-0.598769000	-0.408619000	1.193308000
6	1.024420000	0.064123000	3.038081000
1	5.289113000	-3.275150000	-5.232191000
1	4.735451000	-0.953125000	-4.560659000
1	2.917592000	-0.528843000	-2.968183000
1	1.082446000	-3.124766000	-1.118406000
1	-0.436756000	0.231311000	-2.291566000
1	-3.377388000	0.068005000	-3.144177000
1	-3.269499000	-0.742046000	-4.701667000
1	-3.115349000	2.396015000	-3.179175000
1	-2.187548000	4.453668000	-4.168183000
1	-0.674387000	4.316345000	-6.117865000
1	-0.099386000	2.109569000	-7.074882000
1	-1.028464000	0.054568000	-6.091671000
1	3.098072000	-3.181903000	0.588732000

1	2.945567000	-2.025606000	3.417811000
1	4.377101000	-2.083549000	2.397070000
1	3.640196000	-3.572681000	2.964681000
1	0.720695000	-3.048881000	2.511165000
1	1.564151000	-4.488487000	1.950305000
1	0.584819000	-3.555488000	0.837326000
1	3.433765000	0.828658000	-0.417256000
1	4.408826000	0.306790000	2.436353000
1	3.353868000	1.617432000	1.916983000
1	5.016870000	1.548502000	1.357616000
1	5.304140000	-1.384078000	0.579163000
1	5.730932000	-0.034875000	-0.456529000
1	4.659478000	-1.300859000	-1.039826000
1	0.933010000	1.079007000	1.159684000
1	-0.784298000	-1.430471000	1.528437000
1	-0.815790000	-0.374660000	0.126315000
1	-1.331317000	0.228311000	1.696523000
1	0.860885000	-0.923840000	3.471893000
1	0.309976000	0.740671000	3.513814000
1	2.021237000	0.388391000	3.337100000
8	0.401568000	-5.440516000	-3.939864000
1	-0.151435000	-4.641176000	-3.854694000
6	5.536804000	-5.610426000	1.697335000
6	5.858284000	-6.221874000	2.900097000
6	5.747770000	-7.597990000	3.060407000
6	5.309485000	-8.357065000	1.976159000
6	4.983324000	-7.763049000	0.771062000
6	5.096658000	-6.385907000	0.638652000
16	4.638437000	-5.609989000	-0.888259000
8	3.113195000	-5.504042000	-0.743663000
8	5.241769000	-4.288429000	-0.916416000
8	4.961046000	-6.516919000	-1.982573000
6	6.088059000	-8.260260000	4.362512000
1	5.642351000	-4.540204000	1.583754000
1	6.208195000	-5.613457000	3.725135000
1	5.227114000	-9.432842000	2.075451000
1	4.657266000	-8.368216000	-0.064165000
1	6.808521000	-9.066736000	4.221655000
1	5.201547000	-8.697336000	4.825074000
1	6.515752000	-7.555002000	5.073491000
1	3.996283000	-5.151082000	-4.311948000

5-OH-OTs2

6	2.592119000	-7.467725000	-0.999061000
6	3.508168000	-6.989001000	-1.921165000
6	3.673364000	-5.625315000	-2.065072000
6	2.963140000	-4.743757000	-1.263831000
6	2.046773000	-5.187834000	-0.316844000
6	1.857440000	-6.570909000	-0.236830000
53	0.389584000	-7.471370000	0.950315000
6	1.394003000	-4.177773000	0.623394000
6	-0.093858000	-4.320244000	0.654982000
7	-0.769539000	-4.496935000	1.807573000
7	-2.021032000	-4.618664000	1.553003000
7	-2.182079000	-4.518611000	0.251223000
6	-1.005739000	-4.327757000	-0.368990000
6	-3.522085000	-4.580194000	-0.323684000
6	-3.478351000	-4.843613000	-1.799947000
6	-3.580838000	-3.790265000	-2.702242000
6	-3.503463000	-4.025714000	-4.067137000
6	-3.322812000	-5.318534000	-4.536444000
6	-3.224100000	-6.375490000	-3.641900000
6	-3.303160000	-6.138428000	-2.278170000
8	1.755024000	-2.888390000	0.274473000
14	2.311249000	-1.556772000	1.159061000
6	2.666523000	-2.101372000	2.943910000
6	3.580356000	-1.124103000	3.691342000
6	1.407508000	-2.367902000	3.774305000
6	3.887254000	-1.062556000	0.221087000
6	4.345158000	0.380863000	0.434324000
6	5.038626000	-2.042484000	0.460287000
6	0.995321000	-0.197840000	0.991490000
6	-0.438607000	-0.731101000	1.023907000
6	1.164128000	0.932381000	2.011337000
1	4.067738000	-7.686003000	-2.528469000
1	4.369790000	-5.236077000	-2.795926000
1	3.121672000	-3.680936000	-1.371787000
1	1.755852000	-4.422461000	1.632649000
1	-0.905295000	-4.236110000	-1.436251000
1	-4.027054000	-3.636382000	-0.110362000
1	-4.056709000	-5.367219000	0.207313000
1	-3.724012000	-2.779786000	-2.336518000
1	-3.588181000	-3.200545000	-4.761925000
1	-3.261933000	-5.504982000	-5.600578000
1	-3.077506000	-7.387330000	-3.993989000
1	-3.213507000	-6.969724000	-1.590417000
1	3.218964000	-3.047964000	2.857104000
1	3.117633000	-0.143992000	3.814231000

1	4.537631000	-0.973804000	3.192908000
1	3.798109000	-1.500005000	4.693898000
1	0.846238000	-1.449012000	3.952517000
1	1.675104000	-2.769137000	4.755197000
1	0.719392000	-3.076350000	3.313640000
1	3.594240000	-1.149956000	-0.834742000
1	4.627922000	0.576837000	1.470026000
1	3.573426000	1.102365000	0.163333000
1	5.220864000	0.603169000	-0.180557000
1	5.425286000	-1.972747000	1.478311000
1	5.875967000	-1.832422000	-0.210004000
1	4.749476000	-3.081778000	0.296697000
1	1.160889000	0.232936000	-0.005542000
1	-0.673052000	-1.228467000	1.966777000
1	-0.627614000	-1.446170000	0.224018000
1	-1.153033000	0.088244000	0.907376000
1	0.948605000	0.593008000	3.025674000
1	0.470932000	1.749574000	1.797972000
1	2.168301000	1.357932000	2.017243000
8	1.193890000	-6.872675000	2.632671000
1	0.716396000	-6.113241000	2.986436000
6	1.445363000	-9.006548000	-3.997239000
6	2.208646000	-8.412775000	-4.991889000
6	2.067136000	-7.064921000	-5.300667000
6	1.125701000	-6.320797000	-4.591742000
6	0.353085000	-6.898609000	-3.602202000
6	0.524553000	-8.242901000	-3.299882000
16	-0.453619000	-9.008983000	-2.027366000
8	-0.616816000	-7.885446000	-0.995938000
8	-1.749375000	-9.333727000	-2.592197000
8	0.336754000	-10.097344000	-1.469895000
6	2.894637000	-6.419554000	-6.372427000
1	1.580975000	-10.053259000	-3.759605000
1	2.930133000	-9.013063000	-5.532867000
1	0.998016000	-5.267991000	-4.813795000
1	-0.369220000	-6.302179000	-3.062259000
1	3.389181000	-5.519160000	-6.005454000
1	2.276617000	-6.122696000	-7.221345000
1	3.664656000	-7.091816000	-6.747928000
1	2.451864000	-8.535319000	-0.893717000

6c-OH-N

6	3.529392000	0.606805000	0.735337000
6	2.541652000	1.228717000	1.499476000
6	1.259918000	0.710390000	1.515375000
6	0.945719000	-0.428278000	0.792598000
6	1.905764000	-1.046753000	0.002896000
6	3.186450000	-0.500152000	-0.045533000
53	4.654614000	-1.335768000	-1.265602000
8	4.803790000	0.983202000	0.723060000
6	5.194394000	2.192617000	1.313481000
6	1.521071000	-2.309330000	-0.750491000
6	2.383754000	-3.459233000	-0.313060000
7	3.705366000	-3.291212000	-0.107519000
7	4.275944000	-4.405409000	0.146767000
7	3.348887000	-5.324855000	0.120434000
6	2.139694000	-4.795918000	-0.165763000
6	3.697018000	-6.726817000	0.276657000
6	3.428205000	-7.498110000	-0.987147000
6	2.586070000	-8.601696000	-0.969194000
6	2.341000000	-9.316137000	-2.133871000
6	2.934908000	-8.922355000	-3.322037000
6	3.774568000	-7.816398000	-3.342454000
6	4.025727000	-7.104280000	-2.181863000
8	1.663329000	-2.138616000	-2.112526000
14	0.634495000	-2.106541000	-3.448788000
6	-1.140601000	-2.324834000	-2.793719000
6	-2.210898000	-1.896370000	-3.801022000
6	-1.426943000	-3.746040000	-2.300493000
6	0.944904000	-0.396607000	-4.198613000
6	0.472969000	-0.227904000	-5.642776000
6	0.423303000	0.727740000	-3.302630000
6	1.218577000	-3.473897000	-4.618136000
6	1.728541000	-4.723320000	-3.899822000
6	0.191325000	-3.847559000	-5.689276000
1	2.769691000	2.102527000	2.094197000
1	0.499728000	1.196466000	2.112116000
1	-0.055910000	-0.837254000	0.834939000
1	4.647153000	3.041591000	0.894081000
1	6.250969000	2.317224000	1.093546000
1	5.063124000	2.180070000	2.399861000
1	0.485310000	-2.544405000	-0.471761000
1	1.251101000	-5.394507000	-0.270682000
1	3.137292000	-7.138740000	1.117878000
1	4.754246000	-6.742075000	0.540828000
1	2.119710000	-8.911392000	-0.040742000
1	1.685625000	-10.176675000	-2.110752000

1	2.744579000	-9.475823000	-4.232124000
1	4.241431000	-7.502984000	-4.266718000
1	4.677312000	-6.237831000	-2.219472000
1	-1.224558000	-1.645356000	-1.933247000
1	-2.178361000	-2.495908000	-4.711913000
1	-2.108294000	-0.851492000	-4.093489000
1	-3.211520000	-2.014056000	-3.376933000
1	-1.410565000	-4.464725000	-3.120784000
1	-2.415797000	-3.808714000	-1.838677000
1	-0.704754000	-4.097753000	-1.560715000
1	2.041281000	-0.325529000	-4.207613000
1	-0.610509000	-0.316630000	-5.741807000
1	0.926556000	-0.963869000	-6.308179000
1	0.745732000	0.759704000	-6.023881000
1	-0.667213000	0.726237000	-3.236795000
1	0.716773000	1.705052000	-3.693600000
1	0.817073000	0.661290000	-2.287135000
1	2.079799000	-3.033438000	-5.134024000
1	0.953117000	-5.210186000	-3.302886000
1	2.573603000	-4.503886000	-3.249703000
1	2.075307000	-5.461716000	-4.627214000
1	-0.672769000	-4.362963000	-5.264761000
1	0.638028000	-4.528239000	-6.418829000
1	-0.184524000	-2.985521000	-6.242895000
8	4.919313000	0.422687000	-2.073669000
1	5.570522000	0.331702000	-2.777308000
6	4.609804000	-1.699738000	-5.689133000
6	3.808281000	-1.613885000	-6.820946000
6	3.442502000	-2.748640000	-7.532480000
6	3.913608000	-3.983633000	-7.085819000
6	4.716472000	-4.080353000	-5.964704000
6	5.061742000	-2.934101000	-5.257335000
16	6.095311000	-3.109315000	-3.808819000
8	7.317814000	-3.754334000	-4.252715000
8	6.292476000	-1.714937000	-3.303912000
8	5.292285000	-3.886240000	-2.844926000
6	2.548835000	-2.660702000	-8.734108000
1	4.886015000	-0.800157000	-5.157686000
1	3.465855000	-0.640689000	-7.152241000
1	3.651218000	-4.883602000	-7.629884000
1	5.081563000	-5.046882000	-5.642170000
1	2.957761000	-3.215974000	-9.578815000
1	2.406454000	-1.629349000	-9.054215000
1	1.562148000	-3.077356000	-8.521151000

6c-OH-O

6	3.589278000	-0.440289000	-3.432197000
6	3.503674000	0.844795000	-2.898054000
6	2.793875000	1.058158000	-1.728082000
6	2.130416000	0.021244000	-1.092453000
6	2.224688000	-1.266072000	-1.596629000
6	2.996285000	-1.491233000	-2.728964000
53	3.282898000	-3.455322000	-3.345209000
8	4.170569000	-0.737083000	-4.589171000
6	5.002763000	0.196542000	-5.225502000
6	1.429266000	-2.399727000	-0.979176000
6	2.234849000	-3.211876000	-0.010711000
7	2.800502000	-2.681066000	1.089256000
7	3.413604000	-3.605053000	1.732394000
7	3.255011000	-4.736670000	1.076033000
6	2.516919000	-4.551797000	-0.032058000
6	3.908803000	-5.960239000	1.515836000
6	3.109935000	-7.167363000	1.115911000
6	2.044041000	-7.599175000	1.898772000
6	1.269236000	-8.674382000	1.494421000
6	1.552108000	-9.325292000	0.300831000
6	2.614087000	-8.900983000	-0.482770000
6	3.391819000	-7.827028000	-0.074783000
8	0.963680000	-3.239601000	-2.000684000
14	-0.619101000	-3.450043000	-2.620041000
6	-1.802130000	-3.168563000	-1.164908000
6	-3.274013000	-3.080115000	-1.578115000
6	-1.612580000	-4.197618000	-0.047283000
6	-0.817573000	-2.148949000	-3.985657000
6	-1.908764000	-2.475432000	-5.007477000
6	-0.996314000	-0.735055000	-3.428518000
6	-0.614185000	-5.199116000	-3.350726000
6	0.240244000	-6.213785000	-2.586003000
6	-2.036251000	-5.744417000	-3.528866000
1	3.970698000	1.682675000	-3.397331000
1	2.733280000	2.060026000	-1.324819000
1	1.543806000	0.207624000	-0.202547000
1	4.441857000	1.061700000	-5.591497000
1	5.448671000	-0.313974000	-6.074439000
1	5.802239000	0.538630000	-4.562778000
1	0.595643000	-1.945303000	-0.427591000
1	2.260996000	-5.347913000	-0.708193000
1	4.020150000	-5.884473000	2.596952000
1	4.908264000	-6.000893000	1.077396000
1	1.816489000	-7.088115000	2.827117000
1	0.444042000	-9.005801000	2.110849000

1	0.945187000	-10.163342000	-0.015613000
1	2.841245000	-9.397817000	-1.416455000
1	4.215002000	-7.496322000	-0.696222000
1	-1.537379000	-2.182824000	-0.757775000
1	-3.643902000	-4.019803000	-1.988119000
1	-3.452990000	-2.304476000	-2.323362000
1	-3.897811000	-2.842147000	-0.713403000
1	-1.900908000	-5.199048000	-0.371563000
1	-2.235396000	-3.949929000	0.815522000
1	-0.581875000	-4.256875000	0.306912000
1	0.140696000	-2.170340000	-4.524009000
1	-2.903085000	-2.500260000	-4.557717000
1	-1.744548000	-3.436744000	-5.495489000
1	-1.935903000	-1.718266000	-5.794537000
1	-1.932912000	-0.630722000	-2.877068000
1	-1.020380000	-0.000750000	-4.237138000
1	-0.185024000	-0.440215000	-2.761083000
1	-0.177972000	-5.095741000	-4.353648000
1	-0.044011000	-6.292452000	-1.534951000
1	1.303966000	-5.986972000	-2.633033000
1	0.125698000	-7.207845000	-3.023904000
1	-2.502439000	-5.963933000	-2.566730000
1	-2.014701000	-6.680032000	-4.091466000
1	-2.697822000	-5.063188000	-4.065540000
8	5.148165000	-3.186977000	-3.571384000
1	5.564139000	-3.915972000	-4.127562000
6	6.491464000	-6.351937000	-1.956848000
6	7.027531000	-6.923484000	-0.813879000
6	7.023272000	-8.303708000	-0.626006000
6	6.482603000	-9.100877000	-1.630082000
6	5.946790000	-8.544143000	-2.781442000
6	5.944613000	-7.166710000	-2.938941000
16	5.177739000	-6.436505000	-4.372914000
8	5.032560000	-7.478237000	-5.367836000
8	3.878282000	-5.902637000	-3.854533000
8	6.041262000	-5.293665000	-4.764028000
6	7.557592000	-8.914306000	0.635033000
1	6.517001000	-5.276789000	-2.078177000
1	7.467422000	-6.281774000	-0.059158000
1	6.481336000	-10.178024000	-1.514160000
1	5.540900000	-9.179010000	-3.557373000
1	6.746802000	-9.125797000	1.335482000
1	8.260499000	-8.251433000	1.138064000
1	8.069383000	-9.856243000	0.439910000

6c-OH-OTs1

6	4.312225000	-3.017881000	-2.954328000
6	4.697980000	-1.684588000	-3.032564000
6	3.993672000	-0.727751000	-2.320400000
6	2.930687000	-1.071793000	-1.507041000
6	2.547707000	-2.403272000	-1.377926000
6	3.226887000	-3.350682000	-2.132401000
53	2.710846000	-5.358061000	-2.114460000
8	4.899613000	-4.009087000	-3.609632000
6	6.176623000	-3.829674000	-4.173757000
6	1.427384000	-2.742421000	-0.401700000
6	0.094383000	-2.567798000	-1.066775000
7	-0.309781000	-3.307577000	-2.120475000
7	-1.484029000	-2.940629000	-2.482682000
7	-1.863057000	-1.968562000	-1.687826000
6	-0.911114000	-1.687422000	-0.775017000
6	-3.170053000	-1.357304000	-1.835664000
6	-3.083499000	0.143250000	-1.864854000
6	-3.714122000	0.905392000	-0.891430000
6	-3.632806000	2.291311000	-0.919751000
6	-2.914395000	2.921544000	-1.922048000
6	-2.278862000	2.163976000	-2.897871000
6	-2.364268000	0.782880000	-2.870851000
8	1.460581000	-1.934707000	0.720692000
14	2.306564000	-2.129822000	2.184339000
6	2.492804000	-3.990655000	2.486890000
6	3.415075000	-4.345143000	3.657422000
6	1.147069000	-4.705947000	2.634269000
6	3.981100000	-1.250393000	2.011909000
6	4.559399000	-0.760734000	3.342231000
6	5.021563000	-2.077830000	1.255447000
6	1.217150000	-1.211549000	3.446486000
6	-0.281496000	-1.300538000	3.155327000
6	1.493419000	-1.648832000	4.888258000
1	5.538778000	-1.389272000	-3.644653000
1	4.292617000	0.309321000	-2.397170000
1	2.410822000	-0.311146000	-0.941235000
1	6.142889000	-3.160917000	-5.038892000
1	6.506300000	-4.811675000	-4.500137000
1	6.884215000	-3.453096000	-3.433124000
1	1.518648000	-3.799104000	-0.119738000
1	-1.012406000	-0.916355000	-0.032439000
1	-3.814611000	-1.691250000	-1.020111000
1	-3.583894000	-1.754510000	-2.762655000
1	-4.275123000	0.415916000	-0.103878000
1	-4.129264000	2.875377000	-0.156400000

1	-2.848232000	4.001103000	-1.945660000
1	-1.718315000	2.652523000	-3.683692000
1	-1.866096000	0.196692000	-3.634571000
1	2.985904000	-4.381088000	1.587583000
1	3.002951000	-4.034458000	4.618412000
1	4.404496000	-3.898775000	3.560468000
1	3.568365000	-5.425962000	3.702507000
1	0.636588000	-4.417301000	3.555383000
1	1.288647000	-5.788708000	2.673294000
1	0.459058000	-4.502316000	1.810782000
1	3.758894000	-0.359275000	1.409146000
1	4.794343000	-1.587786000	4.015411000
1	3.884818000	-0.086591000	3.872019000
1	5.491921000	-0.215773000	3.175548000
1	5.315745000	-2.964062000	1.818589000
1	5.929070000	-1.494280000	1.081487000
1	4.679048000	-2.423825000	0.282180000
1	1.503906000	-0.154675000	3.363448000
1	-0.638466000	-2.331900000	3.151645000
1	-0.536967000	-0.863405000	2.191660000
1	-0.852865000	-0.764848000	3.918260000
1	1.158897000	-2.672576000	5.064618000
1	0.954347000	-1.012314000	5.594288000
1	2.549438000	-1.602325000	5.155333000
8	1.381873000	-5.132027000	-3.504495000
1	0.645738000	-4.574269000	-3.185836000
6	5.530184000	-7.231141000	1.739556000
6	5.790141000	-7.507073000	3.068301000
6	6.612487000	-6.676935000	3.830532000
6	7.176818000	-5.566348000	3.216654000
6	6.923957000	-5.274390000	1.883085000
6	6.095423000	-6.106284000	1.151949000
16	5.720118000	-5.734293000	-0.544085000
8	4.234675000	-5.377074000	-0.438435000
8	6.516932000	-4.579217000	-0.930163000
8	5.884576000	-6.946064000	-1.331927000
6	6.863784000	-6.978816000	5.278028000
1	4.897826000	-7.891718000	1.161221000
1	5.350418000	-8.386914000	3.522965000
1	7.828995000	-4.914544000	3.785192000
1	7.374004000	-4.408487000	1.417352000
1	7.658829000	-6.357028000	5.686939000
1	7.148455000	-8.021021000	5.424998000
1	5.967925000	-6.801429000	5.876022000

6c-OH-OTs2

6	3.926231000	-3.838247000	-3.400867000
6	3.891285000	-2.626809000	-4.071403000
6	3.086193000	-1.606390000	-3.586151000
6	2.320126000	-1.773012000	-2.452487000
6	2.328717000	-2.983217000	-1.758368000
6	3.139310000	-3.998426000	-2.250834000
53	3.326874000	-5.850484000	-1.321562000
8	4.692956000	-4.875699000	-3.766400000
6	5.427590000	-4.811678000	-4.961759000
6	1.451077000	-3.084275000	-0.518601000
6	0.003367000	-3.150723000	-0.901893000
7	-0.640100000	-2.151166000	-1.540797000
7	-1.855861000	-2.496133000	-1.747924000
7	-2.029282000	-3.707429000	-1.251213000
6	-0.893543000	-4.166894000	-0.703176000
6	-3.282327000	-4.418745000	-1.430613000
6	-3.659041000	-5.183752000	-0.193853000
6	-3.478389000	-6.560084000	-0.138117000
6	-3.800655000	-7.261465000	1.015891000
6	-4.302501000	-6.590791000	2.119814000
6	-4.481979000	-5.214525000	2.070139000
6	-4.161925000	-4.515430000	0.918473000
8	1.691227000	-1.984612000	0.288120000
14	2.265930000	-1.813556000	1.861101000
6	2.388656000	-3.550341000	2.625360000
6	3.231875000	-3.600529000	3.902120000
6	1.019546000	-4.191657000	2.869175000
6	3.958601000	-0.983499000	1.660520000
6	4.509639000	-0.312456000	2.918908000
6	4.985245000	-1.934171000	1.041984000
6	1.040513000	-0.650911000	2.726358000
6	-0.409334000	-0.877159000	2.290008000
6	1.149318000	-0.679708000	4.253406000
1	4.481030000	-2.466064000	-4.963114000
1	3.062096000	-0.660552000	-4.111101000
1	1.694173000	-0.968843000	-2.094039000
1	4.779410000	-4.620839000	-5.820569000
1	5.899795000	-5.782249000	-5.089597000
1	6.206057000	-4.044291000	-4.917721000
1	1.675909000	-4.018298000	0.008480000
1	-0.809807000	-5.142775000	-0.259003000
1	-3.180693000	-5.092352000	-2.283055000
1	-4.028176000	-3.664878000	-1.681524000
1	-3.072258000	-7.082223000	-0.996333000
1	-3.655672000	-8.333111000	1.049919000

1	-4.554656000	-7.137674000	3.018865000
1	-4.874682000	-4.687132000	2.929518000
1	-4.299679000	-3.440789000	0.882433000
1	2.918432000	-4.161312000	1.879964000
1	2.807637000	-2.988931000	4.699555000
1	4.254561000	-3.261600000	3.736883000
1	3.292004000	-4.623471000	4.282133000
1	0.479773000	-3.683564000	3.669976000
1	1.126572000	-5.236748000	3.170280000
1	0.370415000	-4.173316000	1.991973000
1	3.766678000	-0.188288000	0.927531000
1	4.700730000	-1.026888000	3.721529000
1	3.831278000	0.446438000	3.311376000
1	5.458716000	0.186650000	2.706930000
1	5.232124000	-2.763457000	1.707786000
1	5.920822000	-1.411677000	0.825865000
1	4.639018000	-2.367968000	0.102422000
1	1.328155000	0.356276000	2.395078000
1	-0.769264000	-1.868442000	2.572859000
1	-0.538584000	-0.779262000	1.213058000
1	-1.069439000	-0.149579000	2.769854000
1	0.816803000	-1.636264000	4.660778000
1	0.515518000	0.090377000	4.699886000
1	2.164522000	-0.508433000	4.614126000
8	5.183748000	-5.471253000	-0.794406000
1	5.818512000	-5.539983000	-1.509553000
6	1.804896000	-6.142653000	-5.331161000
6	2.121558000	-5.281904000	-6.374588000
6	1.620788000	-3.987158000	-6.418306000
6	0.770514000	-3.569648000	-5.394300000
6	0.432299000	-4.417220000	-4.359453000
6	0.961772000	-5.702371000	-4.326917000
16	0.554732000	-6.771233000	-2.970066000
8	1.190619000	-6.057367000	-1.759276000
8	-0.885573000	-6.752435000	-2.791647000
8	1.209486000	-8.046718000	-3.196367000
6	1.977389000	-3.049203000	-7.532224000
1	2.204642000	-7.147144000	-5.308997000
1	2.764182000	-5.635181000	-7.172696000
1	0.365488000	-2.565216000	-5.402516000
1	-0.236415000	-4.071571000	-3.583994000
1	2.444828000	-2.141155000	-7.146946000
1	1.090953000	-2.739858000	-8.087483000
1	2.666747000	-3.504914000	-8.241897000

6c-OH⁺

6	3.763705000	-3.360946000	-3.846407000
6	3.880447000	-1.987375000	-4.032575000
6	3.422773000	-1.125211000	-3.052099000
6	2.836628000	-1.595598000	-1.888325000
6	2.722651000	-2.959139000	-1.667863000
6	3.207118000	-3.820448000	-2.649198000
53	3.109080000	-5.890846000	-2.431878000
8	4.146605000	-4.277638000	-4.738447000
6	4.590479000	-3.875279000	-6.019625000
6	2.014142000	-3.485678000	-0.425682000
6	0.815853000	-4.268065000	-0.877092000
7	0.925460000	-5.272698000	-1.762676000
7	-0.214816000	-5.821458000	-1.990876000
7	-1.083633000	-5.179493000	-1.264912000
6	-0.507718000	-4.191041000	-0.546565000
6	-2.505661000	-5.536490000	-1.310086000
6	-3.183402000	-5.151370000	-0.029011000
6	-2.978823000	-5.905358000	1.123212000
6	-3.557905000	-5.520162000	2.320544000
6	-4.350148000	-4.380635000	2.374417000
6	-4.564531000	-3.630833000	1.228718000
6	-3.981081000	-4.015469000	0.029625000
8	1.608065000	-2.492991000	0.421027000
14	2.084375000	-2.148189000	2.030300000
6	2.332634000	-3.823264000	2.887747000
6	3.016070000	-3.709633000	4.254196000
6	1.037817000	-4.632453000	3.002368000
6	3.690398000	-1.154202000	1.895479000
6	4.037088000	-0.347559000	3.149273000
6	4.873270000	-2.019650000	1.453641000
6	0.665690000	-1.085559000	2.696117000
6	-0.712826000	-1.461216000	2.147079000
6	0.633467000	-1.050727000	4.227974000
1	4.319636000	-1.584676000	-4.934482000
1	3.515488000	-0.058969000	-3.207575000
1	2.457114000	-0.904427000	-1.150598000
1	3.832986000	-3.281504000	-6.535608000
1	4.766759000	-4.784916000	-6.586303000
1	5.523127000	-3.310029000	-5.960971000
1	2.704729000	-4.193569000	0.069800000
1	-1.050221000	-3.546484000	0.123050000
1	-2.550271000	-6.609433000	-1.494427000
1	-2.955944000	-5.031684000	-2.165909000
1	-2.367178000	-6.799631000	1.084830000
1	-3.400160000	-6.113564000	3.210940000

1	-4.807138000	-4.083778000	3.308711000
1	-5.189391000	-2.748894000	1.264992000
1	-4.153816000	-3.428359000	-0.864840000
1	3.022380000	-4.390856000	2.246298000
1	2.415558000	-3.148644000	4.970386000
1	3.990987000	-3.226310000	4.194073000
1	3.178894000	-4.700892000	4.682477000
1	0.316851000	-4.147457000	3.662273000
1	1.236661000	-5.621205000	3.422021000
1	0.543022000	-4.786087000	2.041471000
1	3.495355000	-0.427115000	1.095134000
1	4.226254000	-0.983893000	4.014840000
1	3.245973000	0.351602000	3.422310000
1	4.941425000	0.242706000	2.987182000
1	5.127964000	-2.774152000	2.200072000
1	5.766475000	-1.409667000	1.303085000
1	4.689233000	-2.538904000	0.510279000
1	0.887362000	-0.066963000	2.350853000
1	-0.997629000	-2.481929000	2.413262000
1	-0.762560000	-1.365095000	1.062543000
1	-1.479140000	-0.803337000	2.563113000
1	0.347725000	-2.017105000	4.646617000
1	-0.102149000	-0.323908000	4.577992000
1	1.590471000	-0.773419000	4.671022000
8	5.002279000	-6.186561000	-2.702832000
1	5.308535000	-6.110958000	-3.610079000

6c-OH-N$^+$

6	3.563345000	0.516749000	0.906378000
6	2.565043000	0.957813000	1.777135000
6	1.307585000	0.388417000	1.725577000
6	1.018106000	-0.633607000	0.832907000
6	1.979306000	-1.076138000	-0.061275000
6	3.228220000	-0.462142000	-0.034737000
53	4.740027000	-0.986967000	-1.365849000
8	4.815606000	0.941484000	0.920201000
6	5.209368000	2.010439000	1.751880000
6	1.667257000	-2.242525000	-0.978043000
6	2.624640000	-3.362102000	-0.682479000
7	3.952619000	-3.134426000	-0.701732000
7	4.615159000	-4.217865000	-0.527402000
7	3.740833000	-5.171790000	-0.380522000
6	2.476884000	-4.703349000	-0.463424000
6	4.168078000	-6.552075000	-0.146993000
6	3.270289000	-7.515165000	-0.867495000
6	2.432063000	-8.362434000	-0.155207000
6	1.588841000	-9.238944000	-0.825058000
6	1.577855000	-9.265935000	-2.209995000
6	2.414635000	-8.420154000	-2.927539000
6	3.259745000	-7.551035000	-2.259748000
8	1.800837000	-1.873975000	-2.301517000
14	0.757775000	-1.953980000	-3.649756000
6	-0.899118000	-2.653283000	-3.042213000
6	-2.051589000	-2.382991000	-4.015439000
6	-0.844083000	-4.145327000	-2.700603000
6	0.657933000	-0.149574000	-4.206473000
6	0.141235000	0.059976000	-5.630914000
6	-0.109808000	0.711247000	-3.200631000
6	1.654464000	-3.021604000	-4.935317000
6	2.429373000	-4.195801000	-4.331568000
6	0.725998000	-3.513747000	-6.050582000
1	2.772007000	1.730470000	2.504508000
1	0.543448000	0.736593000	2.407029000
1	0.037071000	-1.090601000	0.832101000
1	4.635341000	2.914837000	1.537707000
1	6.255394000	2.199782000	1.531875000
1	5.110637000	1.754480000	2.809565000
1	0.654760000	-2.587981000	-0.736149000
1	1.612555000	-5.340808000	-0.384867000
1	4.167000000	-6.736868000	0.928293000
1	5.199819000	-6.611945000	-0.492636000
1	2.441074000	-8.348256000	0.928445000
1	0.944808000	-9.901466000	-0.263014000

1	0.923347000	-9.950158000	-2.732781000
1	2.415096000	-8.447314000	-4.008836000
1	3.916965000	-6.900327000	-2.825902000
1	-1.134381000	-2.103030000	-2.119579000
1	-1.893907000	-2.861218000	-4.982685000
1	-2.203275000	-1.319290000	-4.195934000
1	-2.989279000	-2.774861000	-3.615663000
1	-0.693524000	-4.755249000	-3.591920000
1	-1.780777000	-4.474239000	-2.245299000
1	-0.041766000	-4.402798000	-2.005912000
1	1.705289000	0.184945000	-4.194208000
1	-0.888609000	-0.277792000	-5.753896000
1	0.748324000	-0.461737000	-6.371575000
1	0.159476000	1.119603000	-5.894321000
1	-1.168339000	0.447116000	-3.164113000
1	-0.059170000	1.767739000	-3.472964000
1	0.286441000	0.622620000	-2.186992000
1	2.389719000	-2.348731000	-5.397109000
1	1.773655000	-4.904209000	-3.820803000
1	3.184430000	-3.868930000	-3.615480000
1	2.948724000	-4.752126000	-5.115822000
1	0.003250000	-4.243684000	-5.682641000
1	1.299210000	-4.006115000	-6.838771000
1	0.162632000	-2.708753000	-6.523259000
8	5.129227000	0.836539000	-1.905205000
1	4.577314000	1.162536000	-2.619873000

OTs$^-$

6	1.806112000	-6.129176000	-5.319835000
6	2.118383000	-5.274776000	-6.371868000
6	1.622374000	-3.979696000	-6.418891000
6	0.797908000	-3.556086000	-5.376432000
6	0.482738000	-4.399295000	-4.327024000
6	0.987122000	-5.695268000	-4.293128000
16	0.557728000	-6.769074000	-2.907937000
8	1.075919000	-6.055802000	-1.731629000
8	-0.909690000	-6.841992000	-2.956047000
8	1.236194000	-8.041544000	-3.190962000
6	1.955342000	-3.054125000	-7.554436000
1	2.198990000	-7.136568000	-5.294801000
1	2.761977000	-5.628654000	-7.170142000
1	0.396926000	-2.547953000	-5.388789000
1	-0.158558000	-4.055104000	-3.526048000
1	2.447661000	-2.145593000	-7.201443000
1	1.059100000	-2.743254000	-8.095506000
1	2.622678000	-3.528990000	-8.273915000

(*R*)-11

6	2.823075000	-2.529821000	-1.629873000
6	1.774877000	-3.327153000	-2.385011000
6	0.470154000	-2.726390000	-1.925685000
8	-0.628266000	-2.863219000	-2.389284000
8	0.683154000	-1.938549000	-0.850649000
1	3.718748000	-3.098884000	-1.348250000
1	3.140984000	-1.648779000	-2.205880000
1	1.766418000	-4.393332000	-2.112214000
1	1.853623000	-3.259774000	-3.478218000
6	3.677256000	-1.765171000	1.985699000
6	3.080713000	-3.022457000	1.739330000
6	3.315481000	-0.610369000	1.167156000
6	2.513802000	-0.717736000	0.098812000
6	2.028812000	-2.040978000	-0.385966000
6	2.088438000	-3.161217000	0.658581000
1	2.247443000	0.148214000	-0.513455000
8	1.366500000	-4.130752000	0.533818000
6	4.580122000	-1.649614000	3.049348000
1	5.039635000	-0.680281000	3.257798000
1	3.722748000	0.362065000	1.455159000
6	4.890067000	-2.755611000	3.838474000
1	5.602431000	-2.649106000	4.660235000
6	4.293457000	-3.995527000	3.590908000
1	4.541308000	-4.858574000	4.212585000
6	3.381336000	-4.122426000	2.548402000
1	2.892324000	-5.076123000	2.338079000

8. TD-DFT/TDA Excited states for (*R*)-11

ABSORPTION SPECTRUM VIA TRANSITION ELECTRIC DIPOLE MOMENTS

State	Energy (cm-1)	hv (nm)	fosc	T2 (au**2)	TX (au)	TY (au)	TZ (au)
1	28367.8	352.5	0.029933811	0.34739	-0.02629	0.44412	-0.38658
2	31599.1	316.5	0.031820853	0.33152	-0.02652	-0.53982	0.19854
3	37298.1	268.1	0.021916840	0.19345	-0.26923	-0.30766	-0.16221
4	35115.4	284.8	0.005214042	0.04888	0.16350	0.14308	0.04099
5	42443.2	235.6	0.127642645	0.99006	-0.61284	0.04921	-0.78235
6	44919.2	222.6	0.018444338	0.13518	-0.08610	0.32735	-0.14355
7	46772.0	213.8	0.547106589	3.85090	0.97822	-0.63731	1.57728
8	46608.7	214.6	0.223643206	1.57966	0.65442	-0.29523	1.03162
9	46789.5	213.7	0.014531679	0.10225	-0.17907	-0.03003	-0.26320
10	48771.0	205.0	0.128668423	0.86853	-0.55133	0.01194	-0.75128
11	49775.6	200.9	0.045486814	0.30085	0.32124	0.14772	0.41932
12	50366.2	198.5	0.143671962	0.93909	-0.55764	-0.05610	-0.79056
13	52406.9	190.8	0.060312598	0.37887	0.36426	-0.11334	0.48306
14	46861.0	213.4	0.007130007	0.05009	-0.12606	0.03466	-0.18166
15	52859.3	189.2	0.032481917	0.20230	-0.07871	0.34338	-0.27963
16	53206.4	187.9	0.034806068	0.21536	-0.18226	0.07105	-0.42082
17	52444.5	190.7	0.001437874	0.00903	0.08195	0.04524	-0.01623
18	55125.1	181.4	0.090325635	0.53943	-0.45063	-0.26524	-0.51576
19	55378.6	180.6	0.011907440	0.07079	0.06125	-0.20030	-0.16405
20	55704.6	179.5	0.009251120	0.05467	0.12776	0.03056	0.19344
21	56032.8	178.5	0.039782095	0.23373	-0.00757	-0.30090	0.37833
22	57350.3	174.4	0.000344061	0.00198	-0.03101	0.01167	-0.02962
23	58507.1	170.9	0.009453517	0.05319	-0.06053	0.22143	-0.02230
24	57055.1	175.3	0.003718630	0.02146	0.01721	-0.07978	-0.12163
25	58472.2	171.0	0.010476639	0.05899	0.22630	0.08173	0.03308
26	58869.3	169.9	0.007391176	0.04133	-0.04311	-0.16869	-0.10497
27	59092.0	169.2	0.007076640	0.03943	0.12510	-0.15387	0.00987
28	61484.6	162.6	0.096122074	0.51467	-0.36398	0.19634	-0.58621
29	60599.3	165.0	0.020427841	0.11098	0.14115	0.21099	0.21572
30	61936.5	161.5	0.009981573	0.05306	-0.18001	0.11037	-0.09203
31	62866.5	159.1	0.165269463	0.86546	0.52843	0.46379	0.60920
32	61867.4	161.6	0.014652388	0.07797	0.06344	0.26869	0.04181
33	63823.3	156.7	0.010546321	0.05440	-0.20883	-0.06007	0.08475
34	65146.1	153.5	0.047773065	0.24142	0.23401	0.40355	0.15430

35	62936.9	158.9	0.163611579	0.85582	0.57848	0.50049	0.52028
36	65301.9	153.1	0.018235948	0.09193	0.04571	0.21415	-0.20972
37	61693.0	162.1	0.038790875	0.20700	0.02756	0.44405	-0.09519
38	63804.9	156.7	0.051292737	0.26465	-0.05652	0.38574	-0.33566
39	65536.2	152.6	0.037032896	0.18603	-0.15041	0.38436	-0.12521
40	66815.3	149.7	0.007651483	0.03770	0.07728	-0.07836	0.15996
41	67083.6	149.1	0.054998675	0.26991	-0.03038	0.45444	-0.24993
42	66747.2	149.8	0.010853770	0.05353	0.17245	0.14096	-0.06263
43	67947.4	147.2	0.008624271	0.04179	0.13437	0.15404	-0.00049
44	68091.5	146.9	0.056297101	0.27219	-0.18814	0.23485	-0.42619
45	68259.1	146.5	0.033358608	0.16089	0.10766	0.33796	-0.18729
46	68613.2	145.7	0.061485524	0.29501	-0.02649	0.54206	0.02188
47	68635.7	145.7	0.005729266	0.02748	0.05075	-0.09379	-0.12692
48	68879.2	145.2	0.037378303	0.17865	-0.18177	-0.38019	0.03266
49	69721.4	143.4	0.080309951	0.37921	-0.39985	-0.09674	-0.45823
50	69499.3	143.9	0.014882278	0.07050	-0.10026	0.08048	-0.23231

ABSORPTION SPECTRUM VIA TRANSITION VELOCITY DIPOLE MOMENTS

State	Energy (cm-1)	hv (nm)	fosc	P2 (au**2)	PX (au)	PY (au)	PZ (au)
1	28367.8	352.5	0.021759569	0.00422	0.02041	0.06126	0.00706
2	31599.1	316.5	0.023585529	0.00509	-0.02977	-0.06465	0.00523
3	37298.1	268.1	0.007612726	0.00194	-0.00409	-0.03846	0.02109
4	35115.4	284.8	0.001647935	0.00040	0.01259	0.00035	-0.01539
5	42443.2	235.6	0.014084407	0.00409	0.00397	-0.01955	0.06073
6	44919.2	222.6	0.000341182	0.00010	0.00412	0.00484	0.00802
7	46772.0	213.8	0.073021415	0.02334	-0.07496	0.00170	-0.13312
8	46608.7	214.6	0.027428237	0.00874	-0.04665	0.00793	-0.08061
9	46789.5	213.7	0.001846096	0.00059	0.01694	0.00946	0.01463
10	48771.0	205.0	0.023436475	0.00781	0.03628	-0.02689	0.07598
11	49775.6	200.9	0.003240478	0.00110	-0.01807	0.01456	-0.02375
12	50366.2	198.5	0.020183459	0.00695	0.02747	-0.02429	0.07485
13	52406.9	190.8	0.012061205	0.00432	-0.03050	-0.00332	-0.05813
14	46861.0	213.4	0.001378657	0.00044	0.01935	-0.00546	0.00611
15	52859.3	189.2	0.004096666	0.00148	0.00754	0.00122	0.03770
16	53206.4	187.9	0.011051925	0.00402	0.02700	0.00362	0.05724
17	52444.5	190.7	0.000508221	0.00018	0.00514	-0.01247	-0.00037
18	55125.1	181.4	0.009099795	0.00343	0.02587	-0.01236	0.05105

19	55378.6	180.6	0.003011910	0.00114	0.01944	0.01551	0.02283
20	55704.6	179.5	0.004297060	0.00164	-0.02611	0.00542	-0.03041
21	56032.8	178.5	0.009768333	0.00374	-0.03471	-0.00389	-0.05021
22	57350.3	174.4	0.000934123	0.00037	0.01903	0.00200	0.00023
23	58507.1	170.9	0.002009293	0.00080	-0.00156	-0.02625	0.01059
24	57055.1	175.3	0.001108461	0.00043	0.01675	0.00403	0.01163
25	58472.2	171.0	0.003522755	0.00141	-0.03443	0.00400	-0.01436
26	58869.3	169.9	0.001516472	0.00061	-0.00183	0.00896	0.02295
27	59092.0	169.2	0.000600878	0.00024	-0.01183	0.00747	0.00685
28	61484.6	162.6	0.024971237	0.01049	0.03576	-0.02996	0.09119
29	60599.3	165.0	0.005454405	0.00226	-0.00698	-0.00818	-0.04630
30	61936.5	161.5	0.001879484	0.00080	0.01523	-0.02050	0.01197
31	62866.5	159.1	0.024680715	0.01060	-0.05030	-0.01439	-0.08870
32	61867.4	161.6	0.003120637	0.00132	-0.00713	-0.03562	0.00032
33	63823.3	156.7	0.002015190	0.00088	0.00154	-0.02036	-0.02150
34	65146.1	153.5	0.008648941	0.00385	-0.04599	-0.01765	-0.03774
35	62936.9	158.9	0.020112677	0.00865	-0.06456	-0.01109	-0.06603
36	65301.9	153.1	0.007879633	0.00352	0.00814	-0.01456	0.05691
37	61693.0	162.1	0.003455229	0.00146	0.00982	-0.03333	0.01580
38	63804.9	156.7	0.008301343	0.00362	0.02060	-0.01665	0.05402
39	65536.2	152.6	0.010745771	0.00481	0.03949	-0.05608	0.01041
40	66815.3	149.7	0.005463613	0.00249	-0.03442	0.00918	-0.03501
41	67083.6	149.1	0.013904659	0.00638	0.05716	-0.03498	0.04341
42	66747.2	149.8	0.007393259	0.00337	-0.04045	0.02677	0.03194
43	67947.4	147.2	0.001966731	0.00091	-0.02504	-0.01693	0.00014
44	68091.5	146.9	0.020422288	0.00950	0.05369	-0.01864	0.07921
45	68259.1	146.5	0.004820999	0.00225	-0.01792	-0.03267	0.02934
46	68613.2	145.7	0.009348895	0.00438	0.02312	-0.05599	-0.02674
47	68635.7	145.7	0.001978872	0.00093	-0.01089	0.00924	0.02691
48	68879.2	145.2	0.003695484	0.00174	0.01136	0.03594	-0.01786
49	69721.4	143.4	0.021201081	0.01010	0.06095	-0.01540	0.07842
50	69499.3	143.9	0.004202093	0.00200	0.01730	-0.02085	0.03552

CD SPECTRUM

State	Energy (cm-1)	hv (nm)	R (1e40*cgs)	MX (au)	MY (au)	MZ (au)
1	28367.8	352.5	-28.93212	-0.49366	-0.02794	0.16022
2	31599.1	316.5	33.57711	0.11993	-0.22707	-0.24264
3	37298.1	268.1	-15.50648	0.34048	-0.10611	-0.16108
4	35115.4	284.8	10.06194	0.05907	0.07512	0.02287
5	42443.2	235.6	28.06438	-0.07250	0.01068	-0.01862
6	44919.2	222.6	-11.43259	-0.12031	-0.06337	0.09658
7	46772.0	213.8	-40.90031	0.31511	0.00919	-0.24672
8	46608.7	214.6	-13.88648	0.12474	-0.11381	-0.14025
9	46789.5	213.7	-39.58851	0.14954	-0.00579	0.21796
10	48771.0	205.0	131.96572	-0.39743	-0.06331	-0.08193
11	49775.6	200.9	-23.41912	-0.09315	-0.03571	-0.03452
12	50366.2	198.5	-1.01257	-0.08131	0.01790	0.05880
13	52406.9	190.8	-28.19288	0.07852	0.00037	-0.18292
14	46861.0	213.4	-13.46924	0.12105	0.00350	0.07394
15	52859.3	189.2	25.90597	-0.32712	0.21365	0.15793
16	53206.4	187.9	-13.28600	-0.02639	0.14684	0.10319
17	52444.5	190.7	11.04891	0.20238	0.12215	-0.08159
18	55125.1	181.4	28.39877	-0.06061	-0.07552	-0.02499
19	55378.6	180.6	14.61971	0.30639	0.02567	-0.10597
20	55704.6	179.5	3.80750	-0.04001	0.18767	0.03853
21	56032.8	178.5	-39.03416	0.28326	0.05880	-0.16641
22	57350.3	174.4	1.37962	-0.08244	-0.16889	-0.07900
23	58507.1	170.9	-7.13283	0.30409	-0.00530	-0.19958
24	57055.1	175.3	-0.46367	0.00732	0.03078	-0.01107
25	58472.2	171.0	-22.11552	-0.25392	0.09165	0.09261
26	58869.3	169.9	0.68017	0.12961	-0.01642	-0.04059
27	59092.0	169.2	6.50755	0.06517	-0.04798	-0.17543
28	61484.6	162.6	1.01363	-0.08498	-0.02399	0.04106
29	60599.3	165.0	18.18129	-0.05103	0.03561	0.17733
30	61936.5	161.5	-8.24813	0.04305	0.00554	0.11255
31	62866.5	159.1	6.79816	-0.09638	-0.03819	0.13634
32	61867.4	161.6	-25.05855	-0.19716	-0.12716	-0.15491
33	63823.3	156.7	-20.19048	0.22911	-0.12302	-0.02798

34	65146.1	153.5	12.92354	-0.30532	0.18329	0.16134
35	62936.9	158.9	39.81466	-0.01635	0.10618	0.07836
36	65301.9	153.1	-2.15124	0.17170	-0.16108	-0.10530
37	61693.0	162.1	22.82194	0.04931	0.08782	-0.08458
38	63804.9	156.7	63.44005	-0.02999	0.14732	-0.22654
39	65536.2	152.6	-1.10205	0.06488	0.03820	0.05801
40	66815.3	149.7	10.46302	0.13875	-0.08310	0.03100
41	67083.6	149.1	-31.65213	0.03136	-0.11910	0.04826
42	66747.2	149.8	-8.86588	0.00463	-0.09606	0.09682
43	67947.4	147.2	-4.01069	-0.03877	-0.02181	-0.12731
44	68091.5	146.9	-24.92579	-0.21807	-0.23943	0.08838
45	68259.1	146.5	68.50971	0.16154	0.30966	-0.12426
46	68613.2	145.7	-16.20448	0.19255	-0.05558	0.03926
47	68635.7	145.7	-18.76347	0.22554	0.14451	0.29697
48	68879.2	145.2	3.20317	0.03473	-0.02966	0.05607
49	69721.4	143.4	-32.22334	0.01513	-0.12180	0.16167
50	69499.3	143.9	11.90377	-0.05584	-0.16359	-0.14126

9. References

[1] a) C. Raminelli, J. V. Comasseto, L. H. Andrade, A. L. M. Porto, *Tetrahedron: Asymmetry* **2004**, *15*, 3117-3122; b) D. Xu, Z. Li, S. Ma, *Tetrahedron Lett.* **2003**, *44*, 6343-6346.

[2] Y. C. Fan, O. Kwon, *Org. Lett.* **2015**, *17*, 2058-2061.

[3] a) S. Özçubukçu, E. Ozkal, C. Jimeno, M. A. Pericàs, *Org. Lett.* **2009**, *11*, 4680-4683; b) E. Ozkal, P. Llanes, F. Bravo, A. Ferrali, M. A. Pericàs, *Adv. Synth. Catal.* **2014**, *356*, 857-869.

[4] C. Hempel, C. Maichle-Mössmer, M. A. Pericàs, B. J. Nachtsheim, *Adv. Synth. Catal.* **2017**, *359*, 2896-2896.

[5] Q.-B. Zhang, Y.-L. Ban, D.-G. Zhou, P.-P. Zhou, L.-Z. Wu, Q. Liu, *Org. Lett.* **2016**, *18*, 5256-5259.

[6] A. K. Chakraborti, S. V. Chankeshwara, *J. Org. Chem.* **2009**, *74*, 1367-1370.

[7] C. Bannwarth, S. Ehlert, S. Grimme, *J. Chem. Theory. Comput.* **2019**, *15*, 1652-1671.

[8] S. Grimme, J. G. Brandenburg, C. Bannwarth, A. Hansen, *J. Phys. Chem.* **2015**, *143*, 054107.

[9] A. V. Marenich, C. J. Cramer, D. G. Truhlar, *J. Phys. Chem. B* **2009**, *113*, 6378-6396.

[10] F. Weigend, *Phys. Chem. Chem. Phys.* **2006**, *8*, 1057-1065.

[11] L. Goerigk, S. Grimme, *J. Chem. Theory. Comput.* **2011**, *7*, 291-309.

[12] A. Hellweg, C. Hättig, S. Höfener, W. Klopper, *Theor. Chem. Acc.* **2007**, *117*, 587-597.
